# Distinct HIF1**α** and HIF2**α** functions control skeletal muscle metabolism and erythropoiesis

**DOI:** 10.1172/JCI195411

**Published:** 2026-02-17

**Authors:** Junhyeong Lee, Merc Emil Matienzo, Sangyi Lim, Edzel Evallo, Yeongsin Kim, Sujin Jang, Keon Kim, Chang Hyeon Choi, Youn Ho Han, Chang-Min Lee, Tae-Il Jeon, Sang-Ik Park, Jun Wu, Dong-il Kim, Min-Jung Park

**Affiliations:** 1 Department of Veterinary Physiology, College of Veterinary Medicine,; 2 College of Veterinary Medicine and BK21 FOUR Program, and; 3 Department of Veterinary Internal Medicine, College of Veterinary Medicine, Chonnam National University, Gwangju, South Korea.; 4 Department of Oral Pharmacology, College of Dentistry, Wonkwang University, Iksan, South Korea.; 5 Department of Animal Science and; 6 Department of Veterinary Pathology, College of Veterinary Medicine, Chonnam National University, Gwangju, South Korea.; 7 Department of Molecular and Integrative Physiology, University of Michigan Medical School, Ann Arbor, Michigan, USA.; 8 Life Sciences Institute, University of Michigan, Ann Arbor, Michigan, USA.

**Keywords:** Metabolism, Muscle biology, Hypoxia, Mitochondria, Muscle

## Abstract

Skeletal muscle frequently encounters hypoxic stress, particularly during exercise, but the specific functions of the hypoxia-inducible factors HIF1α and HIF2α within myofibers remain unclear due to the lack of appropriate in vivo models. Here, we generated 3 complementary mouse models, myofiber-specific triple-PHD knockout (PHD mTKO) and inducible myofiber-specific overexpression of stabilized HIF1α or HIF2α, to delineate isoform-specific roles of HIFα signaling in skeletal muscle. HIF1α stabilization increased the proportion of oxidative fibers yet paradoxically impaired exercise capacity and mitochondrial function. In contrast, HIF2α activation protected against diet-induced obesity, improved glucose tolerance, and maintained mitochondrial function without altering fiber-type composition. Notably, HIF2α stabilization markedly elevated erythropoietin (EPO) expression in skeletal muscle and serum. Myofiber-specific deletion of EPO in the PHD mTKO background abolished polycythemia, demonstrating that this phenotype is driven specifically by muscle-derived EPO. Together, these findings uncover distinct roles of HIF1α and HIF2α in regulating muscle metabolism and mitochondrial function and establish the PHD–HIF2α axis as a myofiber-derived driver of systemic EPO production.

## Abstract 2

HIF2α in skeletal muscle improves glucose tolerance and induces erythropoiesis

## Introduction

Hypoxia-inducible factor alpha (HIFα) is a key transcription factor activated by oxygen deficiency, which causes transient hypoxia. HIF1α and HIF2α, the major isoforms of HIFα, modulate common and unique target genes involved in glucose metabolism, mitochondrial function, angiogenesis, and erythropoiesis (1–4). The regulation of HIFα stabilization in response to oxygen availability is a well-established mechanism. Under normal oxygen conditions, HIFα is hydroxylated on specific proline residues by three prolyl hydroxylase proteins (PHD1, PHD2, and PHD3, encoded by *Egln2*, *Egln1*, and *Egln3*) using oxygen as a substrate. This modification promotes the von Hippel-Lindau (VHL) protein complex to bind, which is an E3 ubiquitin ligase, leading to HIFα becoming ubiquitinated and subsequently undergoing proteasomal degradation (5, 6). Under hypoxic conditions, due to the absence of an oxygen substrate, HIFα hydroxylation is limited, subsequently increasing its stability and activating downstream pathways.

Skeletal muscle is highly metabolically active and relies heavily on oxygen during physical activity. Oxygen deficiency frequently arises in skeletal muscle under various conditions, including high-intensity exercise or sustained physical exertion (7–9). Since oxidation is the primary source of adenosine triphosphate (ATP) for muscle contractions, a reduction in oxygen availability can compromise skeletal muscle homeostasis (10). Despite the critical association between oxygen availability and skeletal muscle, the role of HIFα and the PHDs in skeletal muscle remains unclear.

Here, we generated myofiber-specific PHD triple knockout mice lacking all 3 PHDs to evaluate the impact of HIFα stabilization in skeletal muscle. We demonstrated that a deficiency in all PHDs in skeletal muscle confers resistance to weight gain and glucose intolerance. These PHD deficiencies also increase the proportion of oxidative muscle fibers but counterintuitively impair exercise performance and mitochondrial function. By transducing normoxia-stable HIF1α or HIF2α into myofibers, we further demonstrated that HIF1α primarily regulates the distribution of oxidative muscle fibers and mitochondrial function; however, HIF2α mainly enhances glucose tolerance and promotes weight loss. Surprisingly, HIF2α stabilization, but not HIF1α, in myofibers markedly elevated erythropoietin (EPO) levels in both blood and muscle, subsequently expanding hematocrit levels and mortality. This study demonstrated the separate roles of HIF1α and HIF2α in skeletal muscle, highlighting that the PHD–HIF2α axis produces EPO from myofibers.

## Results

### PHD deficiencies in skeletal muscle confer resistance to glucose intolerance and diet-induced obesity.

HIFα stabilization is highly regulated by the oxygen-sensing PHD enzymes PHD1, PHD2, and PHD3. Western blot analysis revealed that PHD proteins are expressed in the skeletal muscles of C57BL/6 mice, with PHD1 and PHD2 levels being significantly higher in the soleus compared with the extensor digitorum longus (Supplemental Figure 1A; supplemental material available online with this article; https://doi.org/10.1172/JCI195411DS1). Using Myoatlas, a public database of single-nucleus RNA-seq in skeletal muscle (https://research.cchmc.org/myoatlas) (11), we found that all 3 genes encoding the 3 PHD isotypes are sufficiently expressed in myocytes (Supplemental Figure 1B). To investigate the specific role of each PHD isoform in regulating HIFα protein levels in skeletal muscle, we generated myofiber-specific single-PHD knockout (PHD mKO) mice by crossing *Phd1^fl/fl^*, *Phd2^fl/fl^*, or *Phd3^fl/fl^* mice with HSA-Cre mice, which express Cre-recombinase under the control of a human *ACTA1* promoter, driving expression specifically in skeletal muscle but not in the heart (Supplemental Figure 2, A–C) (12, 13). The single-PHD mKO mice did not show increased HIF1α and HIF2α stability or elevated levels of classical HIFα target gene expression in skeletal muscles, although PHD1 deficiency tended to lead to increased HIFα protein levels that did not reach statistical significance, and no corresponding increase in target gene expression was observed (Supplemental Figure 2, D and E), suggesting that depletion of a single PHD alone is insufficient to stabilize HIFα.

**Figure 1:**
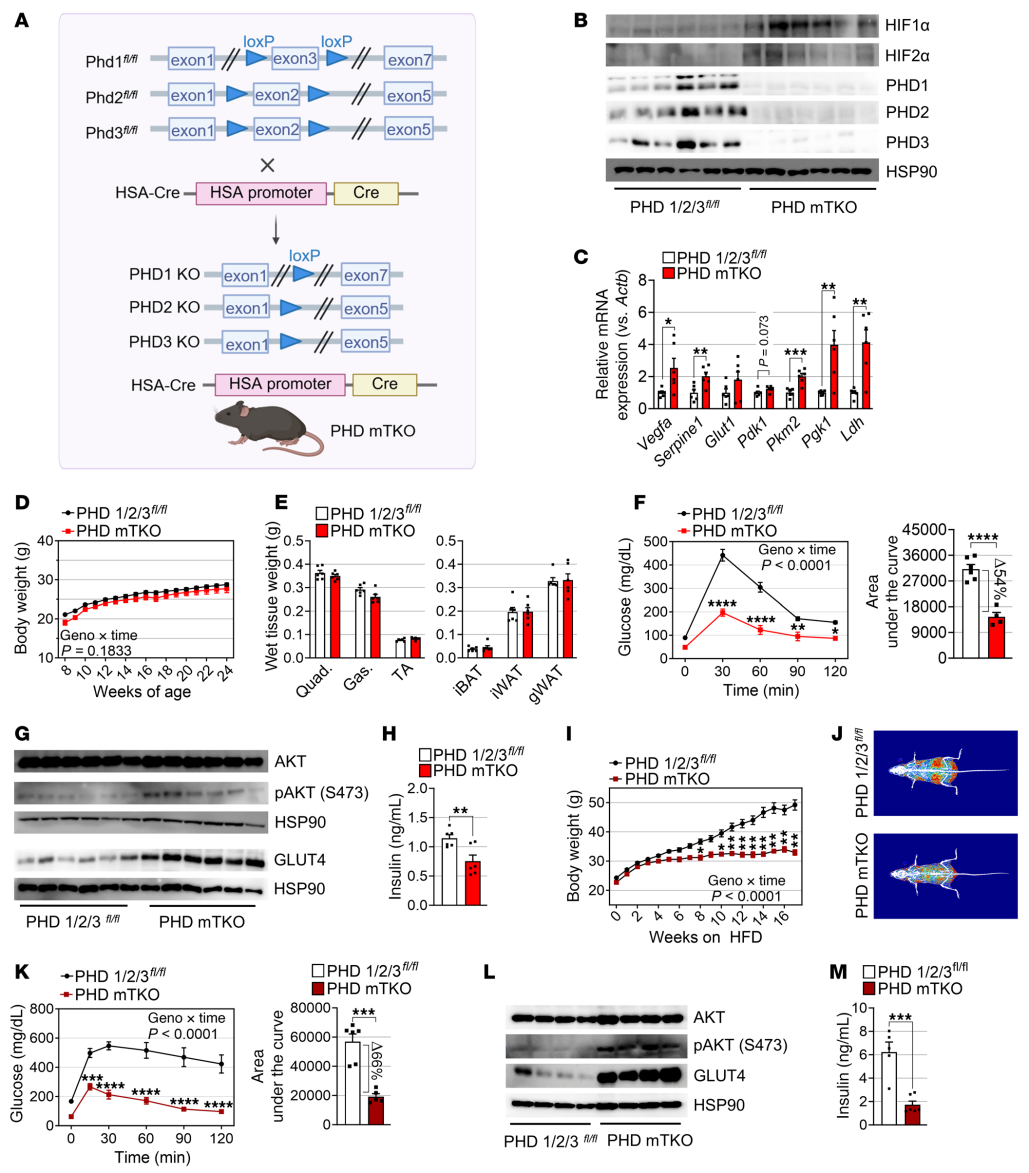
PHD deficiency in skeletal muscle protects against glucose intolerance and diet-induced obesity. (**A**) Schematic of the PHD mTKO mice. (**B**) Representative immunoblots of PHD1, PHD2, PHD3, HIF1α, and HIF2α from the soleus. (**C**) mRNA levels of HIFα target genes in the soleus (PHD 1/2/3*^fl/fl^*, *n* = 6; PHD mTKO, *n* = 6). (**D**) Body weights of mice on an NCD (PHD 1/2/3*^fl/fl^*, *n* = 9; PHD mTKO, *n* = 4). (**E**) Weights of muscles and adipose tissues of mice on an NCD (PHD 1/2/3*^fl/fl^*, *n* = 6; PHD mTKO, *n* = 6). (**F**) GTT on 21-week-old mice on an NCD (left) and area under the curve of glucose levels (right) (PHD 1/2/3*^fl/fl^*, *n* = 6; PHD mTKO, *n* = 4). (**G**) Representative immunoblots from the soleus. (**H**) Fasting serum insulin levels (PHD 1/2/3*^fl/fl^*, *n* = 6; PHD mTKO, *n* = 6). (**I**) Body weights of male mice on an HFD (PHD 1/2/3*^fl/fl^*, *n* = 6; PHD mTKO, *n* = 5). (**J**) Representative images of body composition; red areas indicate fat depots. (**K**) GTT on 14-week-old HFD-fed mice (left) and area under the curve of glucose levels (right) (PHD 1/2/3*^fl/fl^*, *n* = 6; PHD mTKO, *n* = 5). (**L**) Representative immunoblots from the soleus. (**M**) Fasting serum insulin levels (PHD 1/2/3*^fl/fl^*, *n* = 5; PHD mTKO, *n* = 6). Data are shown as the mean ± SEM. Unpaired 2-tailed Student’s *t* test (**C**, **E**, **F**, **H**, **K**, and **M**) or 2-way ANOVA with Bonferroni’s post hoc test (**D**, **F**, **I**, and **K**) was used for statistical analyses. *P < 0.05, **P < 0.01, ***P < 0.001, and ****P < 0.0001. Quad., quadriceps; Gas., gastrocnemius; TA, tibialis anterior; iBAT, interscapular brown adipose tissue; iWAT, inguinal white adipose tissue.

Accordingly, we developed mice with myofiber-specific triple deletion for PHD1, PHD2, and PHD3 (hereafter, PHD mTKO) (Figure 1A). We confirmed the specific ablation of PHDs in the muscle, but not in the heart, white adipose tissue, kidney, or liver (Figure 1B and Supplemental Figure 3A). We further verified that the expression levels of both HIF1α and HIF2α were significantly elevated in the muscles of PHD mTKO mice (Figure 1B and Supplemental Figure 3, B and C). The mRNA expression of classical HIFα target genes related to angiogenesis (*Vegfa*) (14), fibrinolysis inhibition (*Serpine1*) (15), and glucose metabolism (*Pgk1*, *Pkm2*, and *Ldh*) (16) was significantly elevated in the muscles of PHD mTKO mice (Figure 1C), indicating that the triple-PHD knockout increases HIFα stability and activity.

PHD mTKO mice showed body weights comparable with control mice under normal chow diet (NCD) conditions, with no significant differences observed in the weights of skeletal muscles (quadriceps, gastrocnemius, and tibialis anterior) or adipose tissues (interscapular brown adipose tissue, inguinal white adipose tissue, and gonadal white adipose tissue [gWAT]) (Figure 1, D and E). The histological morphologies of the adipose tissues and livers also showed no apparent differences in fat accumulation levels between PHD mTKO and control littermates (Supplemental Figure 4, A and B). However, PHD mTKO mice exhibited a significantly lower nonfasted blood glucose level and enhanced glucose tolerance compared with the control group (~54% decrease in the glucose level determined by area under the curve) (Figure 1F and Supplemental Figure 4C). Furthermore, we observed higher levels of phosphorylated AKT and GLUT4 in the muscles of PHD mTKO mice compared with littermate controls, suggesting enhanced activation of insulin signaling and glucose uptake, which was further supported by a concomitant decrease in serum insulin level (Figure 1, G and H, and Supplemental Figure 4D). In contrast, other metabolic tissues, such as adipose tissue and liver, did not exhibit this trend, suggesting that enhanced insulin signaling in PHD mTKO mice is specific to skeletal muscle (Supplemental Figure 4, E and F). It was previously reported that HIFα induces fatty acid uptake and lipid storage (17, 18). Although the expression of genes involved in these processes was altered, both intramuscular and serum free fatty acid (FFA) levels remained unchanged (Supplemental Figure 4, G–I).

Next, we examined whether PHD deficiency in skeletal muscle protects against diet-induced obesity. While the body weights of mice on an NCD did not differ between groups, PHD mTKO mice gained significantly less weight than the littermate controls that were administered a high-fat diet (HFD) (Figure 1I). Notably, the lower body weight of PHD mTKO mice was primarily due to a marked reduction in fat mass (Figure 1J and Supplemental Figure 5, A–C). Lipid accumulation in the liver and adipose tissues was also reduced in PHD mTKO mice, and this was accompanied by a noticeable decrease in gWAT adipocyte size (Supplemental Figure 5, D and E). Furthermore, glucose tolerance was remarkably improved, accompanied by increased GLUT4 expression and AKT phosphorylation, in PHD mTKO mice (Figure 1, K and L, and Supplemental Figure 5F). Similar to observations under NCD conditions, the HFD-induced elevation in serum insulin levels was drastically reduced in PHD mTKO mice (Figure 1M). However, no changes were detected in intramuscular FFA levels and in serum FFA concentrations (Supplemental Figure 5, G and H). Circulating leptin levels were also lower in PHD mTKO mice, likely due to their reduced adiposity (Supplemental Figure 5I).

Compared with males, females exhibited distinct metabolic profiles in skeletal muscle and differential fat accumulation. Therefore, we investigated whether the effects of skeletal muscle PHD deficiency on glucose metabolism and obesity resistance were reproducible in female mice and found that female PHD mTKO mice exhibited metabolic phenotypes largely comparable with those of males (Supplemental Figure 6). These data indicate that skeletal muscle–specific deletion of PHD enzymes increases HIF1α and HIF2α stability and improves glucose tolerance and diet-induced obesity in both male and female mice.

### PHD deficiencies in skeletal muscle increase the oxidative fiber ratio.

**Figure 2:**
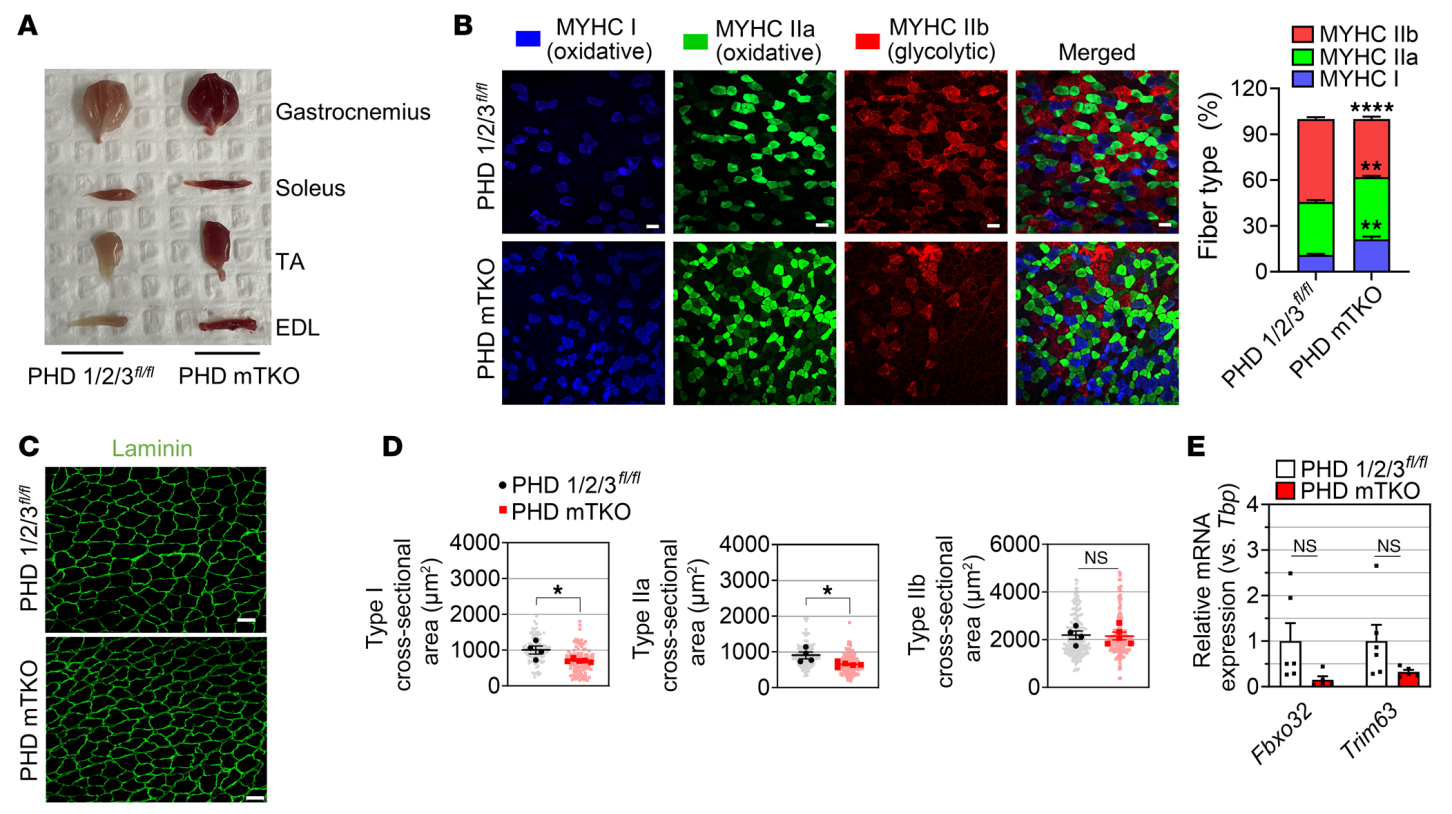
PHD deficiency in skeletal muscle increases oxidative fiber ratio. (**A**) Skeletal muscles isolated from a 6-month-old PHD mTKO mouse and its littermate. (**B**) Immunofluorescence staining for MYHC I (blue), MYHC IIa (green), and MYHC IIb (red) from the gastrocnemius (left). The ratio of each myofiber type composition (PHD 1/2/3*^fl/fl^*, *n* = 4; PHD mTKO, *n* = 5; right). Scale bars = 50 μm. (**C**) Immunofluorescence staining for laminin from the gastrocnemius. Scale bars = 50 μm. (**D**) The cross-sectional area of each myofiber from **B** (PHD 1/2/3*^fl/fl^*, *n* = 4; PHD mTKO, *n* = 5; right). (**E**) mRNA expression levels of muscle atrophic genes (PHD 1/2/3*^fl/fl^*, *n* = 6; PHD mTKO, *n* = 6). Data are shown as the mean ± SEM. Unpaired 2-tailed Student’s *t* test was used for statistical analyses (**B**, **D**, and **E**). *P < 0.05, **P < 0.01, and ****P < 0.0001.

The functional characteristics of skeletal muscles are largely determined by the composition of myofibers, which are broadly classified into slow oxidative (type I), fast oxidative (type IIa), and fast glycolytic (type IIb) fibers. These classifications are based on the inherent differences in contraction velocity determined by myosin-ATPase activity and the primary source of ATP production (19). The latter factor, oxidative versus glycolytic, depends on mitochondrial content and metabolic properties. Given that a higher proportion of oxidative fibers are strongly associated with a lower risk of obesity and type 2 diabetes (20, 21), we hypothesized that the portion of oxidative fibers in PHD mTKO skeletal muscle might be increased. From a morphological perspective, we observed that the muscles in PHD mTKO mice appeared remarkably red (Figure 2A), a feature characteristic of an abundance of oxidative fibers (22). Immunofluorescence staining for myosin-heavy chains revealed that the abundance of type I and IIa fibers was relatively higher in the gastrocnemius of PHD mTKO mice, whereas type IIb fibers were reduced (Figure 2B). In agreement with this, the oxidative myosin heavy chain (MYH7) protein level was significantly increased in the gastrocnemius of PHD mTKO mice (Supplemental Figure 7A). The mRNA expression levels of glycolytic fiber–specific genes were significantly decreased, whereas oxidative fiber–specific markers tended to increase in the gastrocnemius and soleus (Supplemental Figure 7, B and C). Intriguingly, the myofibers in PHD mTKO mice exhibited a markedly reduced cross-sectional area compared with those in littermate controls without reduced muscle weights (Figure 1E and Figure 2, C and D). This reduction was primarily due to the decreased sizes of oxidative type I and type IIa fibers, without increasing the expression of muscle atrophy–related E3 ligase genes (*Fbxo32* and *Trim63*), indicating that the reduced fiber size was not attributable to muscle atrophy (Figure 2, D and E) (23).

### PHD deficiencies in skeletal muscle impair exercise performance and mitochondrial oxidative phosphorylation.

**Figure 3:**
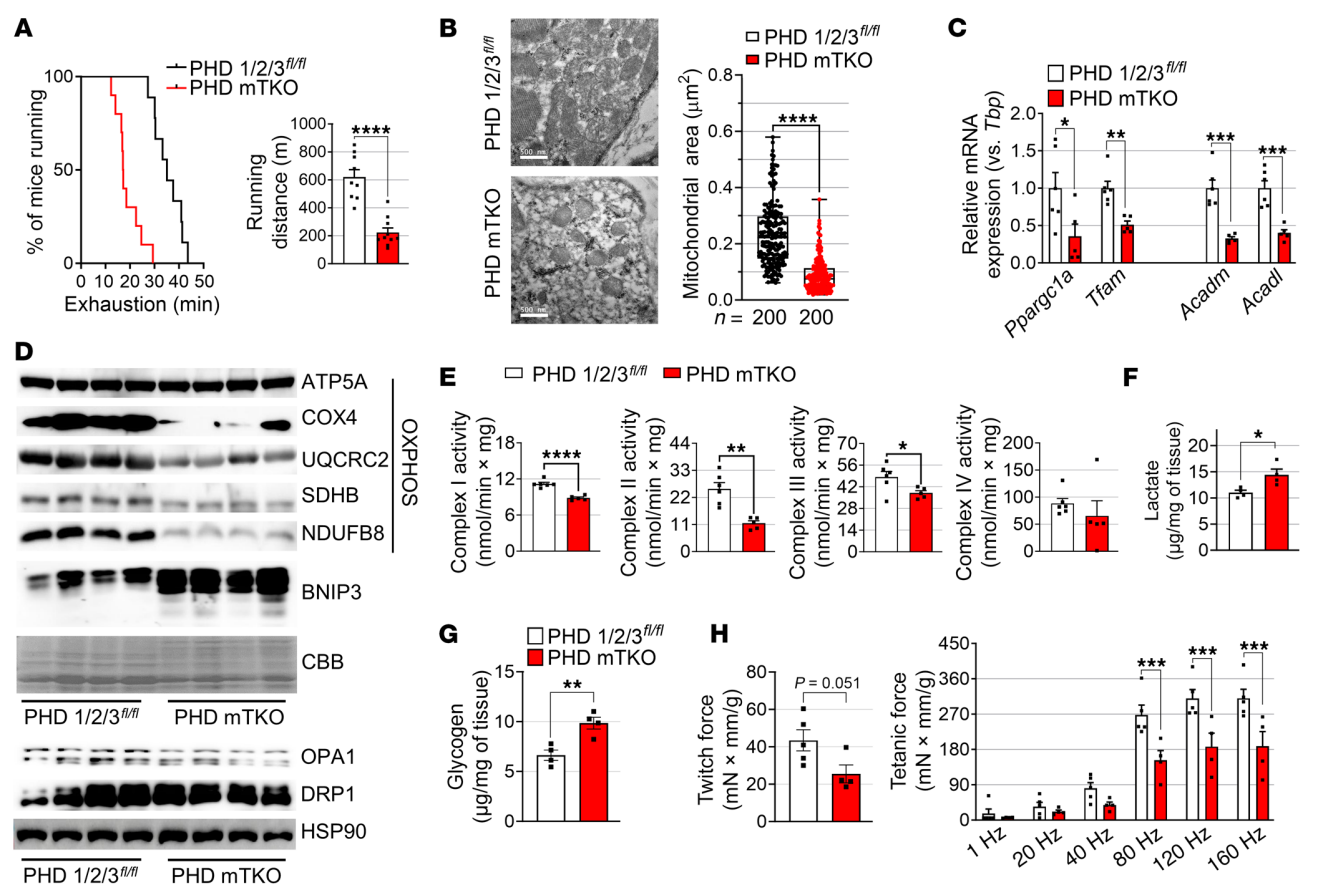
PHD deficiency in skeletal muscle impairs exercise endurance and mitochondrial function. (**A**) Percentage of mice running before exhaustion (left) and running distance (right) (PHD 1/2/3*^fl/fl^*, *n* = 9; PHD mTKO, *n* = 10). (**B**) Transmission electron microscopy images of the gastrocnemius (left) and measurement of the mitochondrial area (right). Scale bars = 500 μm. (**C**) mRNA expression levels of indicated genes (PHD 1/2/3*^fl/fl^*, *n* = 6; PHD mTKO, *n* = 5). (**D**) Immunoblot of the soleus for the indicated proteins (PHD 1/2/3*^fl/fl^*, *n* = 4; PHD mTKO, *n* = 4). (**E**) Enzymatic activities of the mitochondrial complexes (PHD 1/2/3*^fl/fl^*, *n* = 6; PHD mTKO, *n* = 5). (**F** and **G**) Intramuscular lactate (**F**) and glycogen (**G**) content in the gastrocnemius (PHD 1/2/3*^fl/fl^*, *n* = 4; PHD mTKO, *n* = 4). (**H**) Twitch force and tetanic plantar flexion torque normalized to body weight (PHD 1/2/3*^fl/fl^*, *n* = 5; PHD mTKO, *n* = 4). Data are shown as the mean ± SEM. Unpaired 2-tailed Student’s *t* test (**A**–**C** and **E**–**H**) or 2-way ANOVA with Bonferroni’s post hoc test (**H**) was used for statistical analyses. *P < 0.05, **P < 0.01, ***P < 0.001, and ****P < 0.0001. CBB, Coomassie blue stain.

Given the increased number of oxidative fibers in the muscles of PHD mTKO mice, we anticipated that PHD deficiency in skeletal muscle would lead to improved exercise performance. However, it was unexpectedly found that the exercise performance on the treadmill by PHD mTKO mice was impaired (Figure 3A). Since ATP generated by oxidative phosphorylation (OXPHOS) is required for endurance exercise, we investigated the mitochondria of PHD mTKO mice. Quantitative transmission electron microscopy analysis revealed that PHD mTKO mice had significantly smaller mitochondria compared with littermate controls (Figure 3B). Consistent with this, BNIP3, an HIF1α target gene that contributes to mitochondrial fragmentation (24), was remarkably elevated, whereas the mitochondrial fusion factor OPA1 was decreased in PHD mTKO mice (Figure 3D and Supplemental Figure 7D). Moreover, mRNA and protein levels related to OXPHOS were markedly reduced in the muscles of PHD mTKO mice (Figure 3, C and D, and Supplemental Figure 7, D–I). The expression levels of genes associated with mitochondrial biogenesis (*Ppargc1a* and *Tfam*) and fatty acid oxidation (*Acadm* and *Acadl*) were also significantly decreased in the PHD mTKO mice (Figure 3C). Consistent with the gene and protein expression patterns, enzymatic activities of the mitochondrial complexes were significantly lower in the muscles of PHD mTKO mice; instead, lactate production and glycogen content were increased (Figure 3, E–G). In addition, both twitch and tetanic forces were decreased in the muscles of PHD mTKO mice (Figure 3H). These findings suggest that skeletal muscle PHD deficiency increases the proportion of oxidative fibers, yet paradoxically suppresses mitochondrial OXPHOS and impairs muscle function.

### HIF1α in skeletal muscle does not confer resistance to obesity.

**Figure 4:**
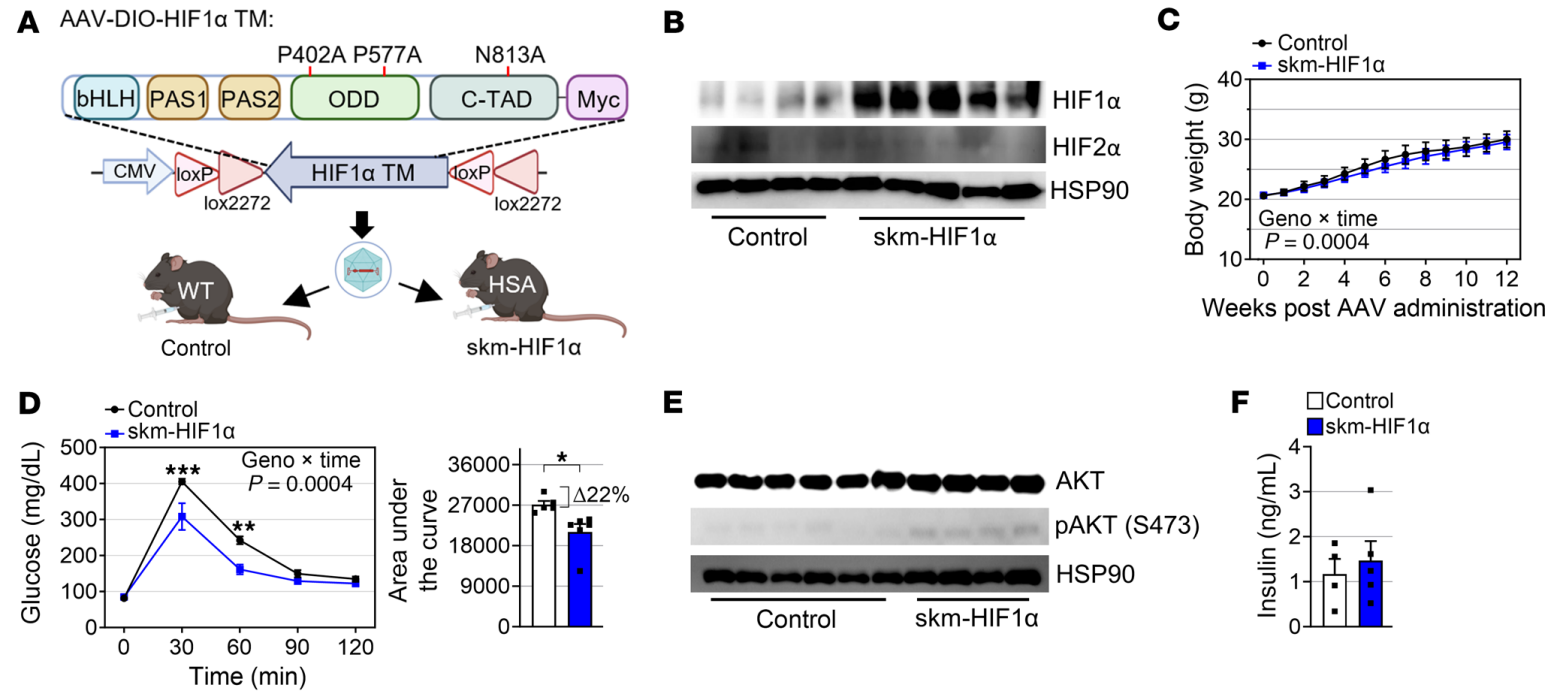
HIF1α in skeletal muscle marginally improves glucose tolerance. (**A**) Schematic of the myofiber-specific transduction of HIF1α TM and generation of skm-HIF1α mice. (**B**) Immunoblot analysis of the soleus confirmed the transduction of HIF1α (control, *n* = 4; skm-HIF1α, *n* = 5). (**C**) Body weights of male mice on an NCD (control, *n* = 5; skm-HIF1α, *n* = 6). (**D**) GTT on 18-week-old mice on an NCD (left) and area under the curve of glucose levels (right) (control, *n* = 5; skm-HIF1α, *n* = 6). (**E**) Representative immunoblots of AKT and pAKT from the soleus (control, *n* = 6; skm-HIF1α, *n* = 4). (**F**) Fasting serum insulin levels in mice (control, *n* = 4; skm-HIF1α, *n* = 5). Data are shown as the mean ± SEM. Unpaired 2-tailed Student’s *t* test (**D** and **F**) or 2-way ANOVA with Bonferroni’s post hoc test (**C** and **D**) was used for statistical analyses. *P < 0.05, **P < 0.01, and ***P < 0.001.

HIF1α and HIF2α are the main substrates of PHD enzymes (25). Since we confirmed the stabilization of both HIF1α and HIF2α in the skeletal muscles of PHD mTKO mice, we sought to determine which of these 2 HIFα isoforms is primarily responsible for the observed phenotypic changes. To address this, we designed parallel gain-of-function approaches for each isoform. As a first step, we generated mice with inducible HIF1α specifically in myofibers using the AAV-DIO system, which expresses transgenes in a Cre-dependent manner (26, 27). Due to the intrinsic instability and inactivation of HIF1α under normoxic conditions, we introduced a 3-point mutation (TM; HIF1α with P402A/P577A/N813A) to preserve stability in normoxia (Figure 4A) (28, 29). We primarily injected the AAV-DIO-HIF1α TM into HSA-Cre or WT mice as a control. HSA-Cre mice with AAV-DIO-HIF1α TM (hereafter, skm-HIF1α) exhibited increased expression levels of HIFα, to an extent comparable with that observed in PHD mTKO mice, and classical HIFα target genes in skeletal muscle, suggesting that the skm-HIF1α model was successfully generated (Figure 4B and Supplemental Figure 8, A–C).

Similar to the PHD mTKO mice, the skm-HIF1α mice also exhibited comparable body weights with the control group under NCD conditions without reductions occurring in muscle weights (Figure 4C and Supplemental Figure 8D). Glucose tolerance was slightly enhanced in skm-HIF1α mice; however, this trend was notably less pronounced than in the PHD mTKO mice (~54% decrease in glucose level in PHD mTKO mice vs. ~22% decrease in skm-HIF1α mice) (Figure 1F and Figure 4D). Although serum insulin levels were unchanged, AKT phosphorylation was increased in skm-HIF1α mice, suggesting that HIF1α enhances muscle insulin sensitivity (Figure 4, E and F, and Supplemental Figure 8E). In terms of fatty acid uptake and storage, despite the reduced expression of *Fabp3*, which is involved in fatty acid transport, the levels of intramuscular and serum FFAs remained unchanged in skm-HIF1α mice, suggesting that muscle HIF1α is relatively less involved in regulating fatty acid metabolism (Supplemental Figure 8, F–H).

**Figure 5:**
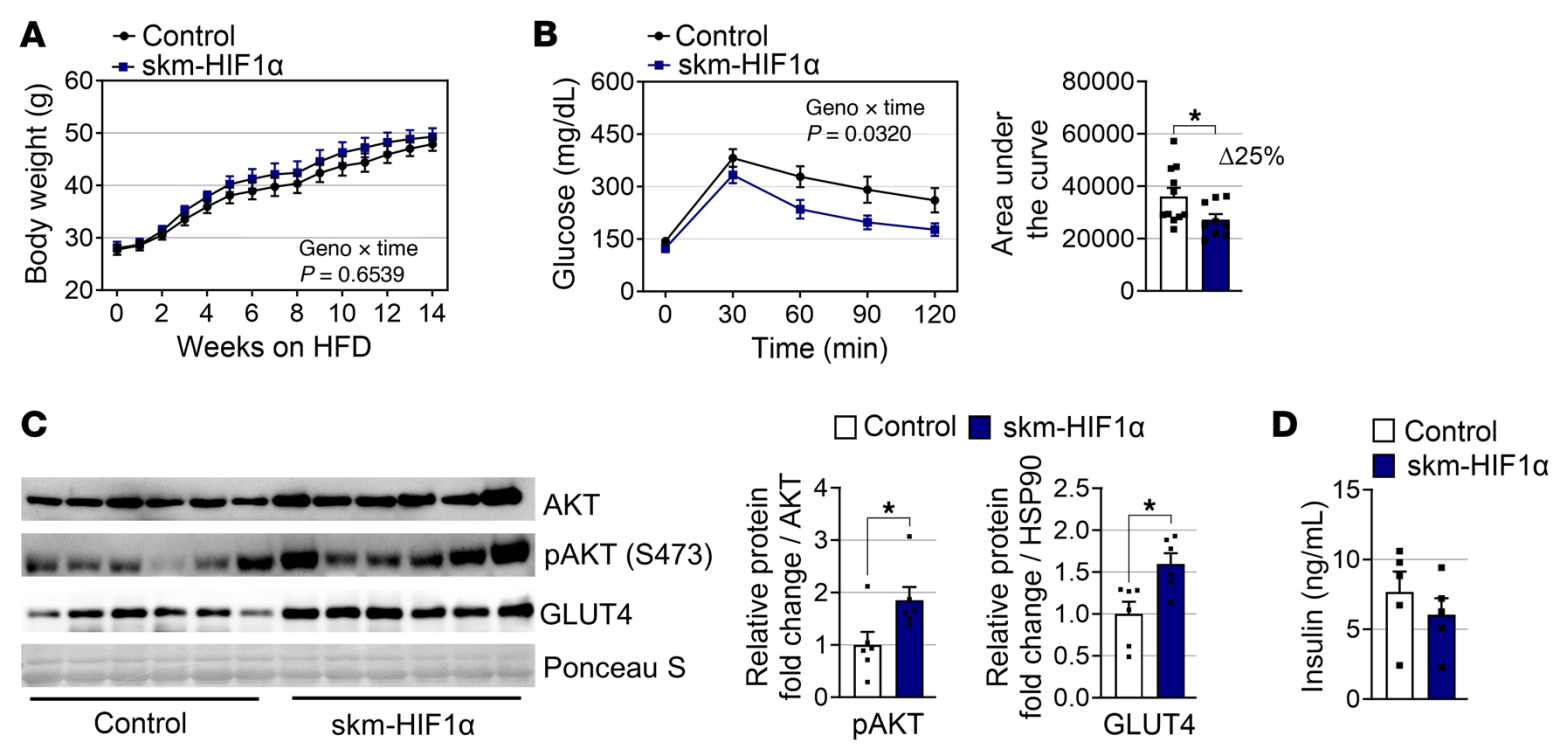
HIF1α in skeletal muscle does not confer resistance to obesity. (**A**) Body weights of male mice on an HFD (control, *n* = 9; skm-HIF1α, *n* = 4). (**B**) GTT on mice administered an HFD for 12 weeks (left) and area under the curve of glucose levels (right) (control, *n* = 11; skm-HIF1α, *n* = 8). (**C**) Representative AKT, pAKT, and GLUT4 immunoblots from the soleus (left) and quantification of indicated proteins (right) (control, *n* = 6; skm-HIF1α, *n* = 6). (**D**) Fasting serum insulin levels in mice (control, *n* = 5; skm-HIF1α, *n* = 5). Data are shown as the mean ± SEM. Unpaired 2-tailed Student’s *t* test (**B**–**D**) or 2-way ANOVA with Bonferroni’s post hoc test (**A** and **B**) was used for statistical analyses. *P < 0.05.

Unlike PHD mTKO mice, HFD-induced weight gain was comparable between skm-HIF1α and control mice (Figure 5A). While muscle weights remained unchanged, gWAT weight was slightly increased in skm-HIF1α; however, this increase was not accompanied by changes in adipocyte sizes (Supplemental Figure 8, I–K). Despite unchanged resistance against diet-induced obesity, glucose tolerance was enhanced in skm-HIF1α mice. However, this tendency remained less pronounced than in PHD mTKO mice (Figure 1K and Figure 5B). Meanwhile, increased GLUT4 and AKT phosphorylation levels in skm-HIF1α mice were further confirmed, without change in serum insulin levels (Figure 5, C and D).

### HIF1α in skeletal muscle increases the oxidative fiber ratio but impairs exercise performance.

**Figure 6:**
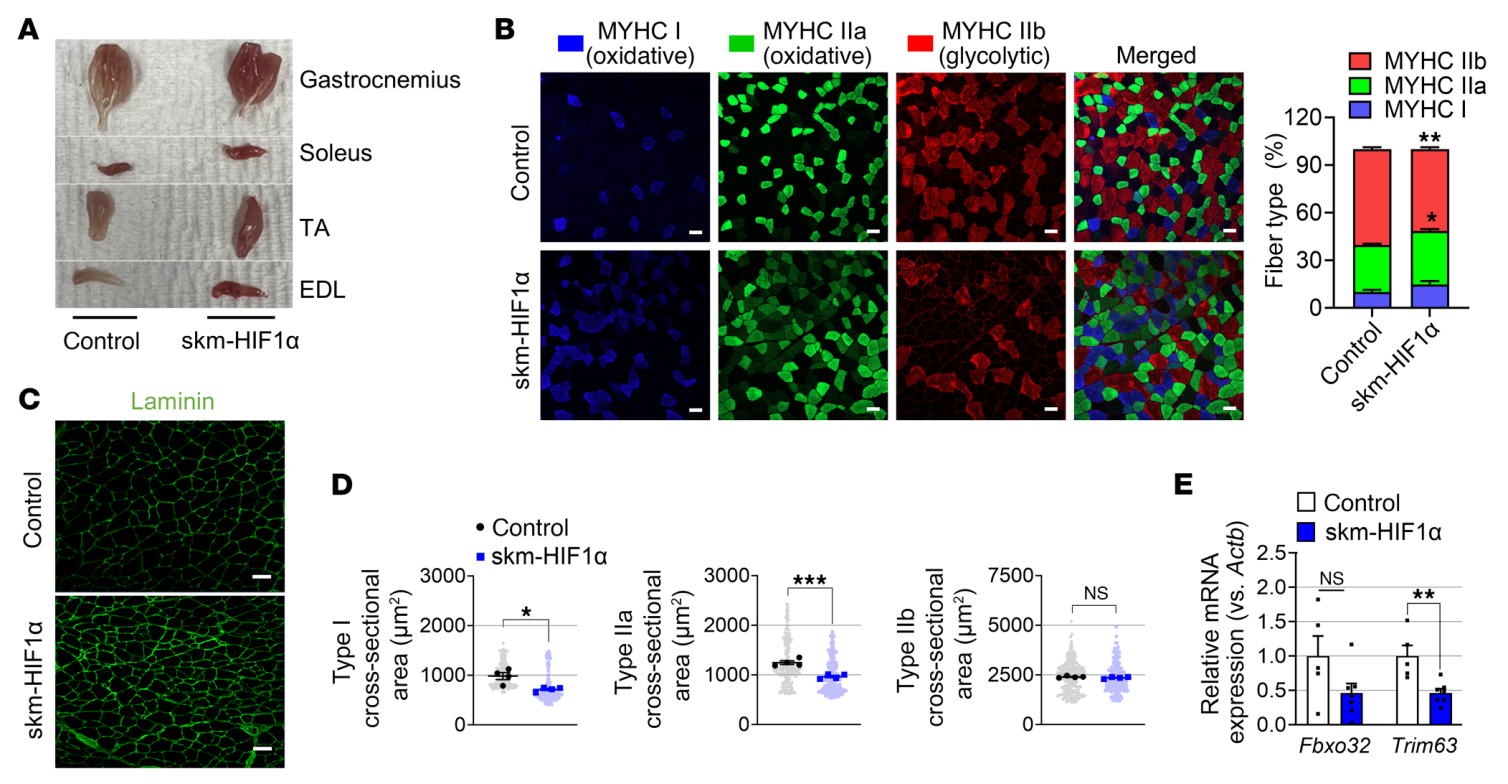
HIF1α in skeletal muscle increases the oxidative fiber ratio. (**A**) Skeletal muscles isolated from a 6-month-old skm-HIF1α mouse and a control mouse. (**B**) Representative images of immunofluorescence staining from the gastrocnemius (left) and the ratio of each myofiber type composition (right) (control, *n* = 4; skm-HIF1α, *n* = 4). Scale bars = 50 μm. (**C**) Representative images of immunofluorescence staining for laminin from the gastrocnemius. Scale bars = 50 μm. (**D**) The cross-sectional area of each myofiber from **B** (control, *n* = 4; skm-HIF1α, *n* = 4). (**E**) mRNA expression levels of muscle atrophic genes (control, *n* = 5; skm-HIF1α, *n* = 7). Data are shown as the mean ± SEM. Unpaired 2-tailed Student’s *t* test (**B**, **D**, and **E**) was used for statistical analyses. *P < 0.05, **P < 0.01, and ***P < 0.001.

**Figure 7:**
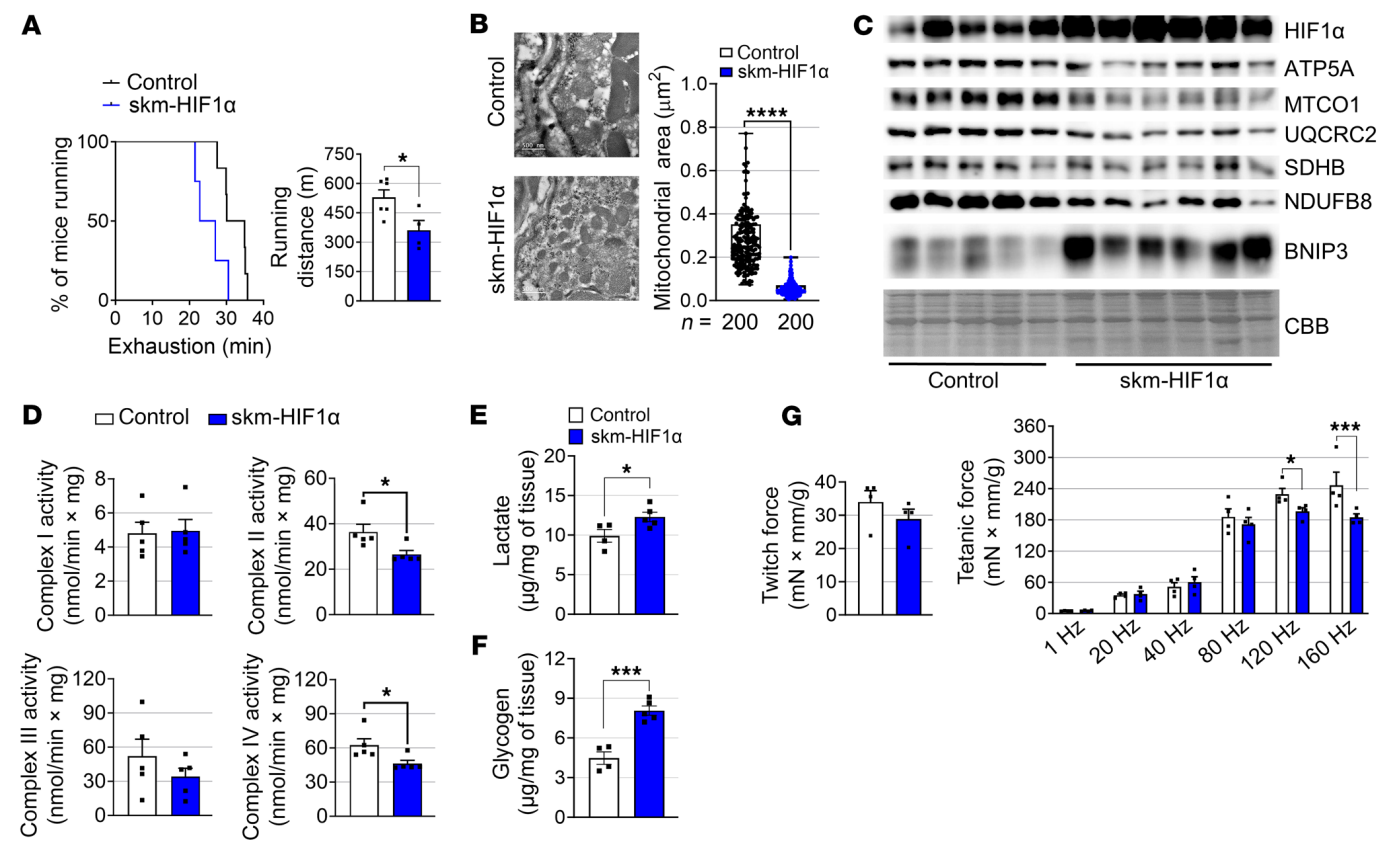
HIF1α in skeletal muscle impairs exercise performance. (**A**) Percentage of mice running before exhaustion (left) and running distance (right) (control, *n* = 6; skm-HIF1α, *n* = 4). (**B**) Transmission electron microscopy images (left) and size of the mitochondria (200 mitochondria were analyzed; right). Scale bar = 500 μm. (**C**) Immunoblot analysis of the soleus (control, *n* = 5; skm-HIF1α, *n* = 6). (**D**) Enzymatic activities of the mitochondrial complexes I, II, III, and IV (control, *n* = 5; skm-HIF1α, *n* = 5). (**E**) Intramuscular lactate content in the gastrocnemius (control, *n* = 4; skm-HIF1α, *n* = 5). (**F**) Intramuscular glycogen content in the gastrocnemius (control, *n* = 4; skm-HIF1α, *n* = 5). (**G**) Twitch force and tetanic plantar flexion torque normalized to body weight (control, *n* = 4; skm-HIF1α, *n* = 4). Data are shown as the mean ± SEM. Unpaired 2-tailed Student’s *t* test (**A**, **B**, and **D**–**G**) or 2-way ANOVA with Bonferroni’s post hoc test (**G**) was used for statistical analyses. *P < 0.05, ***P < 0.001, and ****P < 0.0001. CBB, Coomassie blue stain.

As oxidative fibers were increased in PHD mTKO mice, we analyzed the proportion of oxidative and glycolytic myofibers in skm-HIF1α mice. The skm-HIF1α mice exhibited increased oxidative fibers with more reddish muscles compared with control groups (Figure 6, A and B). In line with this, the gastrocnemius in the skm-HIF1α mice showed a significant increase in oxidative MYH2 and MYH7 protein levels (Supplemental Figure 9A). The mRNA expression levels of oxidative fiber–specific markers were also elevated in the skeletal muscles of skm-HIF1α mice (Supplemental Figure 9B). This increase in the number of oxidative fibers was accompanied by a reduction in the overall diameter of type I and IIa myofibers compared with control mice without the expression of muscle atrophy–related E3 ligase genes (*Fbxo32* and *Trim63*) being upregulated (Figure 6, C–E). Despite an increased oxidative fiber ratio, similar to PHD mTKO mice, skm-HIF1α mice exhibited impaired exercise performance and had abnormally small mitochondria (Figure 7, A and B). Additionally, the expression of proteins associated with OXPHOS decreased, while the mitochondrial fragmentation marker BNIP3 increased (Figure 7C and Supplemental Figure 9C). Reduced expression of transcripts involved in the mitochondrial oxidative function and biogenesis was also observed in the skm-HIF1α mice (Supplemental Figure 9, D–F). Moreover, enzymatic activities of mitochondrial complexes were significantly lower in the muscles of skm-HIF1α mice (Figure 7D), accompanied by increased intramuscular lactate and glycogen content, as well as reduced tetanic force, similar to the phenotype observed in PHD mTKO mice (Figure 7, E–G). These results indicate that the increases in oxidative fibers, together with the paradoxical inhibition of mitochondrial OXPHOS observed in PHD mTKO mice, are largely attributable to the stabilization of HIF1α.

To examine whether mitochondrial OXPHOS is impaired in a cell-autonomous manner, differentiated C2C12 myotubes were cotransduced with AAV-HSA-Cre and either AAV-DIO-GFP or AAV-DIO-HIF1α (Supplemental Figure 10A). In this system, more than 50% of the myotubes expressed GFP when transduced with AAV-DIO-GFP, and AAV-DIO-HIF1α transduction resulted in robust induction of HIF1α target genes (Supplemental Figure 10, B and C). In cells transduced with HIF1α, reduced expression of mitochondrial biogenesis–related genes, diminished OXPHOS levels, and increased BNIP3 protein expression were observed, collectively indicating that HIF1α impairs mitochondrial OXPHOS in a cell-autonomous manner (Supplemental Figure 10, D and E).

### HIF2α in skeletal muscle mitigates weight gain and improves glucose tolerance.

**Figure 8:**
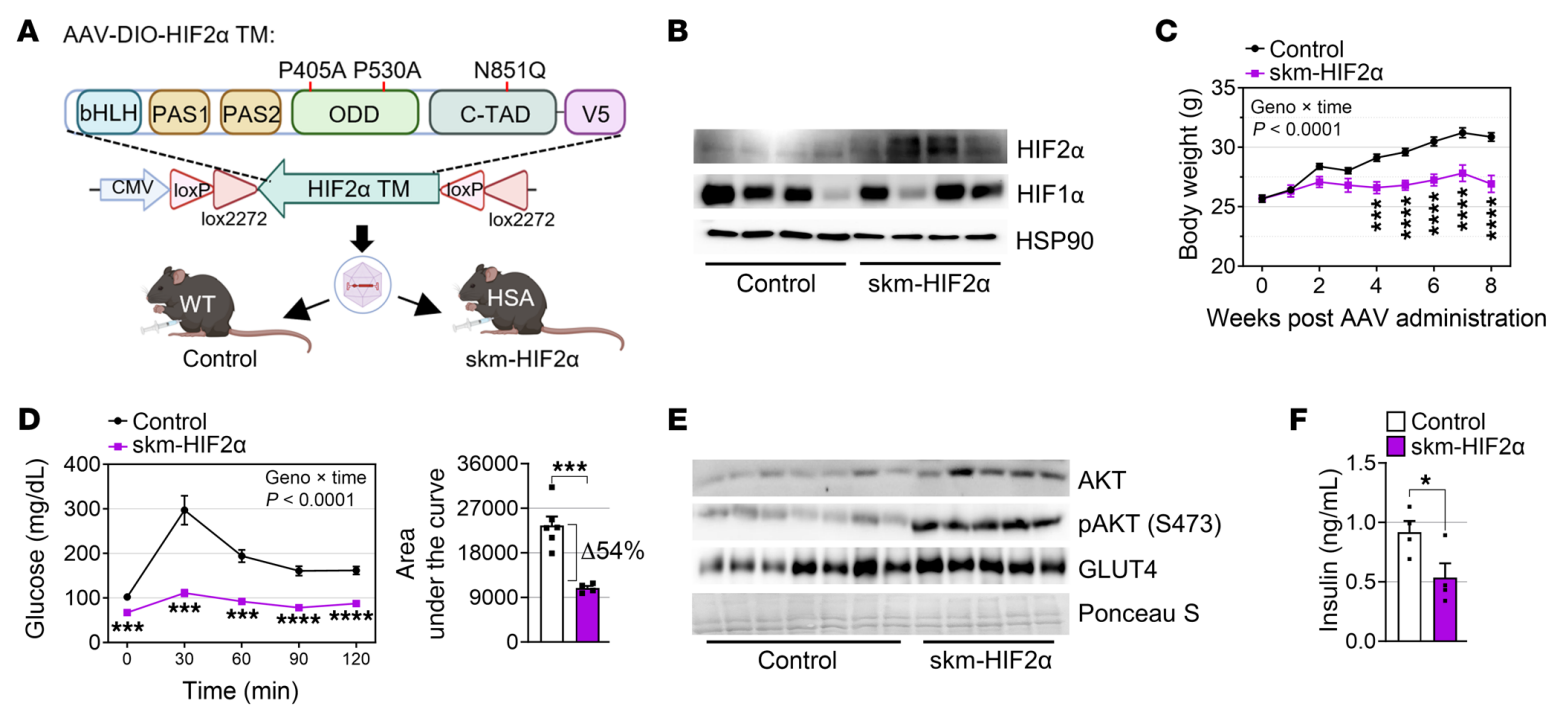
HIF2α stabilization improves glucose tolerance and reduces body weight. (**A**) Schematic of the myofiber-specific transduction of HIF2α-TM and generation of skm-HIF2α mice. (**B**) Immunoblot analysis of the soleus confirmed the transduction of HIF1α (control, *n* = 4; skm-HIF2α, *n* = 4). (**C**) Body weights of mice on an NCD (control, *n* = 6; skm-HIF2α, *n* = 5). (**D**) GTT on 14-week-old mice administered an NCD (left) and area under the curve of glucose levels (right) (control, *n* = 6; skm-HIF2α, *n* = 5). (**E**) Representative immunoblots from the gastrocnemius (control, *n* = 7; skm-HIF2α, *n* = 5). (**F**) Fasting serum insulin levels in mice (control, *n* = 4; skm-HIF2α, *n* = 4). Data are shown as the mean ± SEM. Unpaired 2-tailed Student’s *t* test (**D** and **F**) or 2-way ANOVA with Bonferroni’s post hoc test (**C** and **D**) was used for statistical analyses. *P < 0.05, ***P < 0.001, and ****P < 0.0001.

Next, we generated myofiber-specific, inducible HIF2α–overexpressing mice carrying a TM (HIF2α P405A/P530A/N851Q) to achieve stable expression under normoxic conditions and found that HIF2α expression levels were comparable with those observed in PHD mTKO mice (Figure 8, A and B, and Supplemental Figure 11, A and B). Using these mice, we examined the effects of HIF2α transduction on skeletal muscle and found that both male and female skm-HIF2α mice gained significantly less body weight than control mice while being fed an NCD (Figure 8C and Supplemental Figure 11C). Moreover, we found that the weights of both muscles and adipose tissues were decreased, suggesting that the reduced weight gain in skm-HIF2α mice is due to a decrease in both fat mass and lean mass (Supplemental Figure 11D). Furthermore, glucose tolerance was dramatically enhanced in skm-HIF2α mice (Figure 8D). Notably, this enhancement was substantially greater than that observed in skm-HIF1α mice and was comparable with the level observed in PHD mTKO mice (Figure 1F, Figure 4D, and Figure 8D). Elevated GLUT4 expression, AKT phosphorylation (Figure 8E and Supplemental Figure 11E), and decreased serum insulin levels were also observed in skm-HIF2α mice (Figure 8F), suggesting enhanced glucose uptake and insulin signaling. In addition, gene expression related to fatty acid uptake and lipid storage, as well as intramuscular and serum FFA levels, were only marginally affected in skm-HIF2α mice (Supplemental Figure 11, F–H). These findings suggest that improved glucose tolerance observed in PHD mTKO mice is primarily driven by HIF2α rather than HIF1α.

### HIF2α in skeletal muscle has a negligible effect on oxidative fiber ratio and mitochondrial oxidative phosphorylation.

**Figure 9:**
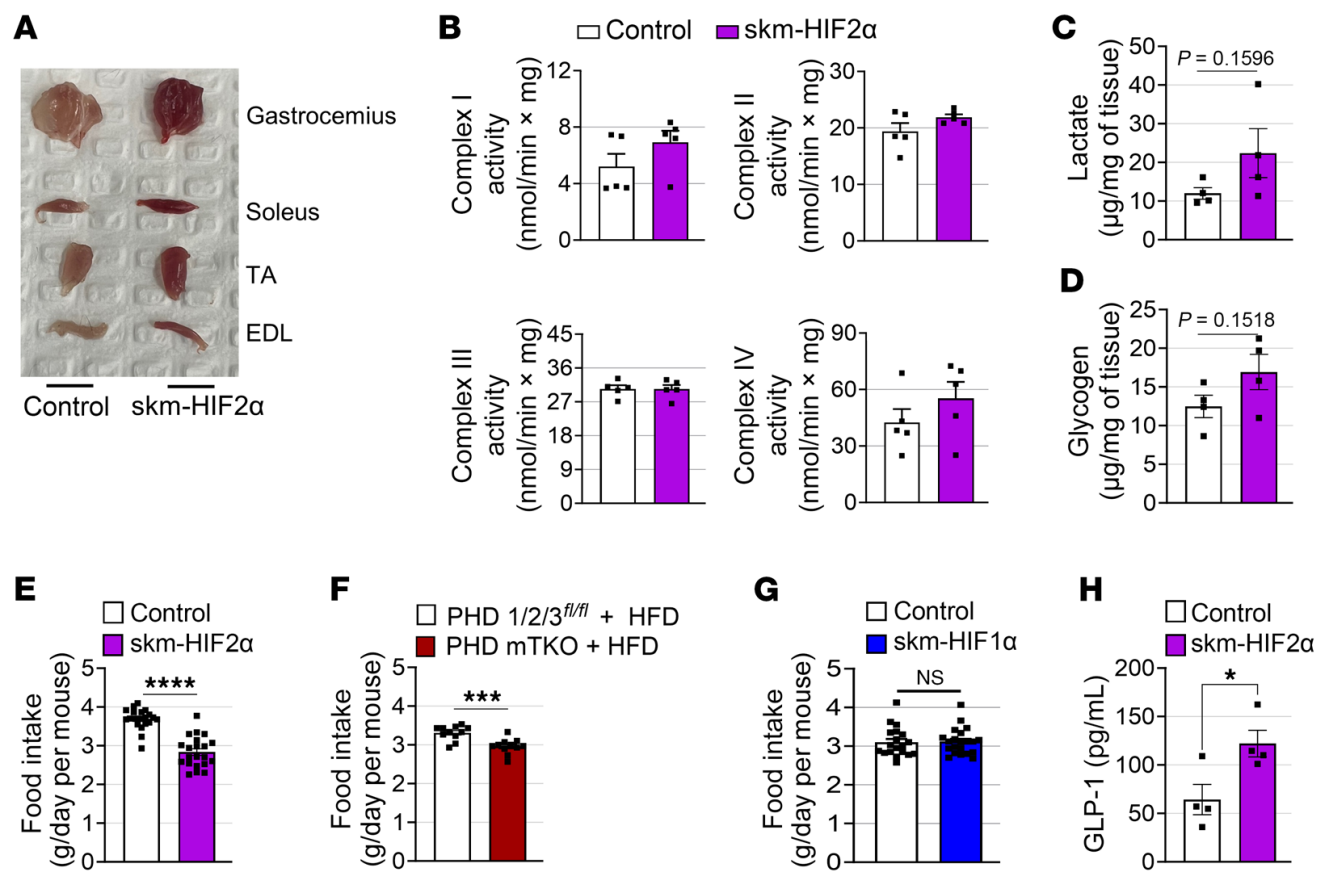
HIF2α stabilization exerts limited effects on mitochondrial function and reduces food intake. (**A**) Skeletal muscles isolated from a 5-month-old skm-HIF2α mouse and a control mouse. (**B**) Enzymatic activities of the mitochondrial complexes I, II, III, and IV (control, *n* = 5; skm-HIF2α, *n* = 5). (**C**) Intramuscular lactate content in the gastrocnemius (control, *n* = 4; skm-HIF2α, *n* = 4). (**D**) Intramuscular glycogen content in the gastrocnemius (control, *n* = 4; skm-HIF2α, *n* = 4). (**E**–**G**) Food intake of skm-HIF2α mice on an NCD (**E**), PHD mTKO mice on an HFD (**F**), and skm-HIF1α mice on an NCD (**G**). (**H**) Serum GLP-1 level in skm-HIF2α mice (control, *n* = 4; skm-HIF2α, *n* = 4). Data are shown as the mean ± SEM. Unpaired 2-tailed Student’s *t* test was used for statistical analyses (**B**–**H**). *P < 0.05, ***P < 0.001, and ****P < 0.0001.

Although skm-HIF2α mice exhibited markedly redder muscles compared with control mice (Figure 9A), the proportions of oxidative and glycolytic fibers were unchanged, and the expression levels of their corresponding marker genes also remained unaltered (Supplemental Figure 11, I and J). The expression of *Fbxo32* and *Trim63* also was not increased in skm-HIF2α mice (Supplemental Figure 11K).

We next evaluated mitochondrial OXPHOS in the skeletal muscle of skm-HIF2α mice. The expression levels of OXPHOS components and BNIP3 were unchanged in skm-HIF2α mice (Supplemental Figure 12A), and mitochondrial complex activities were also unaffected (Figure 9B). Consistently, in differentiated C2C12 myotubes overexpressing HIF2α, generated by transducing AAV-HSA-Cre and AAV-DIO-HIF2α, OXPHOS expression remained unaltered (Supplemental Figure 12B). Moreover, unlike PHD mTKO and skm-HIF1α mice, skm-HIF2α mice showed no changes in muscle twitch or tetanic force (Supplemental Figure 12C). These findings indicate that HIF2α does not impact the oxidative–glycolytic fiber transition nor alter mitochondrial OXPHOS in skeletal muscle.

Notably, although skeletal muscle glucose uptake was expected to increase, mitochondrial activity remained unchanged, and intramuscular FFA concentrations, lactate production, and glycogen content were all unaltered (Figure 9, B–D), despite reductions in body weight and improved glucose tolerance. Because brown adipose tissue can regulate systemic metabolism through uncoupling protein 1–mediated (UCP1-mediated) nonshivering thermogenesis (30), we next examined UCP1 expression in brown adipose tissue; however, it remained unchanged in skm-HIF2α mice (Supplemental Figure 12D). A closer examination revealed a significant reduction in food intake in skm-HIF2α mice (Figure 9E). A similar decrease in food intake was observed in HFD-fed PHD mTKO mice, which showed profound weight loss (Figure 9F), whereas skm-HIF1α mice displayed neither a decrease in food intake nor a reduction in body weight (Figure 9G) and showed only modest improvements in glucose tolerance. Interestingly, circulating glucagon-like peptide-1 (GLP-1) levels were increased nearly 2-fold in skm-HIF2α mice (Figure 9H). These findings suggest that stabilization of HIF2α in skeletal muscle may reduce food intake and improve glucose tolerance, at least in part through elevated GLP-1 expression.

### HIF2α in skeletal muscle induces polycythemia and increases mortality.

**Figure 10:**
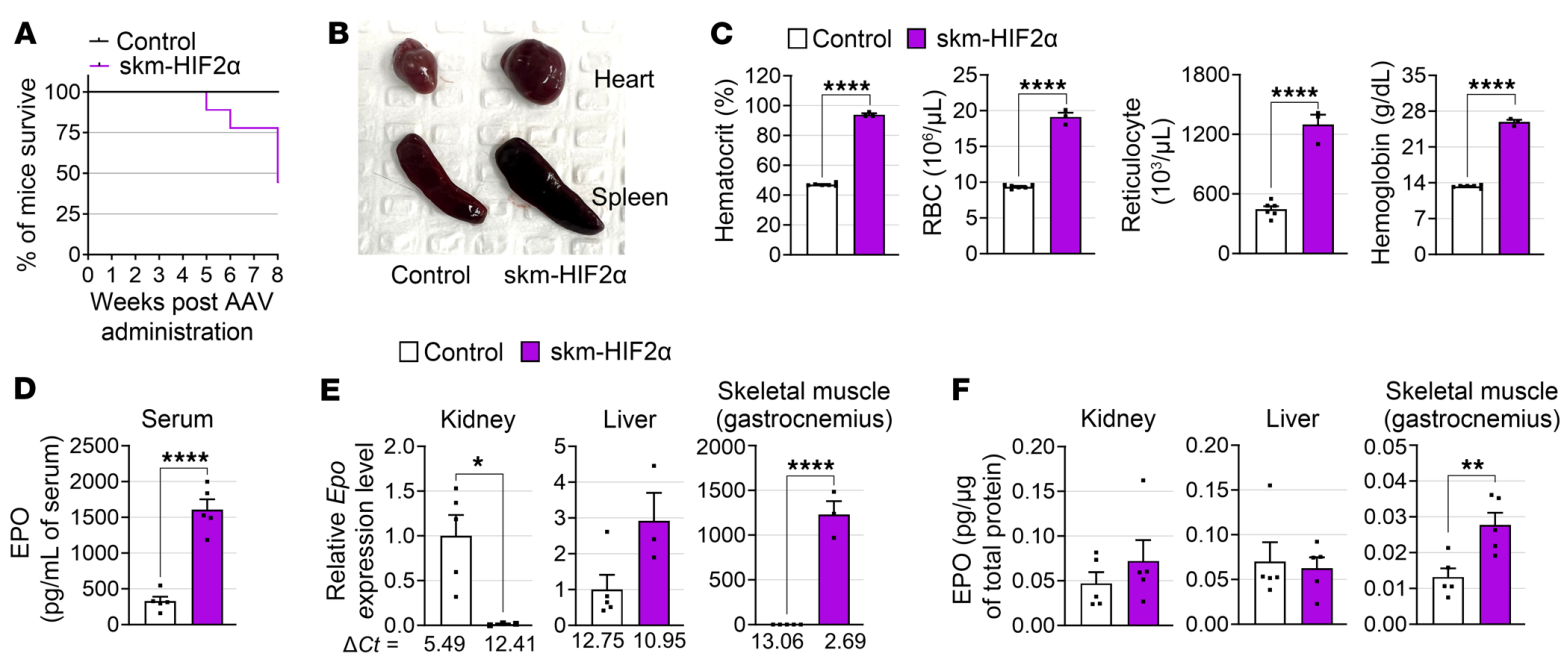
HIF2α in skeletal muscle induces polycythemia. (**A**) Survival ratio of skm-HIF2α male mice (control, *n* = 14; skm-HIF2α, *n* = 9). (**B**) Representative image of ballooned heart and spleen of skm-HIF2α mice. (**C**) Hematologic parameters of skm-HIF2α mice (control, *n* = 6; skm-HIF2α, *n* = 3). (**D**) EPO levels in serum (control, *n* = 5; skm-HIF2α, *n* = 5). (**E**) *Epo* mRNA expression levels in indicated tissues (control, *n* = 6; skm-HIF2α, *n* = 3). (**F**) EPO protein levels in indicated tissues (control, *n* = 5; skm-HIF2α, *n* = 5). Data are shown as the mean ± SEM. Unpaired 2-tailed Student’s *t* test was used for statistical analyses (**C**–**F**). *P < 0.05, **P < 0.01, and ****P < 0.0001.

Surprisingly, we noticed that skm-HIF2α mice exhibited increased mortality even as early as 5 weeks after AAV administration (Figure 10A). Furthermore, we observed abnormally large spleens and hearts in skm-HIF2α mice (Figure 10B), indicating potential alterations in hematopoietic and cardiovascular activity. Indeed, skm-HIF2α mice showed supraphysiologically increased hematocrit (> 90%) and hemoglobin levels, alongside RBC and reticulocyte counts, strongly indicating polycythemia (Figure 10C). Additionally, we found that the serum EPO level, which primarily stimulates erythropoiesis, was remarkably increased in skm-HIF2α mice (Figure 10D). It is widely believed that EPO expression is strongly restricted to the kidney and liver; however, neither the *Epo* gene nor EPO protein expression was elevated in the kidney or liver of skm-HIF2α mice (Figure 10, E and F). Strikingly, we observed a markedly upregulated expression of *Epo* transcripts and EPO protein in skeletal muscle (gastrocnemius) (Figure 10, E and F). Importantly, we also observed increased *Epo* mRNA levels in differentiated C2C12 myotubes overexpressing HIF2α, accompanied by elevated EPO concentrations in the culture media (Supplemental Figure 13, A and B). This finding demonstrates that skeletal muscle can serve as an alternative site for EPO production and secretion, contributing to the induction of erythropoiesis. Despite the abnormally elevated hematocrit, twitch and tetanic force in the skeletal muscle of skm-HIF2α mice remained unchanged (Supplemental Figure 12C). Moreover, in the open-field test, ambulatory distance, center distance, and margin distance were comparable across control and skm-HIF2a mice, demonstrating that spontaneous locomotor activity remained unchanged (Supplemental Figure 13C). However, both rectal and surface temperatures were lower than in controls (Supplemental Figure 13, D and E). These findings suggest that markedly increased hematocrit led to higher blood viscosity and impaired circulation, which placed additional workload on the heart and resulted in cardiac hypertrophy and hypothermia, ultimately increasing mortality.

We also observed significantly increased hematocrit levels in PHD mTKO mice (Supplemental Figure 14, A–C). Moreover, the expression of *Epo* transcripts was markedly upregulated in the skeletal muscle of PHD mTKO mice, without any corresponding increase in *Epo* levels in the kidney or liver (Supplemental Figure 14E). However, in the PHD mKO mice, *Epo* mRNA expression was not increased (Supplemental Figure 14F). The increase in hematocrit was also observed in female PHD mTKO mice, although the magnitude was lower than in males (~95% in males vs. ~75% in females) (Supplemental Figure 14G). This difference subsequently led to an increased mortality rate observed only in male mice (Supplemental Figure 14, D and H). In contrast, skm-HIF1α mice did not exhibit increased mortality or hematocrit levels (Supplemental Figure 14, I–K).

### The PHD1–HIF2α axis increases EPO production in skeletal muscle.

**Figure 11:**
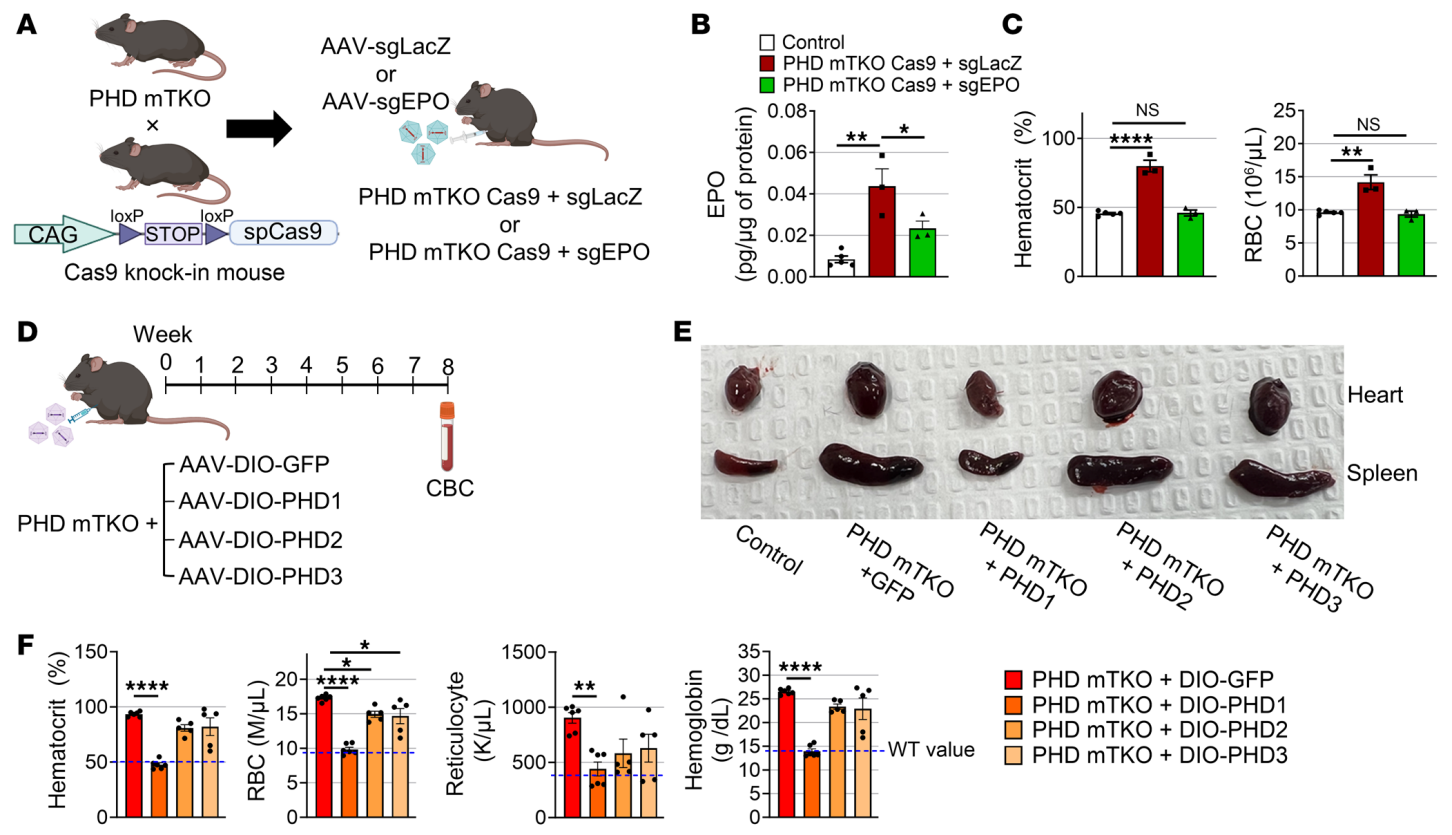
The PHD1–HIF2α axis increases EPO production in skeletal muscle. (**A**) Schematic illustrating the generation of the myofiber-specific *Epo* knockout in PHD mTKO mice. PHD mTKO mice were crossed with LSL-Cas9 knock-in mice to obtain PHD mTKO Cas9 animals. MyoAAV2A-sgEPO or sgLacZ was administered intraperitoneally to PHD mTKO Cas9 mice. *Phd1^fl/fl^Phd2^fl/fl^Phd3^fl/fl^-*Cas9 mice lacking HSA-Cre and injected with MyoAAV2A-sgLacZ served as controls. (**B**) EPO protein levels in gastrocnemius (control, *n* = 5; PHD mTKO Cas9 + sgLacZ, *n* = 3; PHD mTKO Cas9 + sgEPO, *n* = 3). (**C**) Hematologic parameters of mice (control, *n* = 5; PHD mTKO Cas9 + sgLacZ, *n* = 3; PHD mTKO Cas9 + sgEPO, *n* = 3). (**D**) Schematic illustrating the generation of indicated mice. (**E**) Representative image of heart and spleen of indicated mice. (**F**) Hematologic parameters of mice (PHD mTKO + DIO-GFP, *n* = 6; PHD mTKO + DIO-PHD1, *n* = 6; PHD mTKO + DIO-PHD2, *n* = 5; PHD mTKO + DIO-PHD3, *n* = 5). Data are shown as the mean ± SEM. One-way ANOVA with Bonferroni’s post hoc test was used for statistical analyses (**B**, **C**, and **F**). *P < 0.05, **P < 0.01, and ****P < 0.0001.

To further confirm that the increased hematocrit observed in skm-HIF2α and PHD mTKO mice was driven by muscle-derived EPO, we generated PHD mTKO mice that additionally express Cas9 specifically in skeletal muscle by crossing *Phd1^fl/fl^*, *Phd2^fl/fl^*, and *Phd3^fl/fl^* HSA-Cre mice with *Rosa26*-LSL-Cas9 mice (Figure 11A). Administration of an AAV-sgRNA targeting *Epo* (sgEPO) to these mice significantly reduced the elevated intramuscular EPO levels induced by PHD deficiency (Figure 11B). Moreover, muscle-specific EPO deletion normalized the increased hematocrit, RBC and reticulocyte counts, and hemoglobin levels observed in PHD mTKO mice (Figure 11C and Supplemental Figure 14L). In contrast, the reductions in body weight and improvements in glucose tolerance caused by PHD deficiency were only minimally affected by EPO knockout (Supplemental Figure 14, M and N). These findings indicate that HIF2α-dependent EPO production in skeletal muscle promotes erythropoiesis and is responsible for the development of polycythemia.

Next, to determine which PHD isoform plays a critical role in HIF2α-mediated EPO production, we reintroduced each PHD isoform individually into the skeletal muscle of PHD mTKO mice using the AAV-DIO system (Figure 11D). Remarkably, expression of PHD2 or PHD3 had no effect, whereas expression of PHD1 alone restored the enlarged heart and spleen observed in PHD mTKO mice to normal size (Figure 11E). Furthermore, the elevated hematocrit, RBC and reticulocyte counts, and hemoglobin levels in PHD mTKO mice were reduced to near-normal levels upon muscle-specific expression of PHD1 (Figure 11F). These findings indicate that the PHD1–HIF2α axis in skeletal muscle regulates muscle-derived EPO production.

Together, our findings suggest that skeletal muscle, in addition to the kidney and liver, has the capacity to produce EPO. To determine whether muscle contributes to EPO production under physiological conditions, we established a phlebotomy model by removing approximately 500 μL of blood through retro-orbital bleeding in normal mice. As expected, this model exhibited reduced hematocrit (Supplemental Figure 15A), which was accompanied by increased HIF2α stabilization in skeletal muscle (Supplemental Figure 15B). However, *Epo* mRNA expression in muscle was not elevated (Supplemental Figure 15C). We next generated skeletal muscle–specific HIF2α-knockout mice by crossing *Hif2a^fl/fl^* with HSA-Cre mice (Supplemental Figure 15D). These mice displayed hematocrit levels comparable with controls and showed no change in muscle *Epo* mRNA expression (Supplemental Figure 15, E and F). When subjected to phlebotomy, skeletal muscle–specific HIF2α-knockout mice exhibited serum EPO levels comparable with those of control mice (Supplemental Figure 15G). These observations indicate that HIF2α-dependent EPO induction does not occur in skeletal muscle under physiological conditions, although it is possible that muscle-derived EPO could be engaged under certain pathological circumstances, such as states in which renal EPO production is compromised.

## Discussion

This study investigated the comprehensive roles of HIF1α or HIF2α in glucose tolerance in skeletal muscle, resistance against weight gain, myofiber composition, and mitochondrial oxidative respiration. These findings were achieved in mice through myofiber-specific PHD depletion (PHD mTKO) or myofiber-specific overexpression of normoxia-stable HIFα (skm-HIF1α and skm-HIF2α). Initial experiments using single-PHD knockout mice revealed that depletion of a single PHD isoform is insufficient to stabilize HIFα and to induce its target genes in skeletal muscle, suggesting a potential compensatory mechanism among the PHD isoforms (30). Therefore, we generated PHD mTKO mice and evaluated HIFα stabilization. HIF1α is known to be ubiquitously expressed in most cell types, whereas HIF2α expression is reported to be more restricted in specific cell types (31). Using PHD mTKO mice, we discovered that both HIF1α and HIF2α are expressed in skeletal muscle cells (Figure 1B). Building on this finding further, we generated HIF1α- or HIF2α-transduced mice and explored their phenotypes in more depth. Another potential option to stabilize HIFα expression could be to knock out VHL expression since VHL is an E3 ligase for HIFα. However, given the presence of other VHL substrates and the role of VHL as a tumor suppressor, VHL-knockout mice would not be an appropriate model for investigating the role of HIFα (32, 33).

One of the most striking findings of our study is that skeletal muscle HIF2α promotes EPO production within the muscle itself, ultimately leading to polycythemia. EPO has been thought to be produced exclusively by the kidney and liver (34, 35), where EPO production is triggered by PHD deficiency or HIF2α stabilization (36–39). However, our findings provide novel insights demonstrating that skeletal muscle can also serve as a source of EPO secretion and contribute to erythropoiesis in adult mammals. Interestingly, our results showed that hematocrit levels reached supraphysiological values comparable with or even higher than those observed in similar animal models where PHD deletion was applied to renal cells or hepatocytes (37, 39). This is likely because skeletal muscle is the largest organ, accounting for approximately 50% of the total body mass, which may result in a higher overall level of EPO production. Furthermore, PHD mTKO mice exhibited sex-dependent differences in hematocrit levels, with females displaying lower hematocrit levels than males. This discrepancy is likely due to the inherently lower muscle mass in females than males, leading to reduced EPO production in skeletal muscle. Interestingly, skeletal muscle–specific knockout of PHD1 did not significantly affect HIF2α stability or alter *Epo* mRNA expression in muscle, likely because other PHD isoforms compensated by catalyzing HIF2α hydroxylation. However, reexpressing PHD1 in PHD mTKO mice restored hematocrit levels to normal, indicating that PHD1 alone is sufficient to drive complete degradation of HIF2α. These findings highlight that, at least in skeletal muscle, the PHD1–HIF2α axis plays a critical regulatory role.

We also found that both skeletal muscle HIF1α and HIF2α contribute to improved glucose tolerance, with HIF2α playing a predominant role in this effect. Notably, skeletal muscle HIF1α did not confer resistance to obesity or reduce serum insulin levels, suggesting that HIF2α plays a distinct role in regulating body mass and insulin sensitivity. However, the precise mechanism through which HIF2α induces weight loss has yet to be elucidated. We demonstrated that the weight loss observed in skm-HIF2α mice results from reduced fat and muscle mass. One possible explanation is that a drastic increase in circulating EPO concentrations driven by HIF2α contributes to reduced fat mass (40), which might lead to reduced exercise performance and muscular degeneration (41). Previous research also demonstrated that the interaction between EPO and the EPO receptor expressed in nonerythroid cells contributes to resistance against glucose intolerance and weight gain, suggesting a potential mechanism in skm-HIF2α mice (42). However, using a direct animal model in which EPO was deleted specifically in skeletal muscle of PHD mTKO mice, our results demonstrated that muscle-derived EPO affected erythropoiesis but had no impact on body weight and only minimal effects on glucose tolerance. This discrepancy may arise from differences between exogenously administered recombinant EPO and endogenously regulated, tissue-derived EPO.

An additional noteworthy observation is that the reduction in muscle mass observed in skm-HIF2α mice was not evident in PHD mTKO mice. Given that PHD deficiencies constitutively stabilize HIFα from the embryonic stage in PHD mTKO mice, various compensatory mechanisms might have occurred to prevent muscular atrophy. Previous studies have reported that administering recombinant EPO led to weight loss in male mice but not females (43). In our study, male and female PHD mTKO and skm-HIF2α mice exhibited weight loss, suggesting that factors beyond EPO, potentially additional effects of skeletal muscle HIF2α, may contribute to the observed metabolic changes. One possible hypothesis is that the reduced food intake and improved glucose tolerance observed in skm-HIF2α mice are mediated by increased GLP-1 signaling, as serum GLP-1 levels were elevated in these mice.

Another notable finding in our study is that, despite an increased proportion of oxidative fibers, mice with stabilized HIF1α exhibited impaired mitochondrial respiration. Consistent with our results, previous reports have shown that hypoxia can increase the number of oxidative fibers with impaired mitochondrial respiration through HIF1α in cultured C2C12 cells (44). While the effect of HIF1α deficiency on exercise performance has been previously reported (45), the impact of constitutive HIF1α stabilization on exercise capacity remained unknown in vivo. Using our novel animal models, we demonstrated that HIF1α stabilization in skeletal muscle directly impairs exercise capacity and muscle tetanic force. Interestingly, the increased proportion of oxidative fibers was accompanied by a reduction in the size of oxidative fibers. One possible explanation is that, under physiological conditions, PHD1 and PHD2 are more abundantly expressed in oxidative fiber–rich muscles such as the soleus than in glycolytic muscles such as the extensor digitorum longus (Supplemental Figure 1A). That is, oxidative fibers normally contain higher levels of PHD1 and PHD2, which actively degrade HIF1α; thus, genetic ablation of these PHDs may disproportionately increase HIF1α stability within oxidative fibers, ultimately leading to their reduced fiber size. Alternatively, the increased proportion of oxidative fibers, together with their smaller size and the absence of upregulation in atrophy-related genes, may reflect enhanced proliferation and differentiation of muscle stem cells (MuSCs). However, because our experimental models involve genetic manipulation exclusively within myofibers, it remains possible that secondary, myofiber-derived factors indirectly influence MuSC proliferation and differentiation. Therefore, additional studies will be required to establish the mechanistic link between HIF1α stabilization in myofibers and MuSC differentiation.

Our study has several limitations. First, except for the PHD mTKO cohort, body composition was not assessed by dual-energy x-ray absorptiometry. Instead, adiposity was primarily estimated by wet weights of dissected fat depots, which provides a reliable but less comprehensive assessment of whole-body composition. In addition, in HFD-fed PHD mTKO mice, lean mass was unchanged; however, the glucose dose during the glucose tolerance test (GTT) was normalized to total body weight rather than lean mass, which may have affected the magnitude of the observed difference in glucose tolerance. Second, although increased circulating GLP-1 levels were observed in skm-HIF2α mice, the mechanistic link between skeletal muscle HIF2α stabilization and enhanced GLP-1 secretion from intestinal L-cells remains unclear. Further studies will be required to identify the muscle-derived signal and the interorgan communication pathway responsible for this effect. Third, despite marked reductions in body weight and food intake in skm-HIF2α mice, indirect calorimetry was not performed. Therefore, it cannot be determined whether the reduction in body weight was driven solely by decreased energy intake or whether changes in energy expenditure and/or substrate utilization also contributed.

In the present study, we unveiled the multifaceted functions of HIFα in muscle cells. A pharmacological PHD inhibitor is an emerging strategy for treating anemia in patients with chronic kidney disease (46, 47), as a PHD inhibitor promotes HIFα stabilization and EPO production in the kidney. However, as PHD inhibitors exert systemic effects and influence a myriad of genes through HIFα stabilization, the pleiotropic effects of PHD inhibition may play a significant role beyond EPO regulation in the kidney, potentially impacting various physiological processes (5, 48). Based on our study, using a PHD inhibitor should be carefully considered to prevent muscular dysfunctions, such as impaired mitochondrial oxidative respiration. In addition, our findings may be relevant for patients with chromic kidney disease who present with compromised liver function, offering potential insights into new strategies for managing anemia.

## Methods

### Sex as a biological variable.

Our study examined male and female mice, and similar findings are reported for both sexes.

### Mice.

The mice in this study were in a C57BL/6J background and were housed under a 12 h light/12 h dark cycle with ad libitum access to food and water at 22°C (room temperature) and 46%–50% relative humidity. HSA-Cre mice (strain 006149), Rosa26-LSL-Cas9 mice [B6J.129(B6N)-Gt(ROSA)26Sor^tm1(CAG-cas9*,-EGFP)Fezh^/J, strain 026175], HIF2α-floxed mice (*Epas1^tm1Mcs^*/J, strain 008407), and PHD1, PHD2, and PHD3 (PHD1/2/3) triple-floxed mice (*Egln2^tm2Fong^ Egln1^tm2Fong^* *Egln3^tm2Fong^*/J, strain 028097) were purchased from The Jackson Laboratory. PHD1 mKO, PHD2 mKO, PHD3 mKO, and PHD mTKO mice were generated by crossing HSA-Cre mice with the PHD1/2/3 triple-floxed mice. PHD mTKO Cas9 mice were generated by crossing HSA-Cre mice with Rosa26-LSL-Cas9 mice and PHD1/2/3 triple-floxed mice. HIF2α mKO mice were generated by crossing HSA-Cre mice with HIF2α floxed mice. For the diet-induced obesity model, mice were fed an HFD of 60 kcal% fat (D12492; Research Diets). For the GTT, mice were fasted for 16 h and then injected intraperitoneally with filtered glucose solution (2 g/kg body weight). Venous blood samples were collected from punched mouse tails, and blood glucose levels were measured using Accu-Chek (Performa). Body composition parameters were assessed using dual-energy X-ray absorptiometry with an InAlyzer instrument (Medikors) located at the Korea Research Institute of Bioscience and Biotechnology (Jeongeup, South Korea). Rectal temperatures were measured using the Therma Waterproof Type T Thermometer (232-107; ThermoWorks), and infrared thermographic imaging was performed with an infrared camera (FLIR E5 wifi; Teledyne FLIR). For phlebotomy experiments, blood (500 μL) was withdrawn from the retro-orbital sinus under isoflurane anesthesia, followed immediately by intraperitoneal administration of warmed saline (500 μL) as volume replacement.

### AAV preparation and purification.

As described in our previous work, AAV harboring the target gene was produced and administered to mice (49). The AAV-DIO-HIF1α TM and AAV-DIO-HIF2α TM plasmids, which encode normoxia-stable active murine HIF1α (50) or HIF2α, were synthesized by VectorBuilder. AAV-DIO-HIF1α TM, pAAV2/9 (112865; Addgene), and the helper plasmid pAdDelta6 (112864; Addgene) were cotransfected into HEK 293T cells at a 1:1:1 molar ratio using polyetherimide. The media were refreshed 16 h after transfection, and the cells containing viral particles were harvested 60 h after transfection and lysed in AAV lysis buffer (150 mM NaCl, 20 mM Tris at pH 8.0), followed by incubation with Benzonase (E8263; Sigma-Aldrich). An iodixanol gradient was used for virus purification, followed by high-speed centrifugation and concentration using an Amicon Ultra-15 Centrifugal Filter Unit (UFC910096; Sigma-Aldrich). For AAV titration, after incubation with DNase and proteinase K to remove any residual plasmid DNA and proteins, the number of viral particles was quantified using a CFX96 Real-Time PCR system (Bio-Rad). AAV2/9-DIO-HIF1α TM was administered to mice intraperitoneally and intramuscularly, delivering 2 × 10^12^ viral particles intraperitoneally and 3 × 10^12^ intramuscularly per mouse. AAV-DIO-HIF2α TM, AAV-DIO-PHD1, AAV-DIO-PHD2, AAV-DIO-PHD3, and AAV-DIO-GFP were packaged with pMyoAAV2A (224440; Addgene) and pAdDelta6 in identical manners, which show enhanced myotropism (51). In total, 1 × 10^12^ viral particles of AAV-DIO-HIF2α TM, AAV-DIO-PHD1, AAV-DIO-PHD2, AAV-DIO-PHD3, or AAV-DIO-GFP were intraperitoneally injected into each mouse. For CRISPR/Cas9-mediated *Epo*-knockout experiments, *Epo*-specific gRNAs were designed in silico using the CRISPick sgRNA design tool (Broad Institute). gRNAs with the fewest predicted off-target mismatches were selected, synthesized by Bioneer, and subsequently inserted into a pAAV-U6-sgRNA-CMV-mCherry vector that was generated by combining 2 plasmids (87916 and 85451; Addgene). AAV-sgEpo was packaged with pMyoAAV2A, and 1 × 10^12^ viral particles of AAV-sgEpo were intraperitoneally injected into each mouse. The gRNA sequences targeting *Epo* are provided in Supplemental Table 1.

### Treadmill running.

The mice were acclimatized to the treadmill (LE8710MTS; Harvard Apparatus) with a daily 10 min run at 10 cm/s for 5 days with an uphill incline of 10° before the forced running performance test. On the test day, the mice were placed on a treadmill with an uphill incline of 10° and started at an initial speed of 10 cm/s. The speed was increased by 5 cm/s every 4 min until it reached 50 cm/s. The mice were considered exhausted when their hindlimbs remained in contact with the electric grid for more than 5 s.

### Open-field test.

Locomotor activity in the open field was assessed using the TruScan Photo Beam Activity System (Coulbourn Instruments). Each mouse was placed in the center of the arena, and locomotor parameters including ambulatory, margin, and center activity were quantified by measuring time (s) and distance (cm) over a 60 min period.

### Histology.

Immediately after euthanasia, the indicated muscles were dissected and promptly frozen in cooled isopentane. The frozen muscles were then sliced into 10 μm sections using a cryostat microtome (CM1850; Leica Biosystems). For H&E staining, the sections were fixed in 10% neutral buffered formalin and subsequently stained with H&E. For immunofluorescence analysis, muscle sections were incubated with primary antibodies against MyHC1 (BA-D5; DSHB), MyHC2a (SC-71; DSHB), MyHC2b (BF-F3; DSHB), or laminin (L9393; Sigma-Aldrich) at 4°C overnight. Following incubation, sections were washed and incubated with secondary antibodies (Supplemental Table 2) at room temperature for 1 h. Images were acquired using a confocal microscope (LSM 900; Carl Zeiss). The cross-sectional area of the myofibers was quantified using ImageJ (NIH) software.

### Transmission electron microscopy and image analysis.

Immediately after euthanasia, the gastrocnemius muscles were fixed in 50 mM cacodylate buffer containing 2% paraformaldehyde and 2% glutaraldehyde at room temperature for 4 h. After washing with the same buffer, the samples were fixed in 1% OsO_4_ in 50 mM cacodylate buffer at room temperature for 1 h. Following another buffer wash, the samples were dehydrated through a series of graded ethanol solutions (30%–100%). The samples were embedded in LR White resin and incubated at 50°C for 24 h. Ultrathin sections (80–100 nm) were obtained using an ultramicrotome with a diamond knife, stained with uranyl acetate and lead citrate, and examined using a transmission electron microscope (JEM-2100F; Jeol).

### Blood and tissue chemistry.

Blood samples were obtained after the animals were euthanized. Hematologic parameters were analyzed using a complete blood cell counter analyzer (Procte Dx analyzer; IDEXX Veterinary Diagnostics) or manual centrifugation using a heparin-coated capillary tube. The EPO level was measured in serum and tissues, including kidney, liver, and soleus, utilizing a mouse EPO ELISA kit (MEP00B; R&D Systems) according to the manufacturer’s instructions. FFA levels in serum and gastrocnemius muscle were measured using a Free Fatty Acid Assay Kit (ab65341; Abcam). Intramuscular glycogen and lactate content were quantified using a Glycogen Assay Kit (ab65620; Abcam) and PicoSens Lactate Assay (BM-LAC-100; BIOMAX). Serum leptin and GLP-1 levels were measured using Mouse Leptin Quantikine ELISA (MOB00B; R&D Systems) and GLP-1 ELISA (E-EL-M3108; Elabscience) kits.

### Enzymatic activity assays.

The activities of mitochondrial complexes were measured spectrophotometrically following a previously established protocol (52). Briefly, muscles were homogenized in a buffer containing 250 mM sucrose, 40 mM KCl, 20 mM Tris, 2 mM EGTA, and a protease inhibitor (10837091001; Sigma-Aldrich). The homogenates were centrifuged at 600*g* for 10 min, and the resulting supernatants were used for enzymatic assays. The specific activities of mitochondrial complexes were normalized to the protein content of each sample.

### In vivo muscle isometric force measurement.

In vivo plantar flexion torque measurements were performed using a muscle contractility apparatus (model 1300A; Aurora Scientific) as previously described (53). Mice were anesthetized with isoflurane, after which the right hind paw was fixed onto the force sensor with the ankle maintained at 90°. The hind limb was extended to place the knee in the locking position and secured at the femoral condyles. For electrical stimulation, 2 disposable monopolar electrodes were inserted subcutaneously above the tibial nerve for stimulation. Twitch force was recorded using 0.2 ms pulses, and tetanic force was recorded in response to 500 ms pulses at 10, 20, 40, 80, 120, and 160 Hz. Tetanic measurements were obtained at 1 min intervals to avoid fatigue.

### C2C12 cell culture and in vitro AAV transduction.

C2C12 myoblasts were obtained from ATCC (CRL-1772) and maintained in DMEM high glucose (OCMDMH500; OATECH) supplemented with 20% FBS (12483020; Gibco) and 1% penicillin/streptomycin (15140122; Gibco). For differentiation, the culture medium was replaced with differentiating medium consisting of DMEM high glucose supplemented with 2% horse serum (26050088; Gibco) and 1% penicillin/streptomycin when cells reached 80%–90% confluence. Differentiation medium was refreshed every 2 days. MYOAAV2A-HSA-Cre and either MYOAAV2A-DIO HIF1α TM, MYOAAV2A-DIO-HIF2α TM, or MYOAAV2A-DIO-GFP were added to C2C12 myotubes on day 4 of differentiation at a dose of 1 × 10^11^ viral particles per well. The medium was refreshed 16 h after AAV transduction, and cells were harvested on day 6 of differentiation.

### Immunoblot analysis.

The frozen tissues were homogenized in RIPA buffer (140 mM NaCl, 10 mM Tris at pH 8.0, 0.1% sodium deoxycholate, 0.1% SDS, and 1% Triton X-100) containing a protease inhibitor and phosphatase inhibitor (ab201112; Abcam). Protein concentrations were measured using DC Protein Assay Reagents (5000116; Bio-Rad). Equal amounts of protein from each sample were diluted in SDS sample buffer (60 mM Tris at pH 6.8, 25% glycerol, 0.1% bromophenol blue, 2% SDS, and 2.5% 2-mercaptoethanol). Samples were subjected to SDS-PAGE and transferred onto nitrocellulose membranes. The blots were incubated with specific antibodies against AKT (9272; Cell Signaling Technology), AKT pS473 (9271; Cell Signaling Technology), BNIP3 (A5683; ABclonal), COX4 (4850; Cell Signaling Technology), Cre-recombinase (15036; Cell Signaling Technology), DRP1 (8570; Cell Signaling Technology), GLUT4 (sc53566; Santa Cruz Biotechnology), HIF1α (NB-100-105; Novus Biologicals), HIF2α (sc13596; Santa Cruz Biotechnology), HSP90 (4874; Cell Signaling Technology), Lamin B1 (ab16048; Abcam), MYH2 (A15292; ABclonal), MYH4 (A15293; ABclonal), MYH7 (A22140; ABclonal), OXPHOS (ab110413; Abcam), OPA1 (90471; Cell Signaling Technology), PHD1 (ab113077; Abcam), PHD2 (4835; Cell Signaling Technology), PHD3 (NB100-303; Novus Biologicals), and V5 (13202; Cell Signaling Technology) according to the manufacturers’ instructions. Proteins were visualized using a luminescent image analyzer (ImageQuant LAS 4000 mini; GE Healthcare). The blot band intensity was quantified using the ImageJ program.

### Quantification of mRNA.

Total RNA was isolated from frozen tissues using TRI solution (TS200-001; BioScience Technology) according to the standard protocol. Isolated mRNA was reverse transcribed to cDNA using Moloney murine leukemia virus reverse transcriptase (28025021; Thermo Fisher Scientific) and oligo-dT. Relative gene expression was quantified using quantitative PCR on a QuantStudio 6 real-time PCR system (Thermo Fisher Scientific). The primer sequences used in this study are listed in Supplemental Table 3.

### Study approval.

The Animal Care and Use Committee of Chonnam National University (CNU IACUC-YB-2024-50, CNU IACUC-YB-2025-11, CNU IACUC-YB-2025-130, and CNU IACUC-YB-2025-136) approved all experimental protocols for the mouse studies.

### Statistics.

Data were collected from biologically independent samples; all data are expressed as the mean ± SEM. Statistical comparisons were conducted using an unpaired 2-tailed Student’s *t* test for 2-group analyses, 1-way ANOVA for multiple-group comparison, and 2-way ANOVA for analyses involving 2 independent variables, with Bonferroni’s post hoc test applied where appropriate. Statistical analyses were performed using GraphPad Prism (version 9.3.1; GraphPad Software). A *P* value less than 0.05 was considered a statistically significant difference.

### Data availability.

The data supporting the findings of this study are available in the main text or in the Supporting Data Values file.

## Author contributions

JL contributed to the writing of the original draft, visualization, validation, methodology, investigation, formal analysis, and conceptualization. MEM and EE contributed to validation and investigation. SL, YK, and SJ contributed to methodology and investigation. KK and CHC contributed to investigation. YHH, CML, TIJ, and SIP contributed to providing resources. JW contributed to resources and methodology. DK contributed to writing (review and editing), supervision, project administration, funding acquisition, methodology, data curation, and conceptualization. MJP contributed to writing (review and editing), visualization, supervision, project administration, funding acquisition, methodology, data curation, and conceptualization.

## Conflict of interest

The authors have declared that no conflict of interest exists.

## Funding support

- National Research Foundation of Korea grants (Ministry of Science and Information and Communication Technology) NRF-2021R1C1C2095021 (to MJP) and NRF-2022R1C1C1010726 (to DK).
- Technology Innovation Program (number 20026226) funded by the Ministry of Trade, Industry and Energy (Korea).

## Supplementary Material

Supplemental data

Unedited blot and gel images

Supporting data values

## Version 1 02/17/2026

In-Press Preview

## Version 2 04/15/2026

Electronic publication

## References

[1] Semenza GL (2001). HIF-1 and mechanisms of hypoxia sensing. Curr Opin Cell Biol.

[2] Semenza GL (2017). Hypoxia-inducible factors: coupling glucose metabolism and redox regulation with induction of the breast cancer stem cell phenotype. EMBO J.

[3] Taylor CT, Scholz CC (2022). The effect of HIF on metabolism and immunity. Nat Rev Nephrol.

[4] Ivan M (2017). The EGLN-HIF O_2_-sensing system: multiple inputs and feedbacks. Mol Cell.

[5] Maxwell PH, Eckardt KU (2016). HIF prolyl hydroxylase inhibitors for the treatment of renal anaemia and beyond. Nat Rev Nephrol.

[6] Kim WY, Kaelin WG (2004). Role of VHL gene mutation in human cancer. J Clin Oncol.

[7] Lundby C (2009). The response of human skeletal muscle tissue to hypoxia. Cell Mol Life Sci.

[8] Egan B, Zierath JR (2013). Exercise metabolism and the molecular regulation of skeletal muscle adaptation. Cell Metab.

[9] Lindholm ME, Rundqvist H (2016). Skeletal muscle hypoxia-inducible factor-1 and exercise. Exp Physiol.

[10] Hawley JA, Leckey JJ (2015). Carbohydrate dependence during prolonged, intense endurance exercise. Sports Med.

[11] Petrany MJ (2020). Single-nucleus RNA-seq identifies transcriptional heterogeneity in multinucleated skeletal myofibers. Nat Commun.

[12] Ding C (2025). Glucose-responsive PAGR1-regulated skeletal muscle gene program controls systemic glucose homeostasis and hepatic metabolism. Adv Sci (Weinh).

[13] Field JT (2025). The mitophagy receptor BNIP3L/Nix coordinates nuclear calcium signaling to modulate the muscle phenotype. Autophagy.

[14] Koh MY, Powis G (2012). Passing the baton: the HIF switch. Trends Biochem Sci.

[15] Lee JW (2004). Hypoxia-inducible factor (HIF-1) alpha: its protein stability and biological functions. Exp Mol Med.

[16] Ito K, Suda T (2014). Metabolic requirements for the maintenance of self-renewing stem cells. Nat Rev Mol Cell Biol.

[17] Bensaad K (2014). Fatty acid uptake and lipid storage induced by HIF-1α contribute to cell growth and survival after hypoxia-reoxygenation. Cell Rep.

[18] Qiu B (2015). HIF2α-dependent lipid storage promotes endoplasmic reticulum homeostasis in clear-cell renal cell carcinoma. Cancer Discov.

[19] Schiaffino S, Reggiani C (2011). Fiber types in mammalian skeletal muscles. Physiol Rev.

[20] Tanner CJ (2002). Muscle fiber type is associated with obesity and weight loss. Am J Physiol Endocrinol Metab.

[21] Stuart CA (2013). Slow-twitch fiber proportion in skeletal muscle correlates with insulin responsiveness. J Clin Endocrinol Metab.

[22] Lin J (2002). Transcriptional co-activator PGC-1 alpha drives the formation of slow-twitch muscle fibres. Nature.

[23] Bodine SC (2001). Identification of ubiquitin ligases required for skeletal muscle atrophy. Science.

[24] Landes T (2010). The BH3-only Bnip3 binds to the dynamin Opa1 to promote mitochondrial fragmentation and apoptosis by distinct mechanisms. EMBO Rep.

[25] Cockmann ME (2019). Lack of activity of recombinant HIF prolyl hydroxylases (PHDs) on reported non-HIF substrates. Elife.

[26] Livet J (2007). Transgenic strategies for combinatorial expression of fluorescent proteins in the nervous system. Nature.

[27] Sohal VS (2009). Parvalbumin neurons and gamma rhythms enhance cortical circuit performance. Nature.

[28] Albadari N (2019). The transcriptional factors HIF-1 and HIF-2 and their novel inhibitors in cancer therapy. Expert Opin Drug Discov.

[29] Pappalardi MB (2011). Biochemical characterization of human HIF hydroxylases using HIF protein substrates that contain all three hydroxylation sites. Biochem J.

[30] Minamishima YA (2009). A feedback loop involving the Phd3 prolyl hydroxylase tunes the mammalian hypoxic response in vivo. Mol Cell Biol.

[31] Keith B (2012). HIF1α and HIF2α: sibling rivalry in hypoxic tumour growth and progression. Nat Rev Cancer.

[32] Haase VH (2001). Vascular tumors in livers with targeted inactivation of the von Hippel-Lindau tumor suppressor. Proc Natl Acad Sci U S A.

[33] Kapitsinou PP, Haase VH (2008). The VHL tumor suppressor and HIF: insights from genetic studies in mice. Cell Death Differ.

[34] Jacobson LO (1957). Role of the kidney in erythropoiesis. Nature.

[35] Naughton BA (1977). Hepatic regeneration and erythropoietin production in the rat. Science.

[36] Takeda K (2008). Regulation of adult erythropoiesis by prolyl hydroxylase domain proteins. Blood.

[37] Minamishima YA (2008). Somatic inactivation of the PHD2 prolyl hydroxylase causes polycythemia and congestive heart failure. Blood.

[38] Kim WY (2006). Failure to prolyl hydroxylate hypoxia-inducible factor alpha phenocopies VHL inactivation in vivo. EMBO J.

[39] Minamishima YA, Kaelin WG (2010). Reactivation of hepatic EPO synthesis in mice after PHD loss. Science.

[40] Katz O (2010). Erythropoietin treatment leads to reduced blood glucose levels and body mass: insights from murine models. J Endocrinol.

[41] Heinicke K (2006). Excessive erythrocytosis in adult mice overexpressing erythropoietin leads to hepatic, renal, neuronal, and muscular degeneration. Am J Physiol Regul Integr Comp Physiol.

[42] Yin WQ (2024). Erythropoietin regulates energy metabolism through EPO-EpoR-RUNX1 axis. Nat Commun.

[43] Zhang Y (2017). Sex difference in mouse metabolic response to erythropoietin. FASEB J.

[44] Slot IGM (2014). Hypoxia differentially regulates muscle oxidative fiber type and metabolism in a HIF-1α-dependent manner. Cell Signal.

[45] Mason SD (2004). Loss of skeletal muscle HIF-1alpha results in altered exercise endurance. PLoS Biol.

[46] Gupta N, Wish JB (2017). Hypoxia-inducible factor prolyl hydroxylase inhibitors: a potential new treatment for anemia in patients with CKD. Am J Kidney Dis.

[47] Carney EF (2016). Therapy: PHD inhibitors correct anaemia in CKD. Nat Rev Nephrol.

[48] Chou YH (2023). Pleotropic effects of hypoxia-inducible factor-prolyl hydroxylase domain inhibitors: are they clinically relevant?. Kidney Res Clin Pract.

[49] Yang MS (2022). Non-invasive administration of AAV to target lung parenchymal cells and develop SARS-CoV-2-susceptible mice. Mol Ther.

[50] Hu CJ (2007). The N-terminal transactivation domain confers target gene specificity of hypoxia-inducible factors HIF-1alpha and HIF-2alpha. Mol Biol Cell.

[51] Tabebordbar M (2021). Directed evolution of a family of AAV capsid variants enabling potent muscle-directed gene delivery across species. Cell.

[52] Spinazzi M (2012). Assessment of mitochondrial respiratory chain enzymatic activities on tissues and cultured cells. Nat Protoc.

[53] Sheth KA (2018). Muscle strength and size are associated with motor unit connectivity in aged mice. Neurobiol Aging.

