# Supplementary material for: Distinct HIF1α and HIF2α functions control skeletal muscle metabolism and erythropoiesis
Source: J Clin Invest. 2026 Feb 17;136(8):e195411. doi: 10.1172/JCI195411 (PMC13078885; doi:10.1172/JCI195411)
Supplement: Supplemental data [file jci-136-195411-s096.pdf]

Supplemental Figure. 1

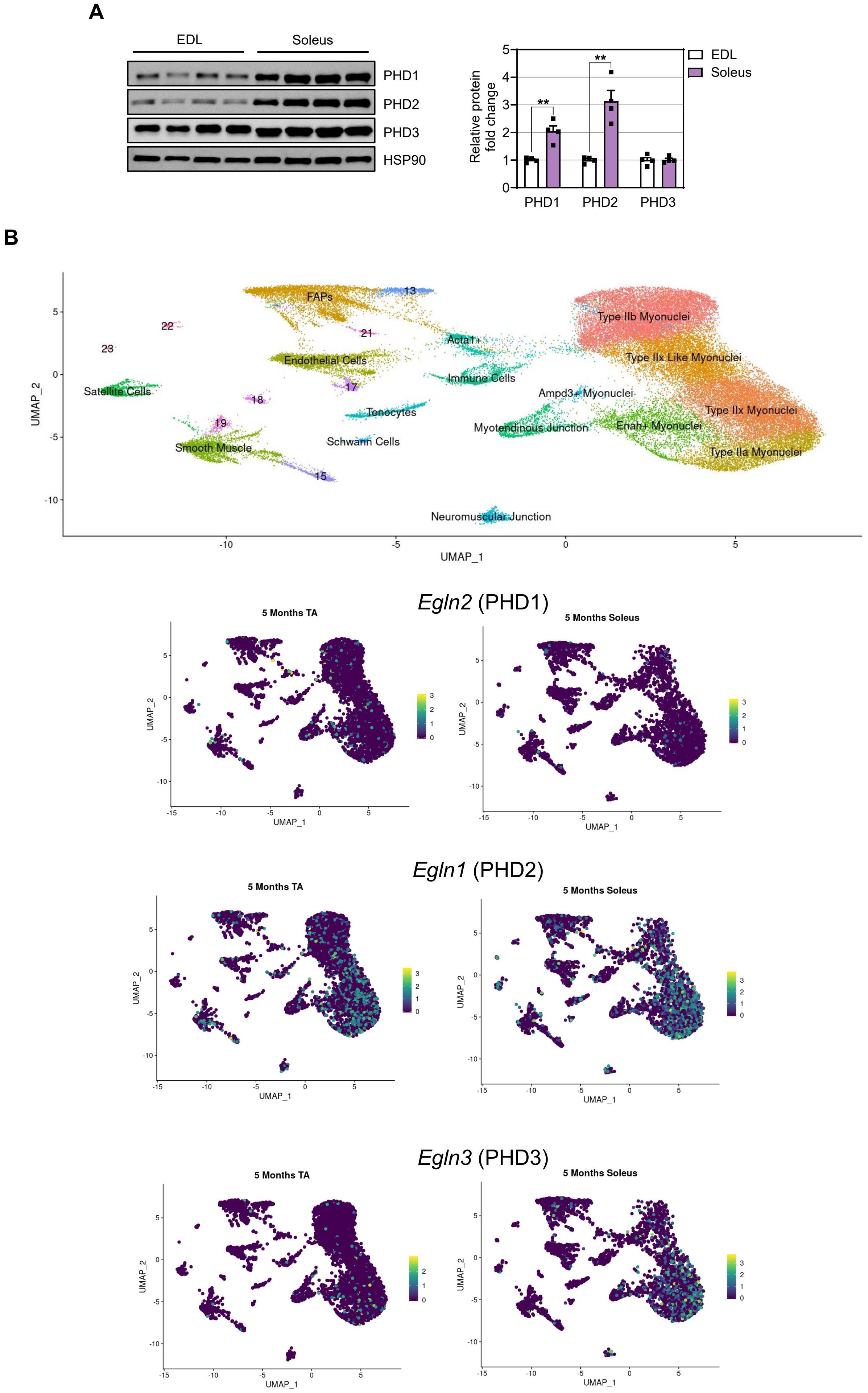

Supplemental Figure. 2

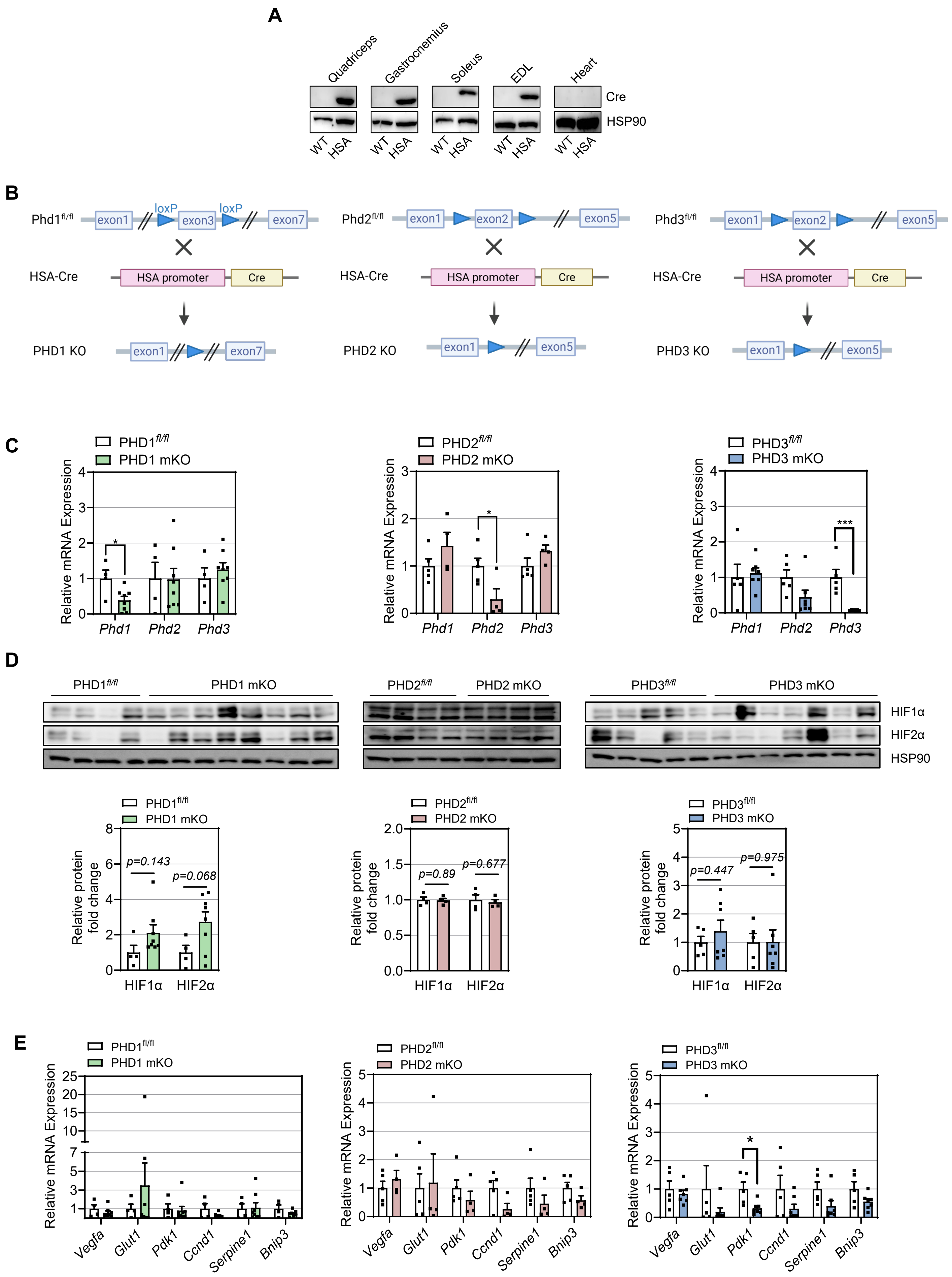

Supplemental Figure. 3

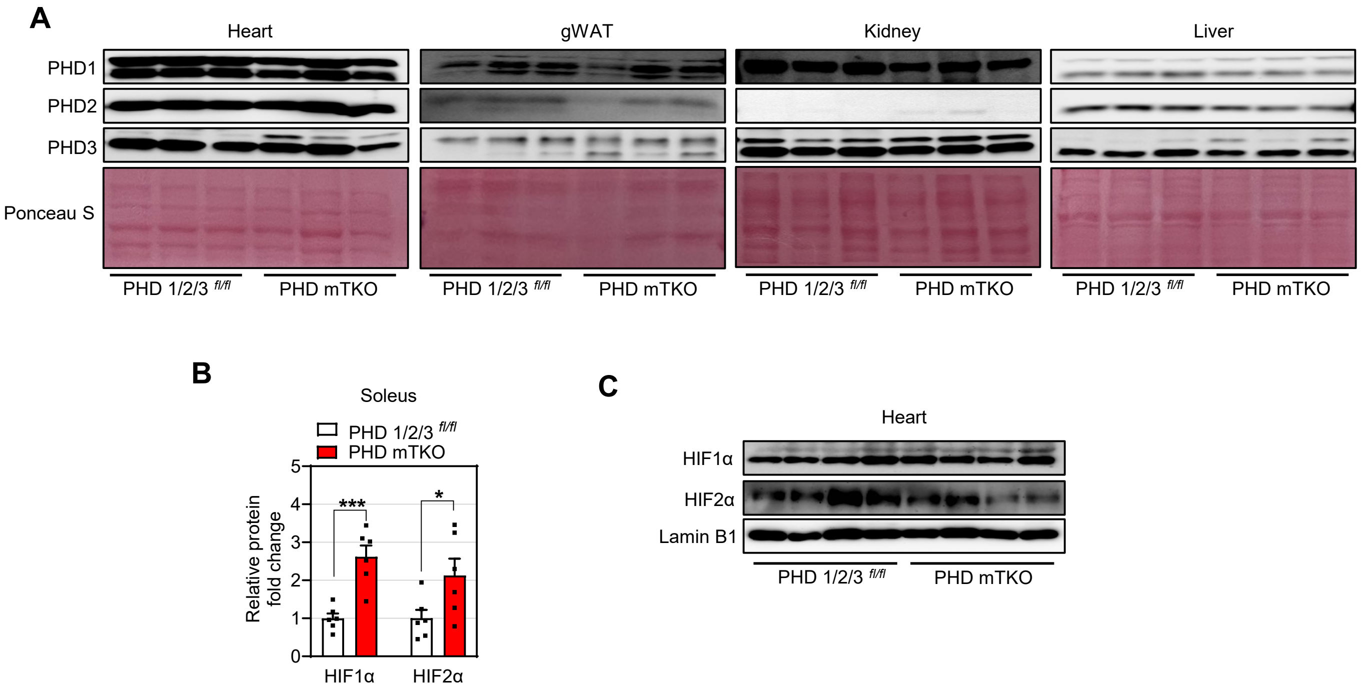

Supplemental Figure. 4

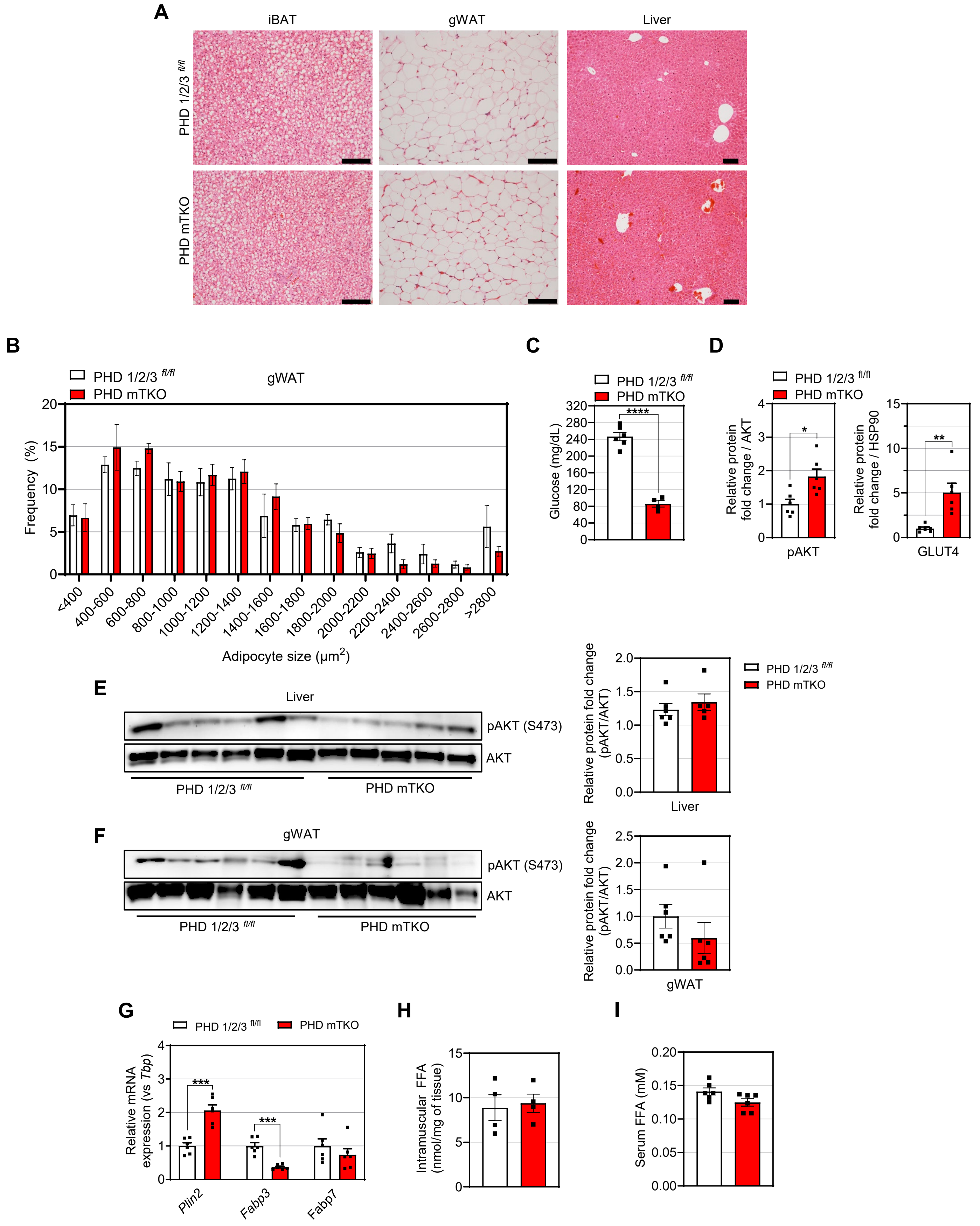

Supplemental Figure. 5

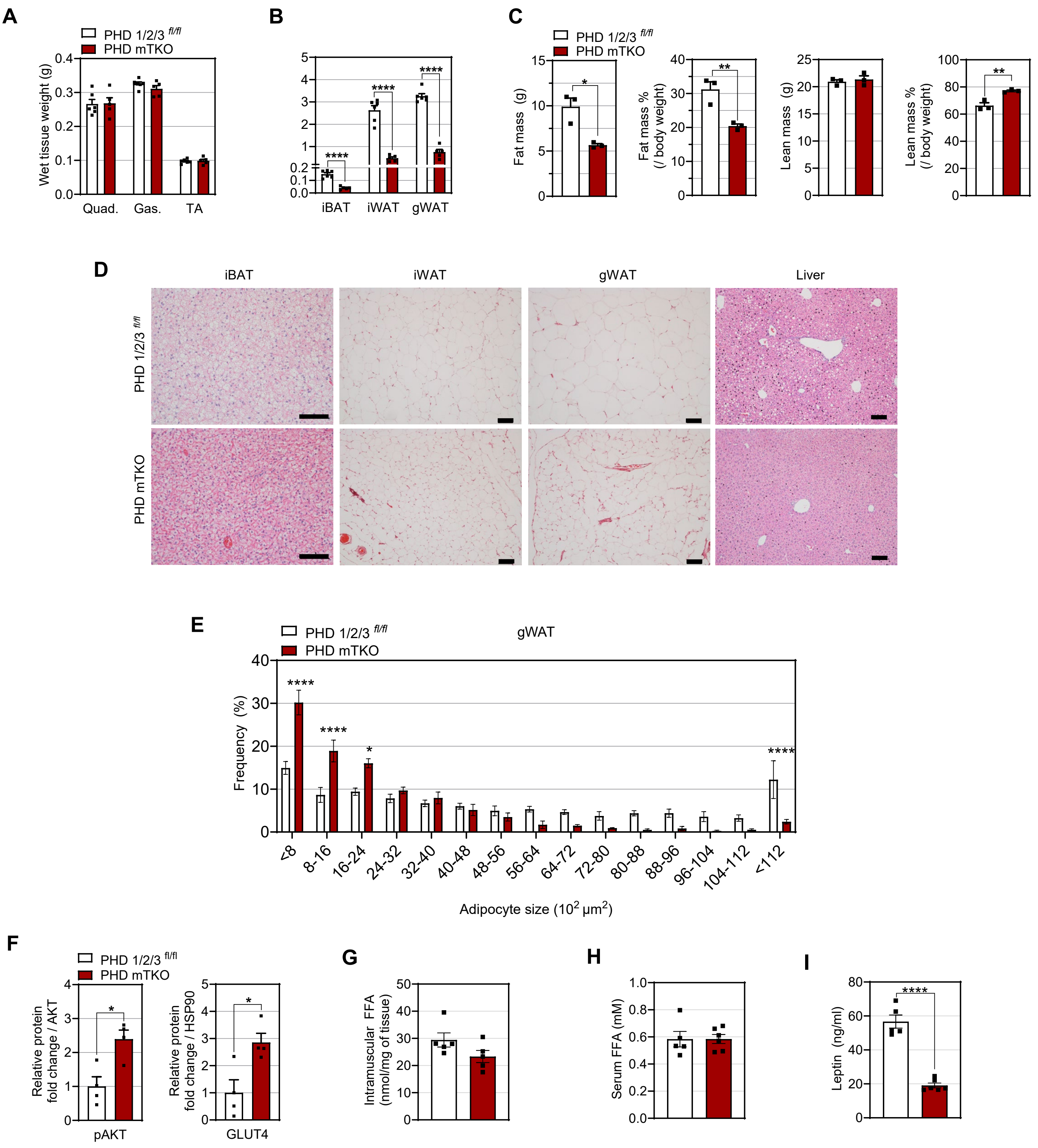

Supplemental Figure. 6

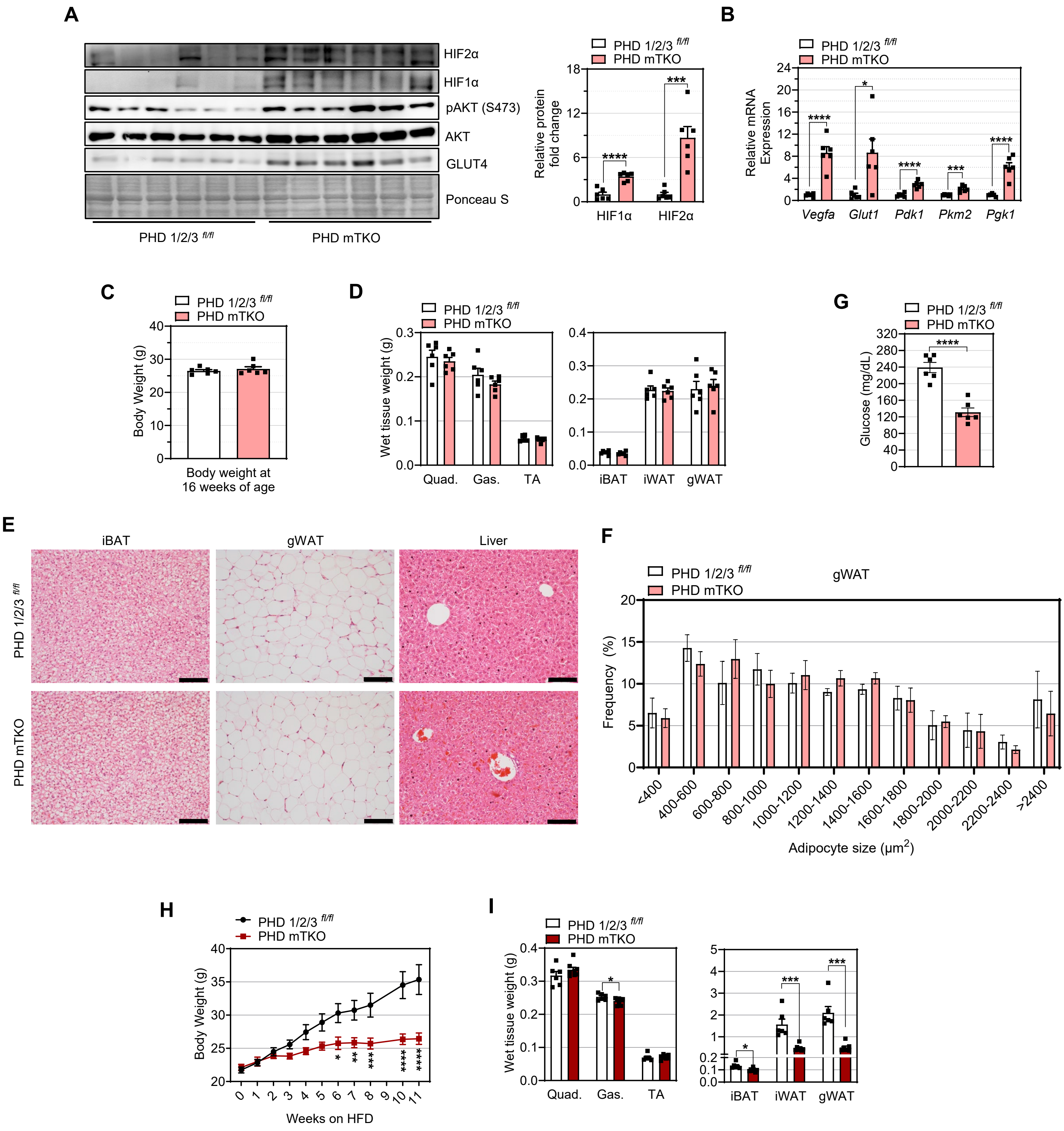

Supplemental Figure. 7

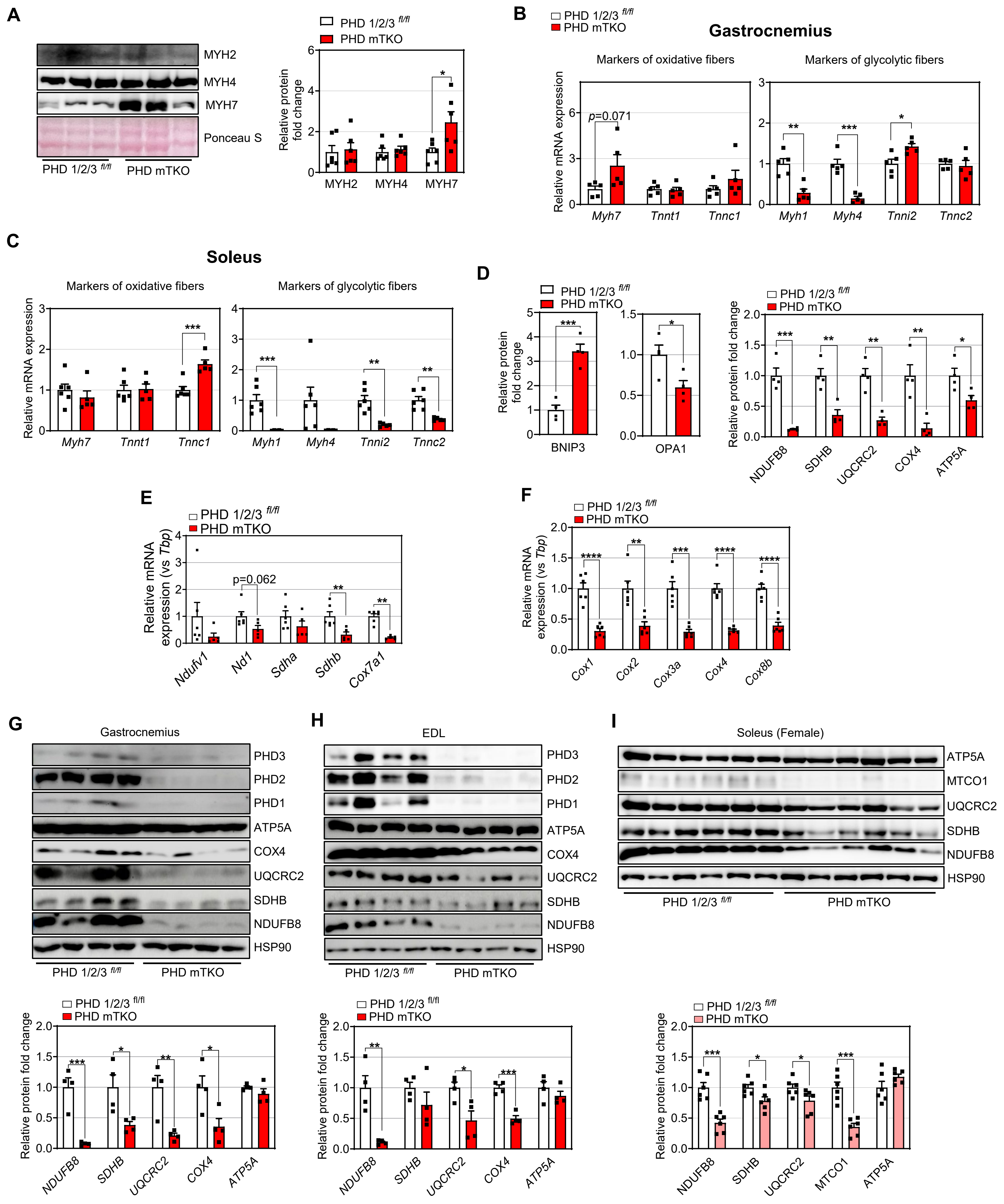

Supplemental Figure. 8

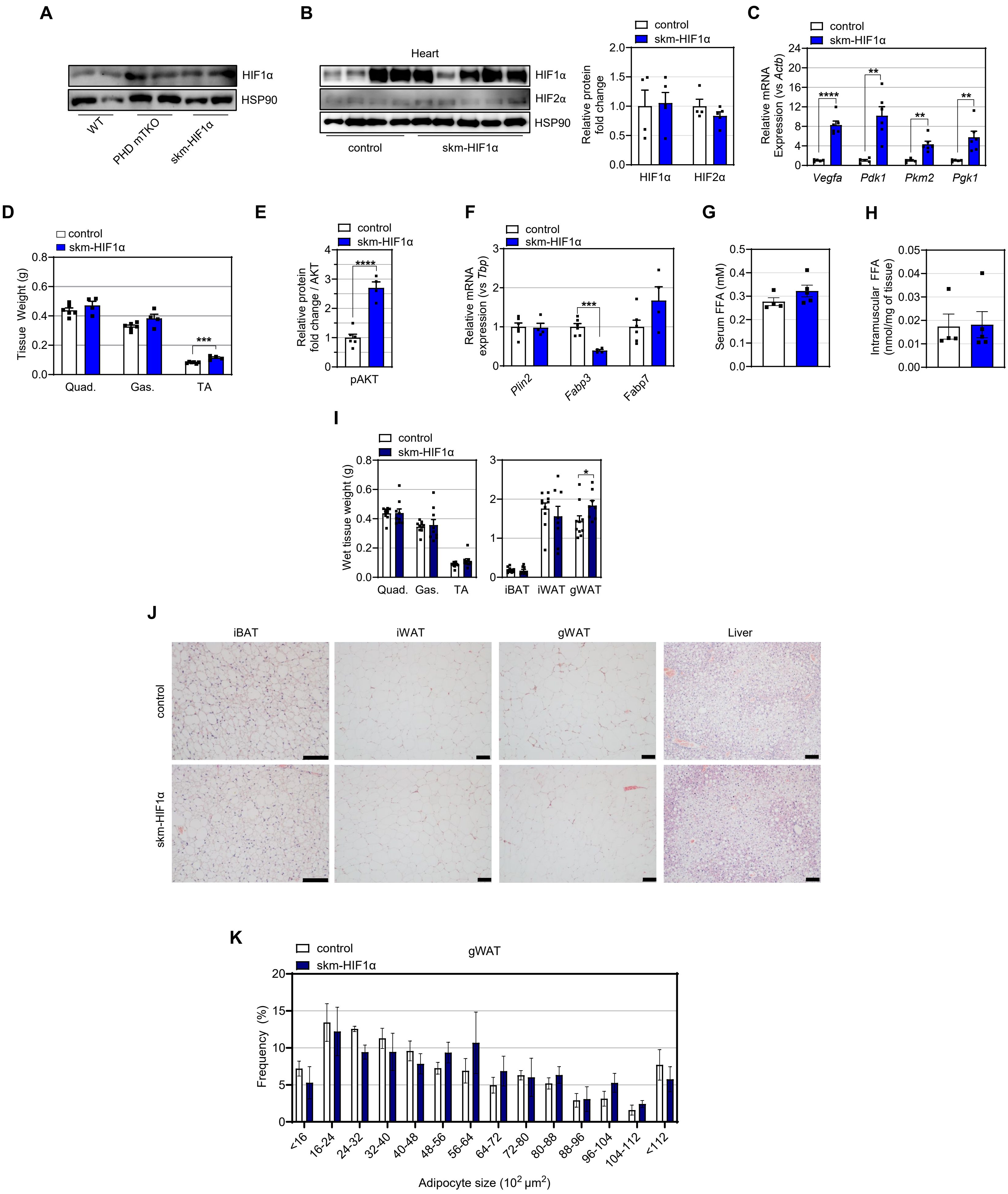

Supplemental Figure. 9

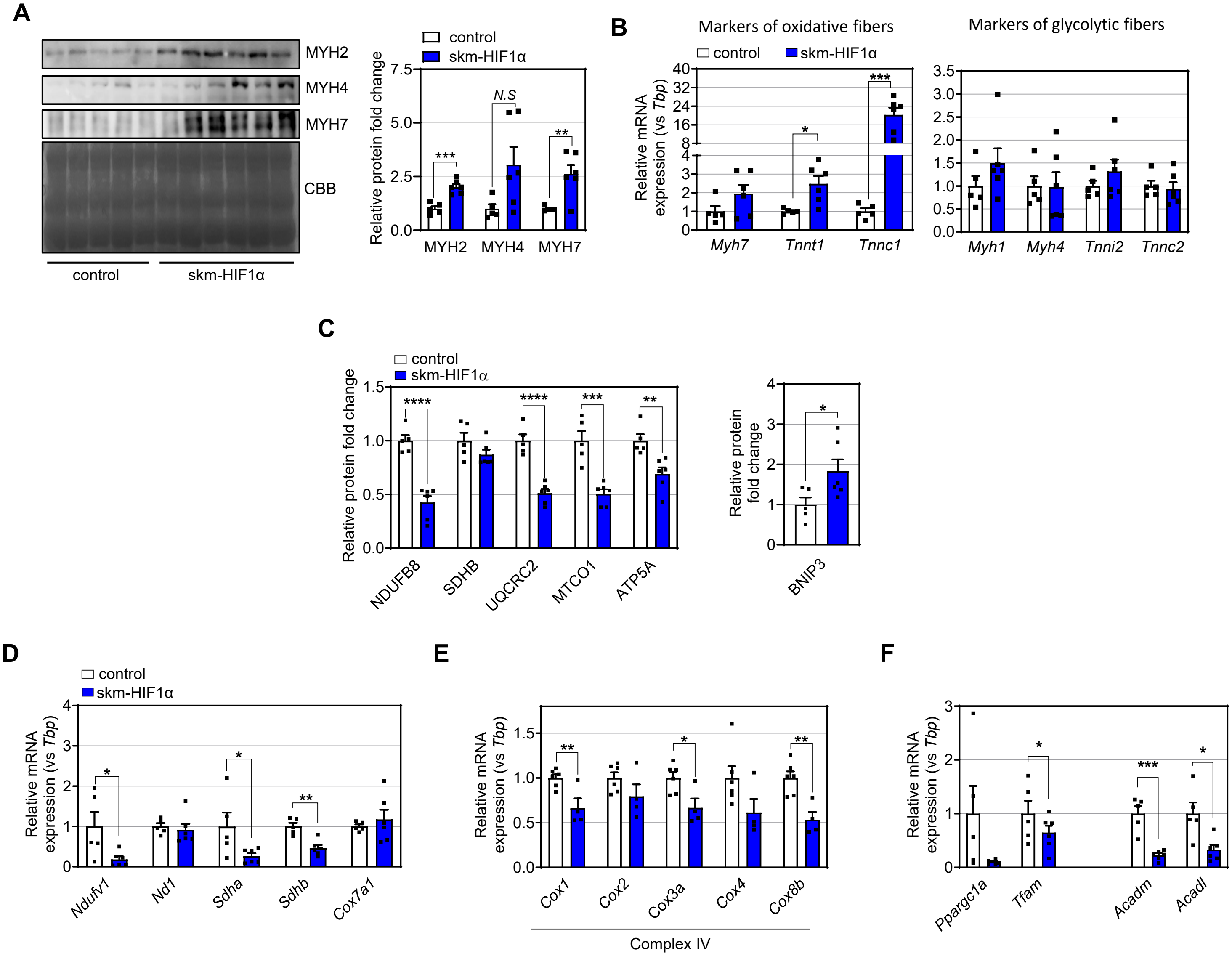

Supplemental Figure. 10

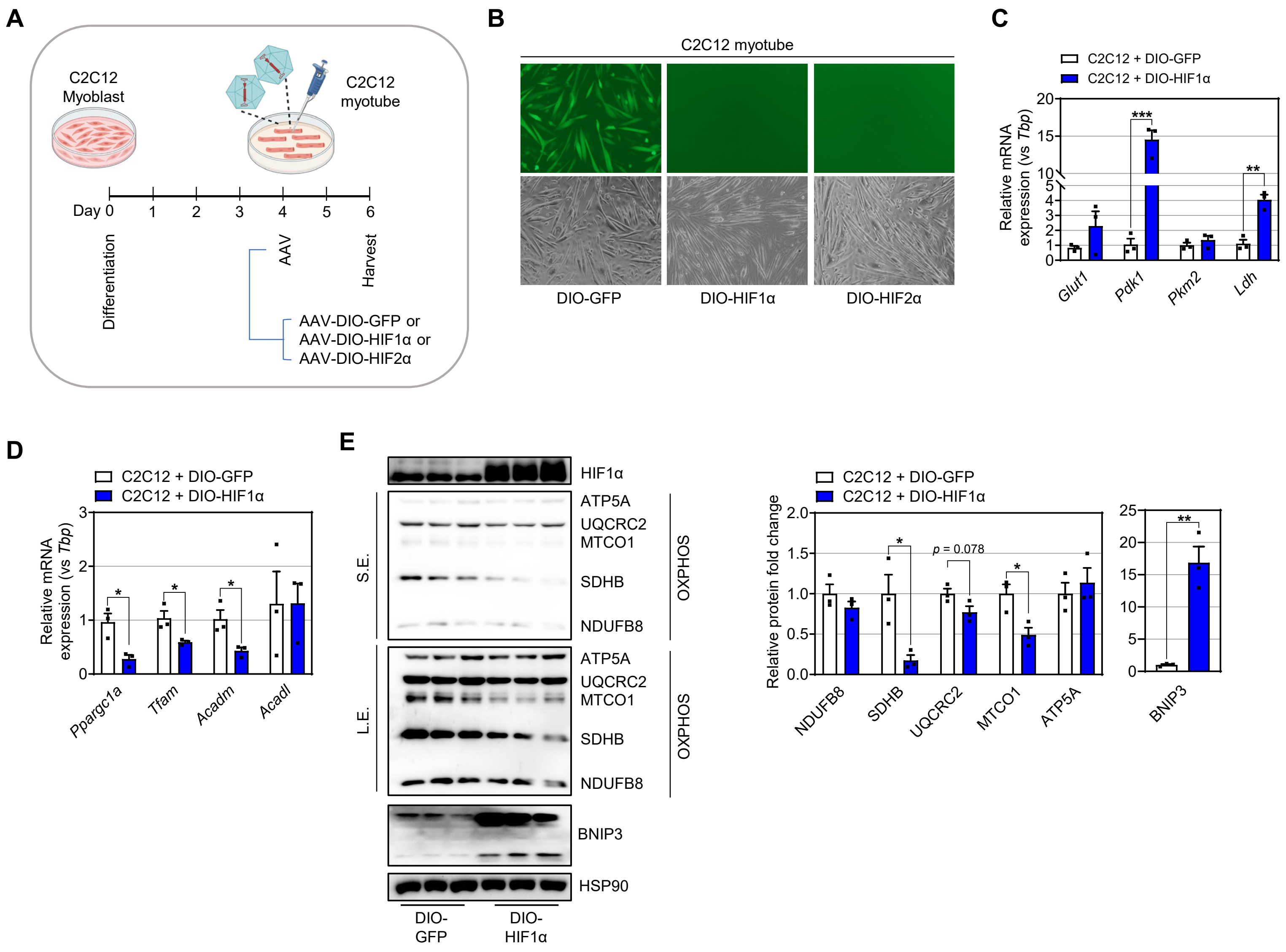

Supplemental Figure. 11

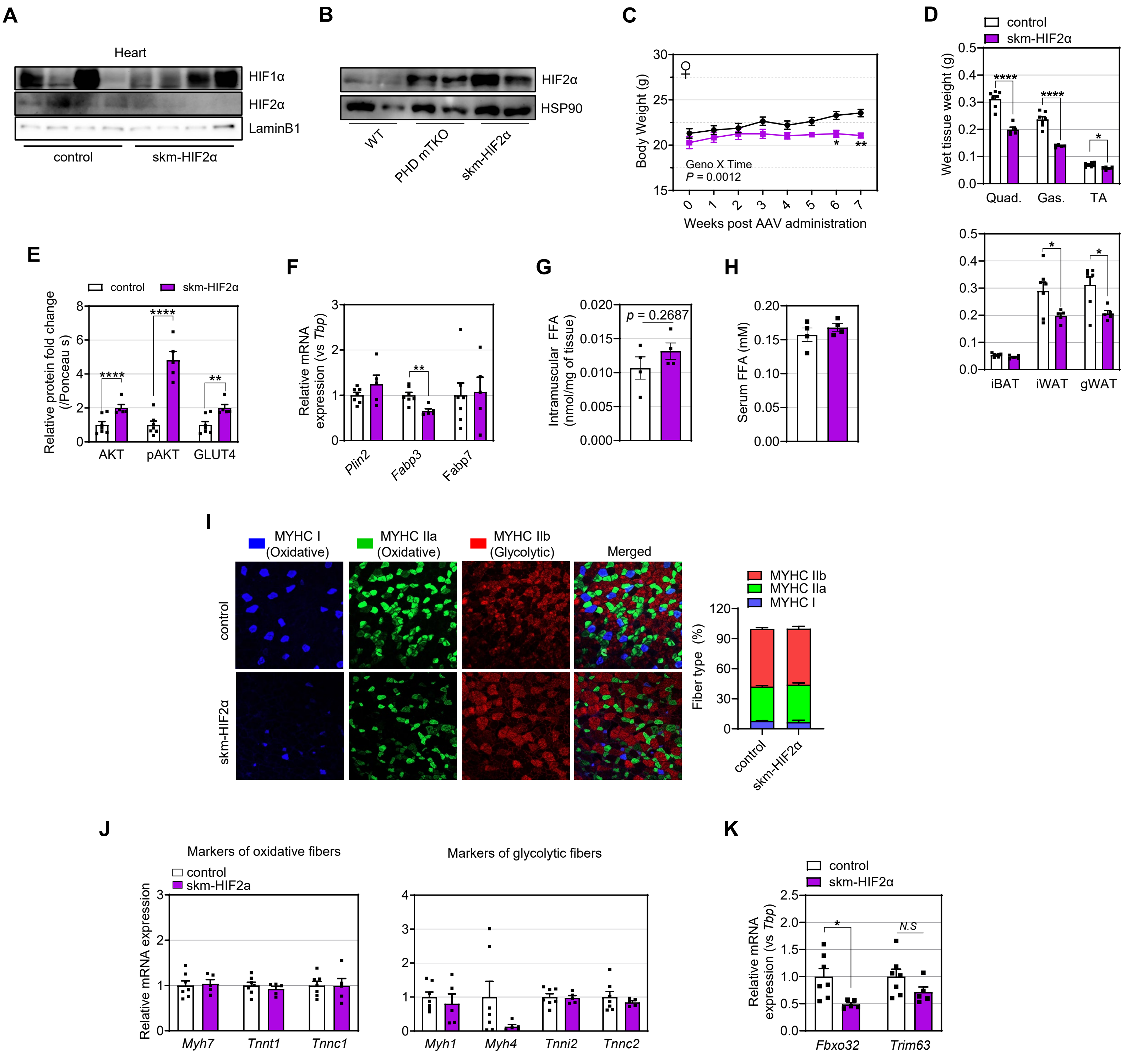

Supplemental Figure. 12

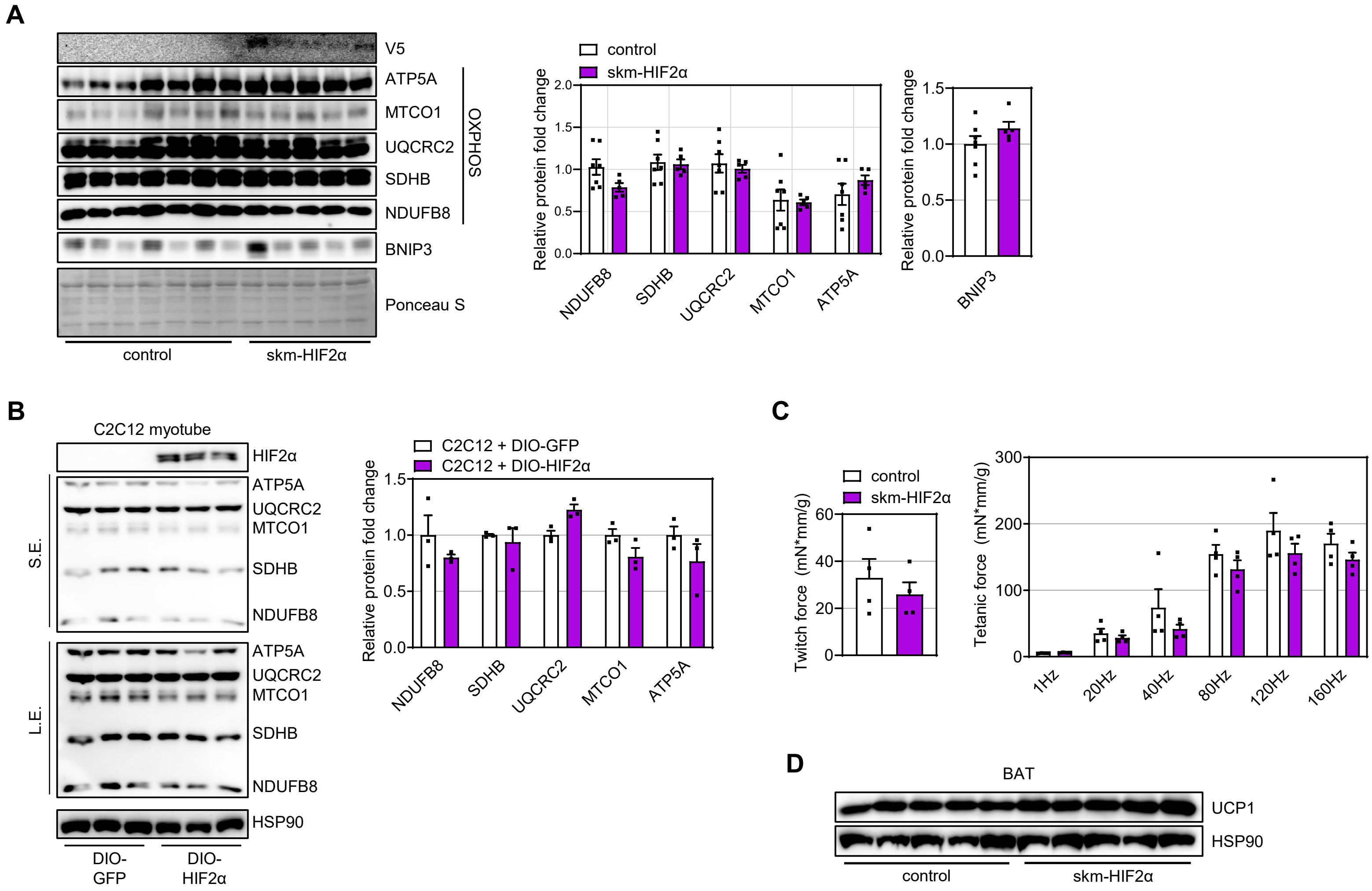

Supplemental Figure. 13

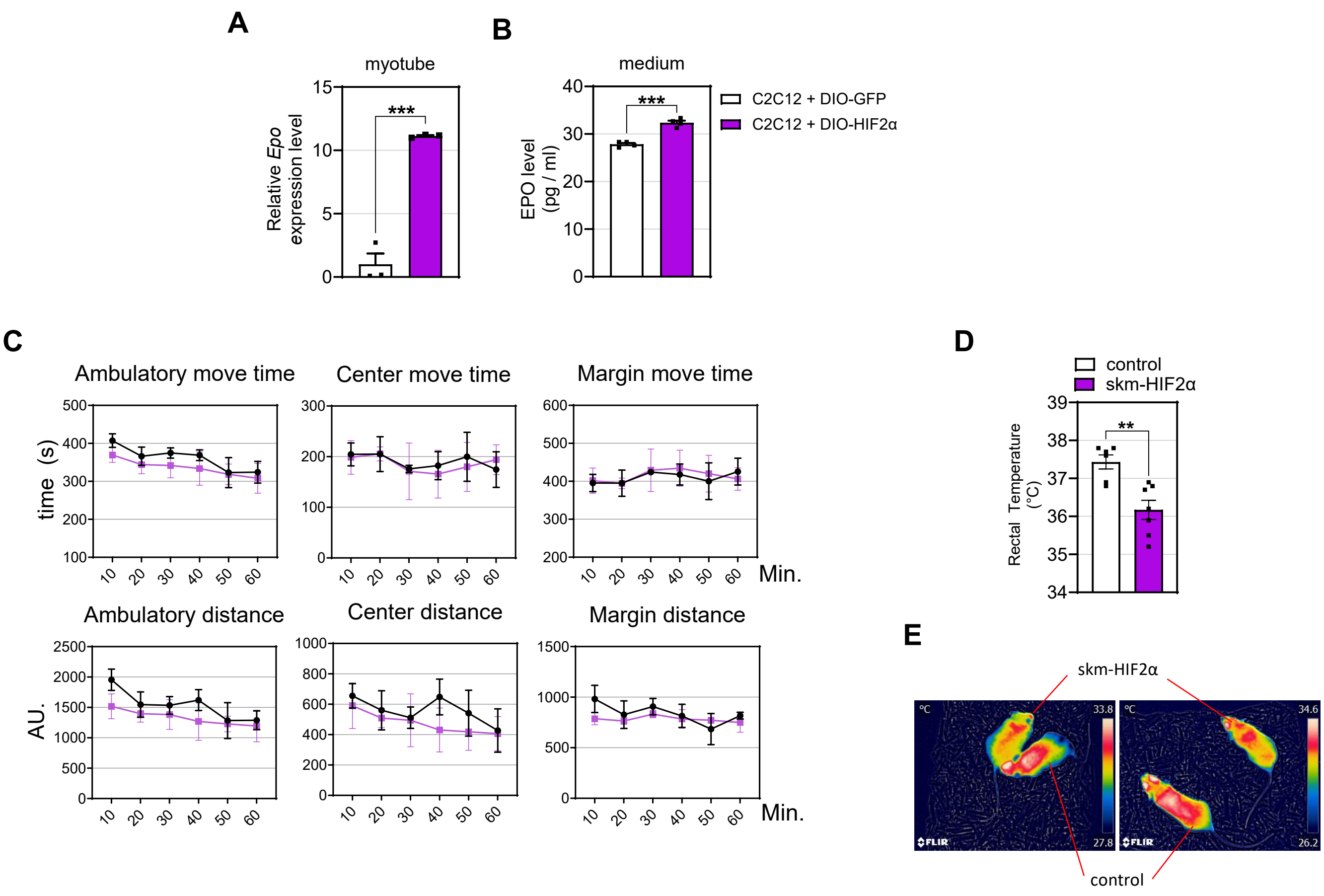

Supplemental Figure. 14

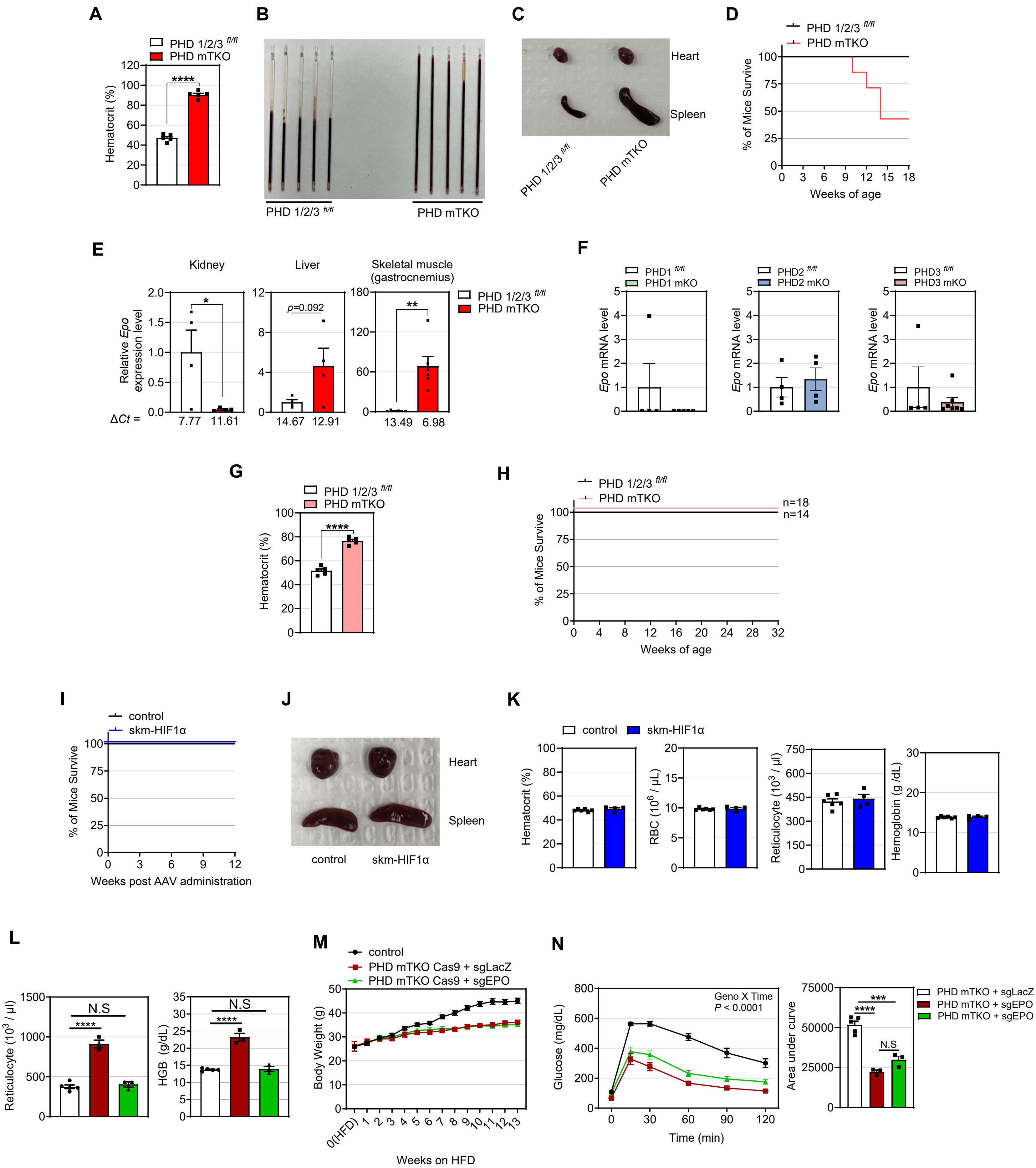

Supplemental Figure. 15

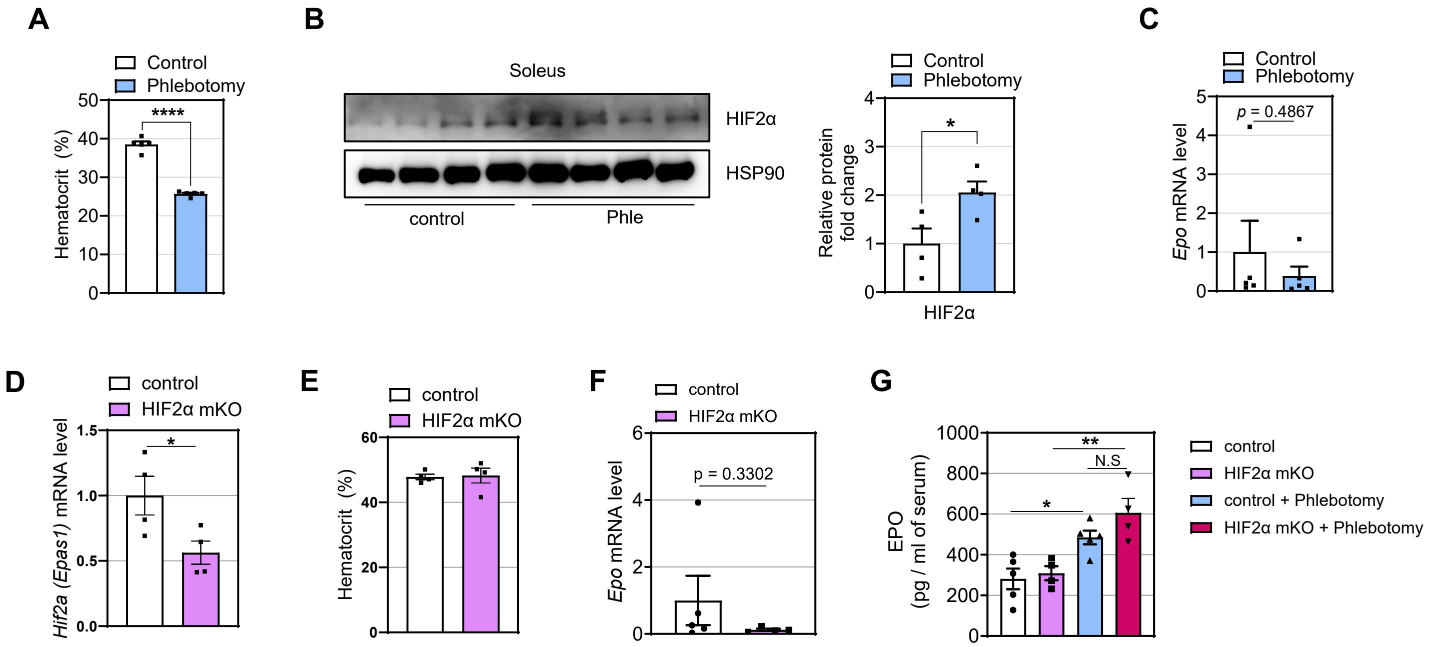

Supplemental Figure 1. Expression profiles of prolyl hydroxylase domain (PHD) enzymes.

(A) Immunoblot results from the extensor digitorum longus (EDL) or soleus from C57BL/6 mice (left) and quantification of the indicated proteins (right) (n = 4). **(B)** Single-nuclei RNA sequencing results from Myoatlas. Data are shown as the mean ± SEM. Unpaired two-tailed Student's t-test was used for statistical analysis. $^{**}P < 0.01$.

Supplemental Figure 2. Generation of skeletal muscle-specific single-PHD knockout mice.

(A) Representative immunoblots of Cre in wild-type (WT) and HSA-Cre**.** **(B)** Schematic showing the PHD1 mKO, PHD2 mKO and PHD3 mKO mice (myofiber-specific deletion of PHD1 or PHD2 or PHD3, respectively). **(C)** Relative mRNA expression in PHD mKO and control mice (PHD1 $^{fl/fl}$, n = 4; PHD1 mKO, n = 8; PHD2 $^{fl/fl}$, n = 5; PHD2 mKO, n = 4; PHD3 $^{fl/fl}$, n = 5; PHD3 mKO, n = 7). **(D)** Immunoblot analysis of the soleus from the indicated mice (top) and relative density of HIF1α and HIF2α compared with HSP90 levels (bottom) (PHD1 $^{fl/fl}$, n = 4; PHD1 mKO, n = 8; PHD2 $^{fl/fl}$, n = 4; PHD2 mKO, n = 4; PHD3 $^{fl/fl}$, n = 5; PHD3 mKO, n = 7). **(E)** mRNA expression levels of the HIFα target genes in the soleus from the indicated mice (PHD1 $^{fl/fl}$, n = 4; PHD1 mKO, n = 8; PHD2 $^{fl/fl}$, n = 5; PHD2 mKO, n = 4; PHD3 $^{fl/fl}$, n = 5; PHD3 mKO, n = 7). Data are shown as the mean ± SEM. Unpaired two-tailed Student's t-test was used for statistical analysis. $^{*}P < 0.05$ and $^{***}P < 0.001$. N.S., not significant.

Supplemental Figure 3. Confirmation of muscle-specific ablation of PHDs.

(A) Immunoblot analysis of heart, gWAT, kidney, and liver tissues from PHD mTKO and control mice (PHD1/2/3 $^{fl/fl}$, n = 3; PHD mTKO, n = 3). The gel blots were stained with Ponceau S to indicate the relative amount of total proteins loaded. **(B)** Quantification of HIF1α and

HIF2α related to Figure 1B (PHD1/2/3 $^{fl/fl}$, n = 4; PHD mTKO, n = 4). **(C)** Immunoblots of HIF1α and HIF2α in the heart of PHD mTKO mice (PHD1/2/3 $^{fl/fl}$, n = 4; PHD mTKO, n = 4) Data are shown as the mean ± SEM. Unpaired two-tailed Student's t-test was used for statistical analysis. *P < 0.05 and ***P < 0.001.

Supplemental Figure 4. Lipid accumulation and insulin sensitivity are unchanged in the PHD mTKO male mice under NCD.

(A) Histological analysis of adipose and liver tissues. Scale bar = 100 μm. **(B)** Frequency of adipocyte sizes in gWAT (PHD 1/2/3 $^{fl/fl}$, n = 4; PHD mTKO, n = 4). **(C)** Non-fasting glucose levels in blood (PHD 1/2/3 $^{fl/fl}$, n = 6; PHD mTKO, n = 4). **(D)** Quantification of pAKT and GLUT4 related to Figure 1G (PHD 1/2/3 $^{fl/fl}$, n = 6; PHD mTKO, n = 6). **(E, F)** Immunoblots of indicated proteins (left) and quantifications (right) from the liver (**E**) or gWAT (**F**) (PHD 1/2/3 $^{fl/fl}$, n = 6; PHD mTKO, n = 5 to 6). **(G)** mRNA expression related to fatty acid uptake and storage in the soleus of PHD mTKO mice (PHD 1/2/3 $^{fl/fl}$, n = 6; PHD mTKO, n = 6). **(H)** Intramuscular free fatty acid (FFA) levels in the gastrocnemius of PHD mTKO mice (PHD 1/2/3 $^{fl/fl}$, n = 4; PHD mTKO, n = 4). **(I)** Serum FFA levels in PHD mTKO mice (PHD 1/2/3 $^{fl/fl}$, n = 6; PHD mTKO, n = 6) Data are shown as the mean ± SEM. Unpaired two-tailed Student's t-test was used for statistical analysis. *P < 0.05, **P < 0.01, ***P < 0.001, and ****P < 0.0001.

Supplemental Figure 5. PHD deficiency in skeletal muscle protects against diet-induced obesity.

(A, B) Weights of muscles **(A)** and adipose tissues **(B)** of PHD mTKO mice under HFD (PHD 1/2/3 $^{fl/fl}$, n = 6; PHD mTKO, n = 5). **(C)** Body mass of PHD mTKO mice under HFD (PHD

1/2/3 $^{fl/fl}$, n = 3; PHD mTKO, n = 3). **(D)** Histological analysis of the adipose and liver tissues. Scale bar = 100 μm. **(E)** Frequency of adipocyte sizes in gWAT (PHD 1/2/3 $^{fl/fl}$, n = 4; PHD mTKO, n = 4). **(F)** Quantification of pAKT and GLUT4 in PHD mTKO mice related to Figure 1L (PHD 1/2/3 $^{fl/fl}$, n = 4; PHD mTKO, n = 4). **(G)** Intramuscular free fatty acid (FFA) levels in the gastrocnemius of PHD mTKO mice under HFD (PHD 1/2/3 $^{fl/fl}$, n = 5; PHD mTKO, n = 5). **(H)** Serum FFA levels in PHD mTKO mice under HFD (PHD 1/2/3 $^{fl/fl}$, n = 5; PHD mTKO, n = 6). **(I)** Serum leptin levels in PHD mTKO mice under HFD (PHD 1/2/3 $^{fl/fl}$, n = 5; PHD mTKO, n = 6). Data are shown as the mean ± SEM. Unpaired two-tailed Student's t-test (**A**, **B**, **C**, **F**, **G**, **H**, **I**) and two-way ANOVA with Bonferroni's post-hoc test (**E**) was used for statistical analyses. *P < 0.05, **P < 0.01, ***P < 0.001, and ****P < 0.0001.

Supplemental Figure 6. PHD deficiency in skeletal muscles of female mice.

(A - F) Female PHD 1/2/3 $^{fl/fl}$ or PHD mTKO mice were administered a normal chow diet (NCD). **(A)** Representative immunoblots of the indicated proteins from the soleus (left) and quantification of HIF1α and HIF2α (right) (PHD 1/2/3 $^{fl/fl}$, n = 6; PHD mTKO, n = 6). **(B)** mRNA levels of the indicated HIFα target genes in the soleus (PHD 1/2/3 $^{fl/fl}$, n = 6; PHD mTKO, n = 6). **(C)** Body weights of mice on an NCD (PHD 1/2/3 $^{fl/fl}$, n = 6; PHD mTKO, n = 6). **(D)** Weights of muscles and adipose tissues of mice on an NCD (PHD 1/2/3 $^{fl/fl}$, n = 6; PHD mTKO, n = 6). **(E)** Histological analysis of the adipose and liver tissues. Scale bar = 100 μm. **(F)** Frequency of adipocyte sizes in gWAT (PHD 1/2/3 $^{fl/fl}$, n = 4; PHD mTKO, n = 4). **(G)** Non-fasting glucose levels in blood (PHD 1/2/3 $^{fl/fl}$, n = 6; PHD mTKO, n = 6). **(H)** Body weights of female mice on an HFD (PHD 1/2/3 $^{fl/fl}$, n = 6; PHD mTKO, n = 8). **(I)** Weights of muscles and adipose tissues of mice on an HFD (PHD 1/2/3 $^{fl/fl}$, n = 6; PHD mTKO, n = 7–8). Data are shown as the mean ± SEM. Unpaired two-tailed Student's t-test (**A, B, C, D, G, I**) or ordinary

(F) or repeated-measured **(H)** two-way ANOVA with Bonferroni's post-hoc test was used for statistical analyses. $*P < 0.05$, $**P < 0.01$, $***P < 0.001$, and $****P < 0.0001$.

Supplemental Figure 7. PHD deficiency in skeletal muscle increases oxidative fiber proportion.

(A) Immunoblots of the indicated proteins (left) and quantification (right) (PHD 1/2/3 $^{fl/fl}$, n = 6; PHD mTKO, n = 6). **(B, C)** mRNA expression levels of oxidative or glycolytic fiber markers in the gastrocnemius (**B**) or soleus (**C**) of mice (PHD 1/2/3 $^{fl/fl}$, n = 5; PHD mTKO, n = 5). **(D)** Quantification of indicated proteins in PHD mTKO related to Figure 2J (PHD 1/2/3 $^{fl/fl}$, n = 4; PHD mTKO, n = 4). **(E, F)** mRNA expression of the indicated genes (PHD 1/2/3 $^{fl/fl}$, n = 6; PHD mTKO, n = 5–6). **(G – I)** Representative immunoblots of the indicated proteins (top) and quantification (bottom) in the gastrocnemius **(G)** or EDL **(H)** of male PHD mTKO mice (PHD 1/2/3 $^{fl/fl}$, n = 4; PHD mTKO, n = 4) or the soleus **(I)** of female PHD mTKO mice (PHD 1/2/3 $^{fl/fl}$, n = 6; PHD mTKO, n = 6). Data are shown as the mean ± SEM. Unpaired two-tailed Student's t-test was used for statistical analysis. $*P < 0.05$, $**P < 0.01$, $***P < 0.001$, and $****P < 0.0001$.

Supplemental Figure 8. HIF1α stabilization improves glucose tolerance but does not reduce weight gain.

(A) Immunoblot of HIF1α in the soleus from skm-HIF1α mice compared with PHD mTKO mice. **(B)** Immunoblot of HIF1α and HIF2α (left) and quantification (right) in the heart of skm-HIF1α mice (control, n = 4; skm-HIF1α, n = 5). **(C)** mRNA expression of the indicated genes (control, n = 5; skm-HIF1α, n = 6). **(D)** Weights of muscles from skm-HIF1α mice on an NCD

(control, n = 5; skm-HIF1α, n = 4). **(E)** Quantification of pAKT levels in the soleus of skm-HIF1α mice, related to Figure 3F (control, n = 6; skm-HIF1α, n = 4). **(F)** mRNA expression levels related to fatty acid uptake and storage in the soleus of skm-HIF1α mice (control, n = 6; skm-HIF1α, n = 4). **(G)** Serum free fatty acid levels in skm-HIF1α mice (control, n = 4; skm-HIF1α, n = 5**). (H)** Intramuscular free fatty acid levels in gastrocnemius of skm-HIF1α mice (control, n = 4; skm-HIF1α, n = 5). **(I)** Weights of muscles and adipose tissues from skm-HIF1α mice on an HFD (control, n = 10; skm-HIF1α, n = 8 – 9). **(J)** Histological analysis of the adipose and liver tissues. Scale bar = 100 μm. **(K)** Frequency of adipocyte sizes in gWAT (control, n = 4; skm-HIF1α, n = 3). Data are shown as the mean ± SEM. Unpaired two-tailed Student's t-test (**B–I**) or two-way ANOVA **(K)** with Bonferroni's post-hoc test was used for statistical analysis. *$P < 0.05$, **$P < 0.01$, ***$P < 0.001$, and ****$P < 0.0001$.

Supplemental Figure 9. HIF1α in skeletal muscle increases oxidative fiber proportion but impairs mitochondrial activity.

(A) Immunoblots of the indicated proteins from the gastrocnemius of mice (left) and quantification (right) (control, n = 6; skm-HIF1α, n = 5). **(B)** mRNA expression levels of oxidative or glycolytic fiber markers in the gastrocnemius (control, n = 5; skm-HIF1α, n = 6). **(C)** Quantification of the indicated proteins levels in the soleus of skm-HIF1α mice, related to Figure 4H (control, n = 5; skm-HIF1α, n = 6). **(D)** mRNA expression levels related to the mitochondrial electron transport chain in the soleus of skm-HIF1α mice (control, n = 5; skm-HIF1α, n = 6). (**E)** mRNA expression levels related to the mitochondrial complex IV (control, n = 6; skm-HIF1α, n = 4). **(F)** mRNA expression levels in the soleus of skm-HIF1α mice (control, n = 5; skm-HIF1α, n = 6). Data are shown as the mean ± SEM. Unpaired two-tailed Student's t-test was used for statistical analysis. *$P < 0.05$, **$P < 0.01$, ***$P < 0.001$, ****P

< 0.0001.

Supplemental Figure 10. Stabilized HIF1α transduction in C2C12 myotubes impairs mitochondrial oxidative phosphorylation.

(A) Schematic illustration of C2C12 myotubes transduced with HIF1α TM or HIF2α TM. **(B)** Differential interference contrast images of C2C12 myotubes transduced with AAV-DIO-GFP or AAV-DIO-HIF1α TM or AAV-DIO-HIF2α TM. **(C)** mRNA expression levels of genes related to HIF1α targets. **(D)** mRNA expression levels of indicated genes. **(E)** Immunoblot of the indicated proteins (left) and quantification (right) (C2C12 + DIO-GFP, n = 3; C2C12 + DIO-HIF1α TM, n = 3). Data are shown as the mean ± SEM. Unpaired two-tailed Student's t-test was used for statistical analysis. *$P < 0.05$, **$P < 0.01$, ***$P < 0.001$.

Supplemental Figure 11. HIF2α in skeletal muscle mitigates body weight gain.

(A) Immunoblot of HIF1α and HIF2α in the heart from skm-HIF2α mice (control, n = 4; skm-HIF2α, n = 4). **(B)** Immunoblot of HIF2α in the soleus from skm-HIF2α mice compared with PHD mTKO mice. (**C**) Body weights of mice on an NCD (control, n = 8; skm-HIF2α, n = 6). (**D)** Weights of muscles and adipose tissues of mice on an NCD (control, n = 7; skm-HIF2α, n = 5). **(E)** Quantifications of AKT, pAKT, and GLUT4 in skm-HIF2α mice related to Figure 5F (control, n = 7; skm-HIF2α, n = 5). **(F)** mRNA expression levels of the indicated genes in the soleus of skm-HIF2α mice (control, n = 7; skm-HIF2α, n = 5). **(G)** Intramuscular free fatty acid levels in the gastrocnemius of skm-HIF2α mice (control, n = 4; skm-HIF2α, n = 4). **(H)** Serum free fatty acid levels in skm-HIF2α mice (control, n = 4; skm-HIF2α, n = 4). **(I)** Representative images of immunofluorescence staining from the gastrocnemius. The ratio of each myofiber

type composition (control, n = 4; skm-HIF2α, n = 4). **(J)** mRNA expression levels of oxidative or glycolytic fiber markers in gastrocnemius (control, n = 7; skm-HIF2α, n = 5). **(K)** mRNA expression levels of muscle atrophic genes (control, n = 7; skm-HIF2α, n = 5). Data are shown as the mean ± SEM. Unpaired two-tailed Student's t-test (**D, E, F, G, H, J, K**), or two-way ANOVA with Bonferroni's post-hoc test (**C**) was used for statistical analysis. *$P < 0.05$, **$P < 0.01$, ****$P < 0.001$.

Supplemental Figure 12. HIF2α in skeletal muscle has a negligible effect on mitochondrial oxidative phosphorylation.

(A) Immunoblot (left) and quantifications (right) of mitochondria-related proteins in the soleus of skm-HIF2α mice (control, n = 7; skm-HIF2α, n = 5). **(B)** Immunoblot (left) and quantifications (right) of OXPHOS in C2C12 myotubes transduced with AAV-DIO-HIF2α TM (C2C12 + DIO-GFP, n = 3; C2C12 + DIO-HIF2α TM, n = 3). **(C)** Twitch force and tetanic plantar flexion torque normalized to body weight (control, n = 4; skm-HIF2α, n = 4). **(D)** UCP1 protein levels in the iBAT of skm-HIF2α mice (control, n = 5; skm-HIF2α, n = 5). Data are shown as the mean ± SEM. Unpaired two-tailed Student's t-test **(A, B, C, D)** or two-way ANOVA with Bonferroni's post-hoc test (**C**) was used for statistical analysis.

Supplemental Figure 13. HIF2α-driven muscle EPO impairs thermal homeostasis

(A) mRNA expression levels of the *Epo* gene in the C2C12 myotube transduced with AAV-DIO-HIF2α TM (C2C12 + DIO-GFP, n = 3; C2C12 + DIO-HIF2α TM, n = 3). **(B)** EPO protein levels in the medium of C2C12 myotube transduced with AAV-DIO-HIF2α TM (C2C12 + DIO-GFP, n = 3; C2C12 + DIO-HIF2α TM, n = 3). **(C)** Open field test results of skm-HIF2α

mice (control, n = 4; skm-HIF2α, n = 4). (**D**) Rectal temperature was reduced in skm-HIF2α mice (control, n = 6; skm-HIF2α, n = 8). **(E)** Infra-red imaging of the skm-HIF2α mice. Data are shown as the mean ± SEM. Unpaired two-tailed Student's t-test **(A, B, D)** or two-way ANOVA with Bonferroni's post-hoc test (**C**) was used for statistical analysis. $^{**}P < 0.01$, $^{***}P < 0.001$.

Supplemental Figure 14. HIF2α-dependent EPO production in skeletal muscle promotes erythropoiesis.

(A, G) Hematocrit test of (**A**) male or (**G**) female PHD mTKO mice (PHD $1/2/3^{fl/fl}$, n = 5; PHD mTKO, n = 5). **(B)** Representative image of capillary tubes showing the hematocrit levels in blood samples from PHD mTKO male mice (PHD $1/2/3^{fl/fl}$, n = 5; PHD mTKO, n = 5). **(C)** Representative image of ballooned heart and spleen of PHD mTKO mouse**.** **(D, H)** Survival ratio of **(D)** male or (**H**) female PHD mTKO mice (PHD $1/2/3^{fl/fl}$, n = 9–18; PHD mTKO, n = 7–14). **(E)** *Epo* mRNA expression levels in indicated tissues (PHD $1/2/3^{fl/fl}$, n = 4–6; PHD mTKO, n = 4–6) **(F)** *Epo* mRNA levels in the soleus of PHD single mKO mice (PHD1$^{fl/fl}$, n = 4; PHD1 mKO, n = 5; PHD2$^{fl/fl}$, n = 4; PHD2 mKO, n = 4; PHD3$^{fl/fl}$, n = 4; PHD3 mKO, n = 7). **(I)** Survival ratio of male skm-HIF1α mice (control, n = 9; skm-HIF1α, n = 9). **(J)** Representative image of the heart and spleen of skm-HIF1α mouse. **(K)** Hematologic parameters of skm-HIF1α mice (control, n = 6; skm-HIF1α, n = 4). **(L)** Hematologic parameters of mice (control, n = 5; PHD mTKO Cas9 + sgLacZ, n = 3; PHD mTKO Cas9 + sgEPO, n = 3). **(M)** Body weights of male PHD mTKO Cas9 + sgEPO mice on an HFD (control, n = 5; PHD mTKO Cas9 + sgLacZ, n = 3; PHD mTKO Cas9 + sgEPO, n = 3). **(N)** Glucose tolerance test (left) of PHD mTKO Cas9 + sgEPO mice and area under curve (right) results (control, n = 5; PHD mTKO Cas9 + sgLacZ, n = 3; PHD mTKO Cas9 + sgEPO, n = 3). Data are shown as

the mean ± SEM. Unpaired two-tailed Student's t-test **(A, E, F, G, K)** or one-way (**L**, **N**) and repeated measured two-way ANOVA (**M, N**) with Bonferroni's post-hoc test was used for statistical analysis. **$P < 0.01$, ***$P < 0.001$.

Supplemental Figure 15. Myofiber HIF2α is dispensable for EPO induction under physiological stress.

(A) Hematocrit test two days after phlebotomy in C57BL/6 mice (control, n = 5; Phlebotomy, n = 5). (**B**) Immunoblot (left) of HIF2α protein and quantification (right) in the soleus of mice after phlebotomy (control, n = 4; Phlebotomy, n = 4). (**C**) mRNA levels of *Epo* in the soleus after phlebotomy (control, n = 5; Phlebotomy, n = 5). (**D**) mRNA levels of *Epas1* (HIF2α encoding gene) in the soleus of HIF2α mKO mice (control, n = 4; HIF2α mKO, n = 4) **(E)** Hematocrit test in the HIF2α mKO mice (control, n = 4; HIF2α mKO, n = 4). **(F)** mRNA levels of *Epo* in the soleus of HIF2α mKO mice (control, n = 5; HIF2α mKO, n = 4). **(G)** Serum EPO protein level in HIF2α mKO mice after phlebotomy (control, n = 5; HIF2α mKO, n = 4; control + Phlebotomy, n = 5; HIF2α mKO + Phlebotomy, n = 4). Data are shown as the mean ± SEM. Unpaired two-tailed Student's t-test **(A, B, C, D, E, F)** or one-way ANOVA with Bonferroni's post-hoc test (**G**) was used for statistical analysis. *$P < 0.05$, **$P < 0.01$, ***$P < 0.001$, ****$P < 0.0001$.

Supplemental Table 1. gRNA sequence for *Epo* knockout

Name	Sequence
sgEPO	GCGACAGTCGAGTTCTGGAG

Supplemental Table 2. Secondary antibodies for immunofluorescence analysis

Name	Company	Catalogue number
DyLight™ 405 AffiniPure Fab Fragment Goat Anti-Mouse IgG2b, Fcγ fragment specific	Jackson ImmunoResearch	115-477-187
Alexa Fluor® 488 AffiniPure Fab Fragment Goat Anti-Mouse IgG1, Fcγ fragment specific	Jackson ImmunoResearch	115-547-185
Alexa Fluor® 594 AffiniPure Fab Fragment Goat Anti-Mouse IgM, μ chain specific	Jackson ImmunoResearch	115-587-020
Donkey anti-Rabbit IgG (H+L) Highly Cross-Adsrobed Secondary Antibody, Alexa Fluor™ 647	Thermofisher Scientific	A-31573

Supplemental Table 3. Sequences for primers (forward, For; reverse, Rev)

Gene	Sequences for primers	
Acadl	For	TCTTTTCCTCGGAGCATGACA
	Rev	GACCTCTCTACTCACTTCTCCAG
Acadm	For	AGGGTTTAGTTTTGAGTTGACGG
	Rev	CCCCGCTTTTGTCATATTCCG
Bnip3	For	TCCTGGGTAGAACTGCACTTC
	Rev	GCTGGGCATCCAACAGTATTT
Ccnd1	For	GCGTACCCTGACACCAATCTC
	Rev	ACTTGAAGTAAGATACGGAGGGC
Cox1	For	GCTAGCCGCAGGCATTACTA
	Rev	CTCCTCCAGCGGGATCAAAG
Cox2	For	ACCTGGTGAACTACGACTGC
	Rev	GGACTGCTCATGAGTGGAGG
Cox3a	For	GCAGGATTCTTCTGAGCGTTCT
	Rev	GTCAGCAGCCTCCTAGATCATGT
Cox4	For	ACCAAGCGAATGCTGGACAT
	Rev	GGCGGAGAAGCCCTGAA
Cox7a1	For	CAGCTTGTAATGGGTTCCACAGT
	Rev	GTACTGGGAGGTCATTGTCGG
Cox8b	For	GAACCATGAAGCCAACGACT
	Rev	GCGAAGTTCACAGTGGTTCC
Epas1	For	CTGGACAAAGCCTCCATCATG
	Rev	CCGACAGAAAGATCAT GTCACC
Epo	For	ACAAAGCCATCAGTGGTCTACG
	Rev	TCTGGAGGCGACATCAATTCC
Fbxo32	For	ATGCACACTGGTGCAGAGAG
	Rev	TGTAAGCACACAGGCAGGTC
Glut1	For	TCAAACATGGAACCACCGCTA
	Rev	AAGAGGCCGACAGAGAAGGAA
Ldh	For	GGAAGGAGGTTCACAAGCAG
	Rev	TCACAACATCCGAGATTCCA
Murf-1	For	GTGTGAGGTGCCTACTTGCTC
	Rev	GCTCAGTCTTCTGTCCTTGGA
Myh1	For	CTCTTCCCGCTTTGGTAAGTT
	Rev	CAGGAGCATTTCGATTAGATCCG
Myh4	For	AGGACCAACTGAGTGAAGTGA
	Rev	GGGAAAACTCGCCTGACTCTG

Myh7	For	ACTGTCAACACTAAGAGGGTCA
	Rev	TTGGATGATTTGATCTTCCAGGG
Nd1	For	GGGATAACAGCGCAATCCTA
	Rev	ATCGTTGAACAAACGAACCA
Ndufv1	For	TTTCTCGGCGGGTTGGTTC
	Rev	GGTTGGTAAAGATCCGGTCTTC
Serpine1	For	CAAGCTCTTCCAGACTATGGTG
	Rev	ACCTTTGGTATGCCTTTCCAC
Trim63	For	GTGTGAGGTGCCTACTTGCTC
	Rev	GCTCAGTCTTCTGTCCTTGGA
Pdk1	For	GGACTTCGGGTCAGTGAATGC
	Rev	TCCTGAGAAGATTGTCGGGGA
Pgk1	For	ATGTCGCTTTCCAACAAGCTG
	Rev	GCTCCATTGTCCAAGCAGAAT
Phd1	For	GCTGCATCACCTGTATCTAT
	Rev	TCAGACCAGAAAATGAGCAA
Phd2	For	AAAGCCATGGTTGCTTGTTA
	Rev	TACATGTCACGCATCTTCC
Phd3	For	ATGGCCGCTGTATCACCTG
	Rev	CCAGACAGTCATAGCGTACCT
Pkm2	For	TGCCGTGCTGAATGCCTGGG
	Rev	CGCCACCCGGTCAGCACAAT
Ppargc1a	For	AGCCGTGACCACTGACAACGAG
	Rev	GCTGCATGGTTCTGAGTGCTAAG
Sdha	For	GGAACACTCCAAAAACAGACCT
	Rev	CCACCACTGGGTATTGAGTAGAA
Sdhb	For	AATTTGCCATTTACCGATGGGA
	Rev	AGCATCCAACACCATAGGTCC
Tfam	For	GGAATGTGGAGCGTGCTAAAA
	Rev	ACAAGACTGATAGACGAGGGG
Tnnc1	For	GCGGTAGAACAGTTGACAGAG
	Rev	GACAAGAAACTCATCGAAGTCCA
Tnnc2	For	CCATCATCGAGGAGGTGGAC
	Rev	CTTCCCCTTCGCATCCTCTT
Tnni2	For	CGGAGGGTGCGTATGTCTG
	Rev	CAGGTCCCGTTCCTTCTCA
Vegfa	For	ACAGAAGGAGAGCAGAAGTC
	Rev	GCTTGAAGATGTACTCTATCTCG
