# Supplementary material for: Distinct HIF1α and HIF2α functions control skeletal muscle metabolism and erythropoiesis
Source: J Clin Invest. 2026 Feb 17;136(8):e195411. doi: 10.1172/JCI195411 (PMC13078885; doi:10.1172/JCI195411)

Figure. 1B

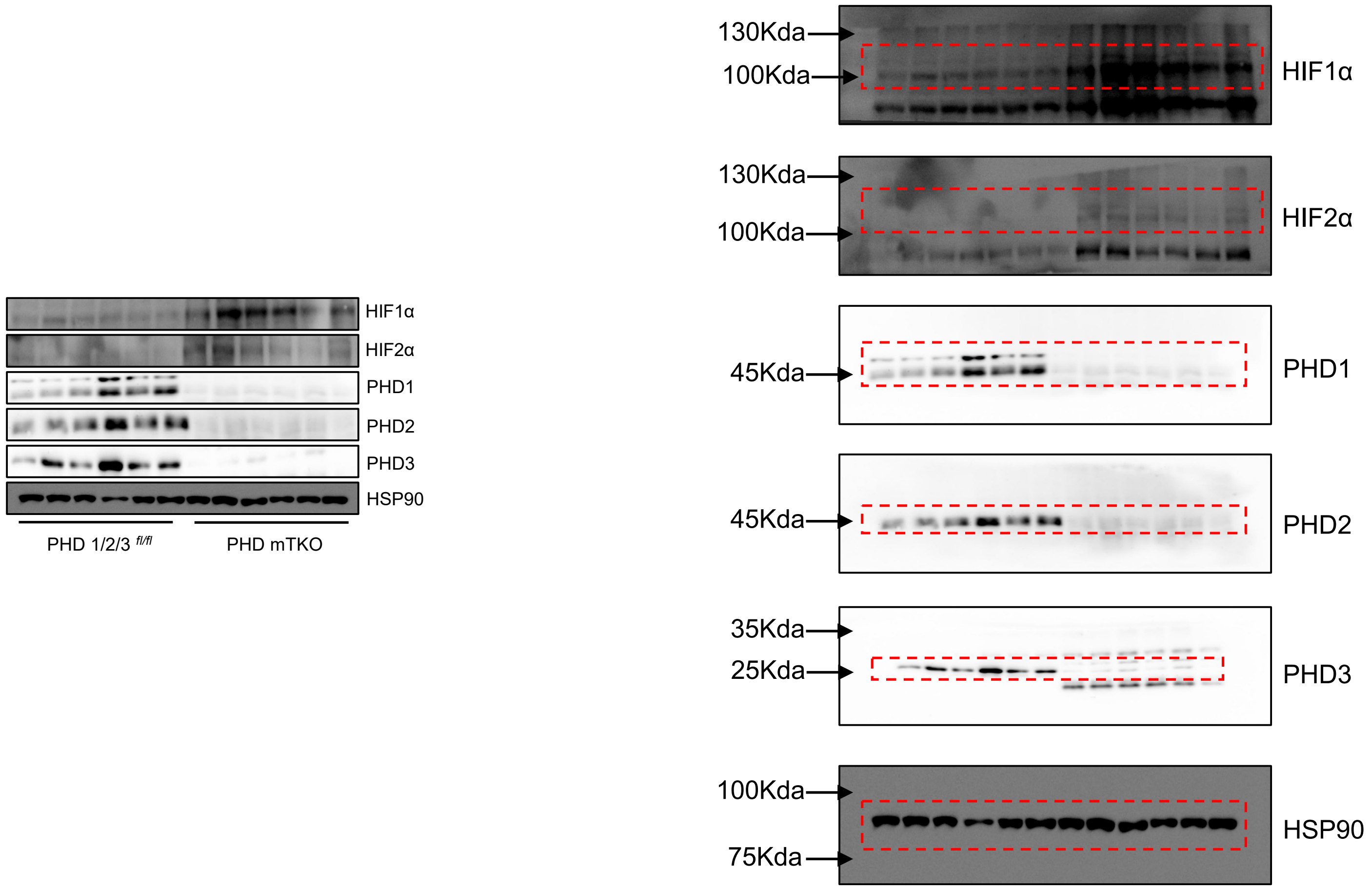
HIF1α
HIF2α
PHD1
PHD2
PHD3
HSP90
PHD 1/2/3 fl/fl
PHD mTKO
130Kda
100Kda
HIF1α
130Kda
100Kda
HIF2α
45Kda
PHD1
45Kda
PHD2
35Kda
25Kda
PHD3
100Kda
75Kda
HSP90

Figure. 1G

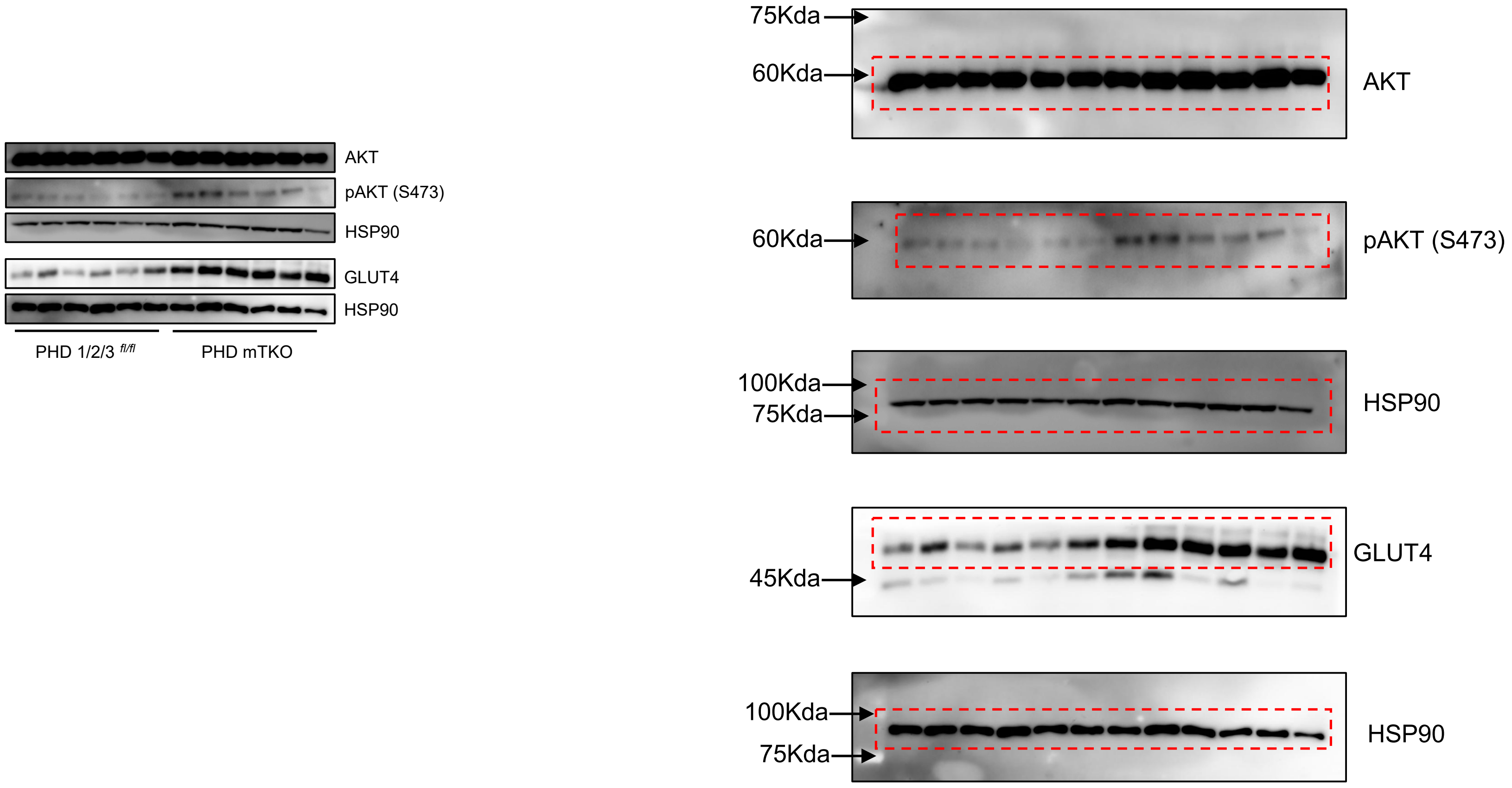
AKT
pAKT (S473)
HSP90
GLUT4
HSP90
PHD 1/2/3 fl/fl
PHD mTKO
75Kda
60Kda
AKT
60Kda
pAKT (S473)
100Kda
75Kda
HSP90
GLUT4
45Kda
100Kda
75Kda
HSP90

Figure. 1L

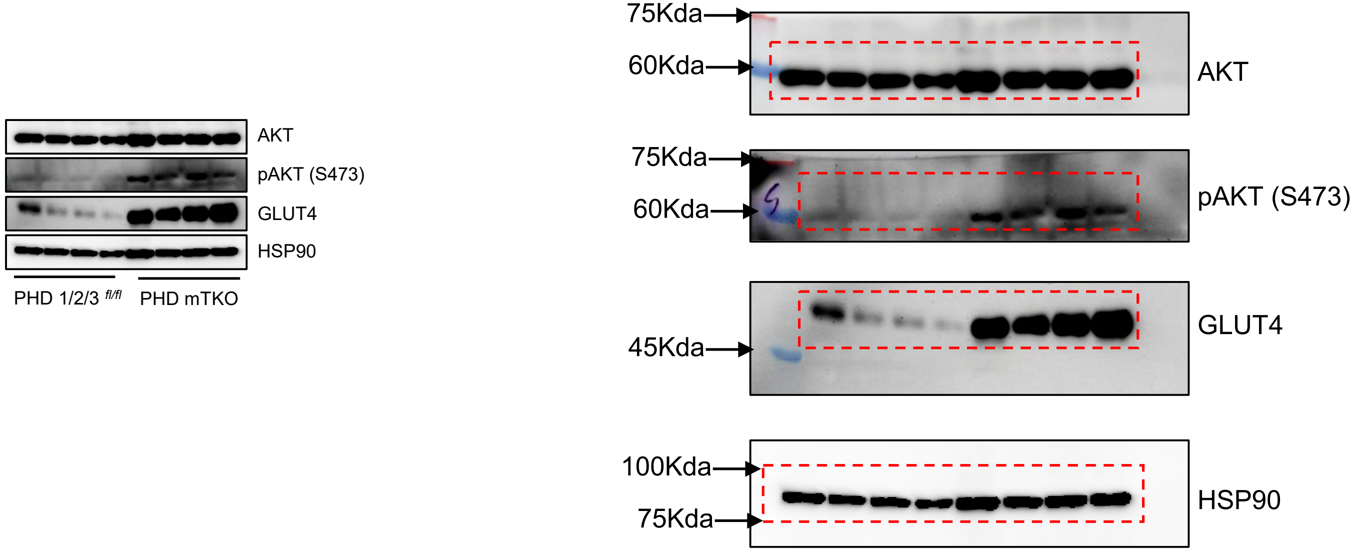
AKT
pAKT (S473)
GLUT4
HSP90
PHD 1/2/3 fl/fl
PHD mTKO
75Kda
60Kda
AKT
75Kda
60Kda
pAKT (S473)
45Kda
GLUT4
100Kda
75Kda
HSP90

Figure. 3D

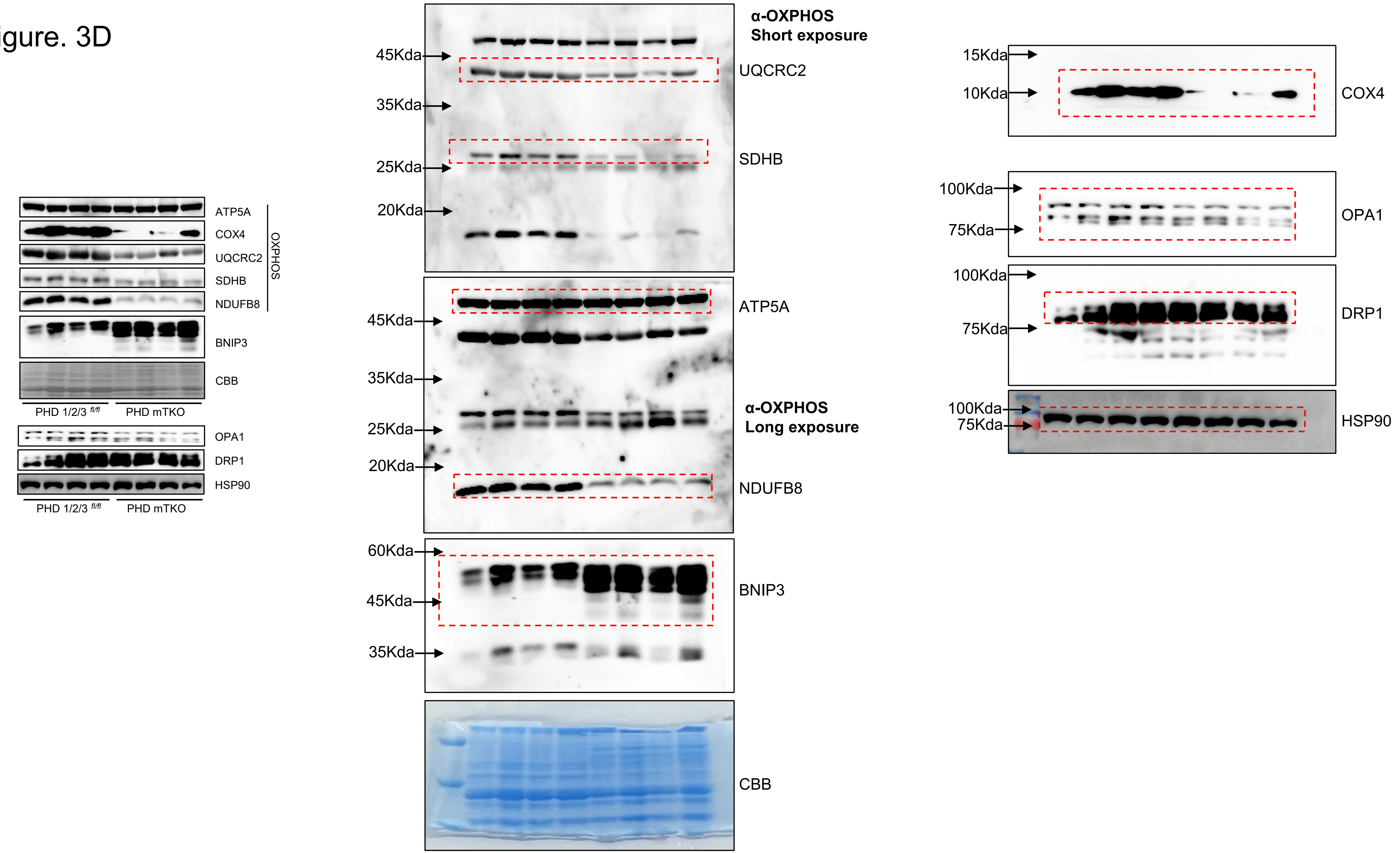

ATP5A
COX4
UQCRC2
SDHB
NDUFB8
OXPHOS
BNIP3
CBB
PHD 1/2/3 fl/fl
PHD mTKO
OPA1
DRP1
HSP90
PHD 1/2/3 fl/fl
PHD mTKO
α-OXPHOS
Short exposure
45Kda
35Kda
25Kda
20Kda
UQCRC2
SDHB
ATP5A
NDUFB8
α-OXPHOS
Long exposure
45Kda
35Kda
25Kda
20Kda
60Kda
45Kda
35Kda
BNIP3
CBB
15Kda
10Kda
COX4
100Kda
75Kda
OPA1
100Kda
75Kda
DRP1
100Kda
75Kda
HSP90

Figure. 4B

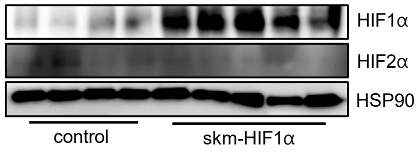

HIF1α
HIF2α
HSP90
control
skm-HIF1α

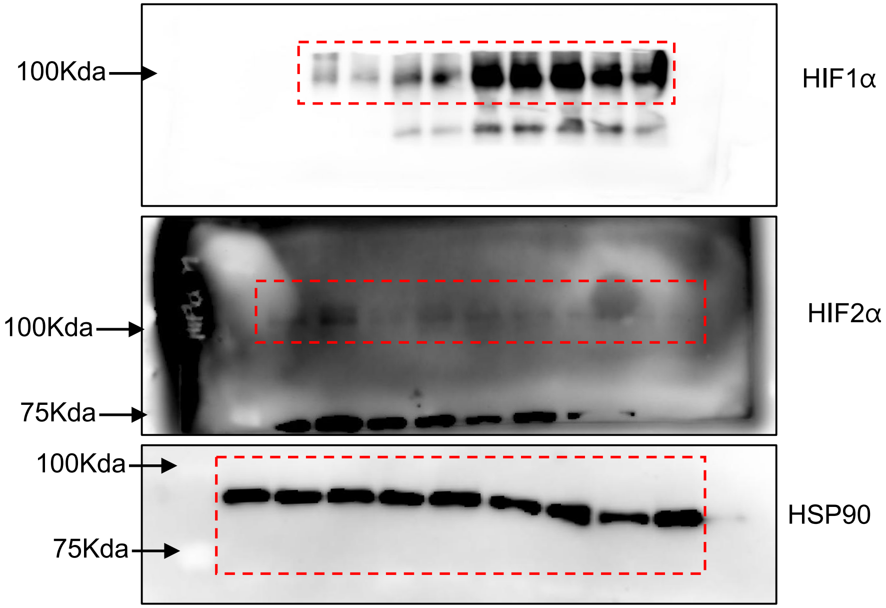

100Kda
HIF1α
100Kda
75Kda
HIF2α
100Kda
75Kda
HSP90

Figure. 4E

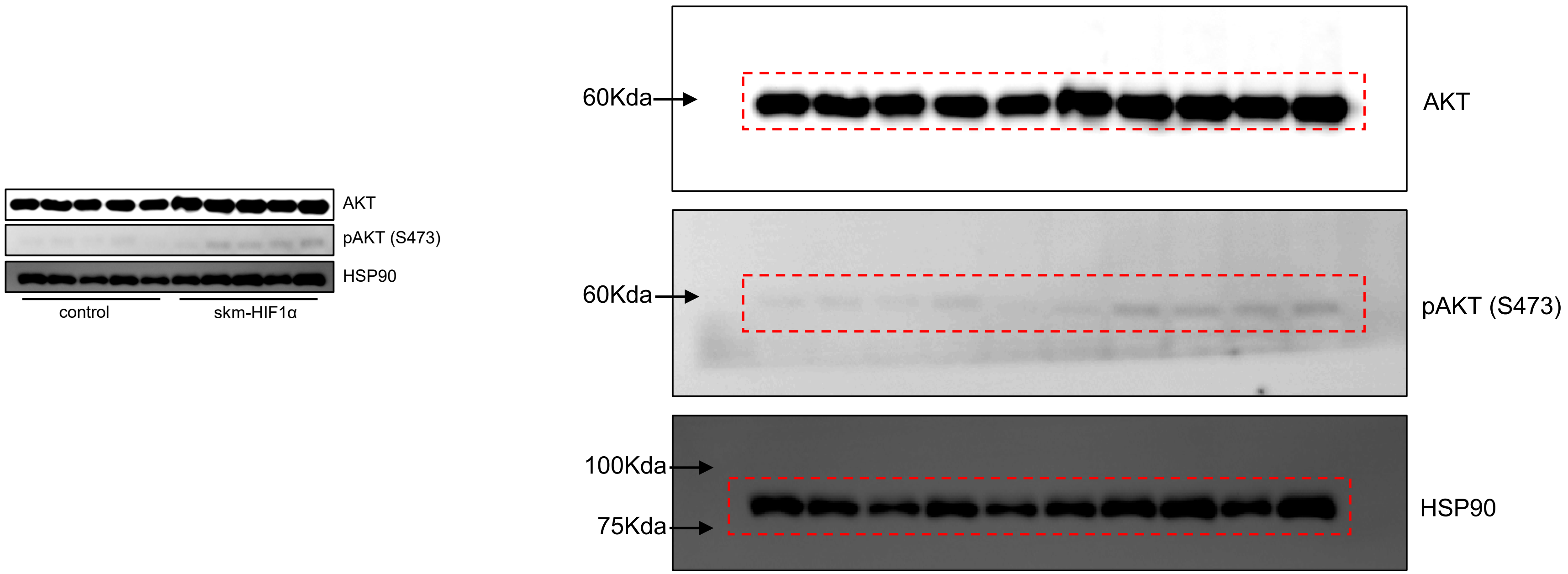

AKT
pAKT (S473)
HSP90
control
skm-HIF1α
60Kda
AKT
60Kda
pAKT (S473)
100Kda
75Kda
HSP90

Figure. 5C

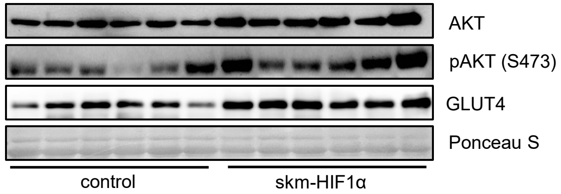
AKT
pAKT (S473)
GLUT4
Ponceau S
control
skm-HIF1α

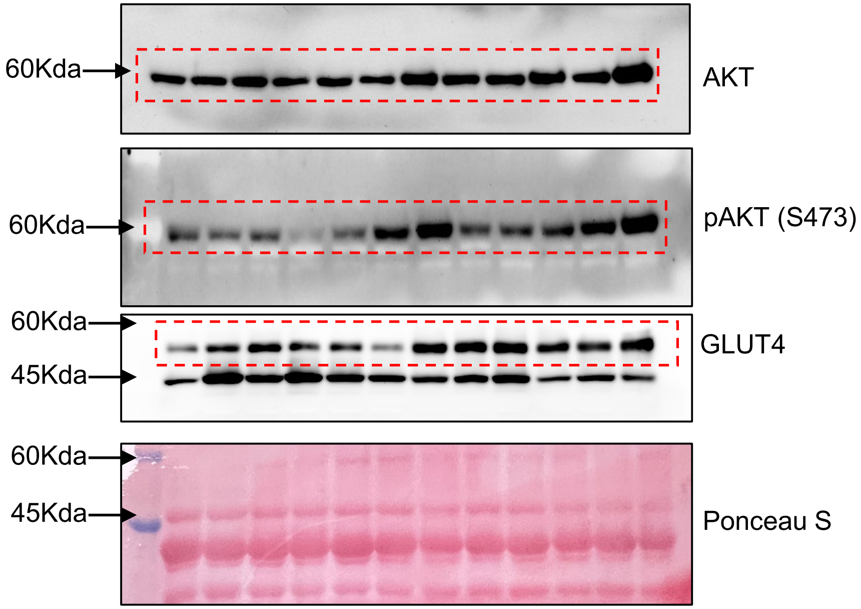
60Kda
AKT
60Kda
pAKT (S473)
60Kda
45Kda
GLUT4
60Kda
45Kda
Ponceau S

Figure. 7C

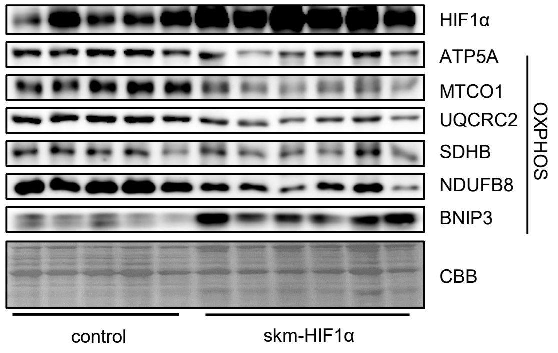
HIF1α
ATP5A
MTCO1
UQCRC2
SDHB
NDUFB8
BNIP3
CBB
OXPHOS
control
skm-HIF1α

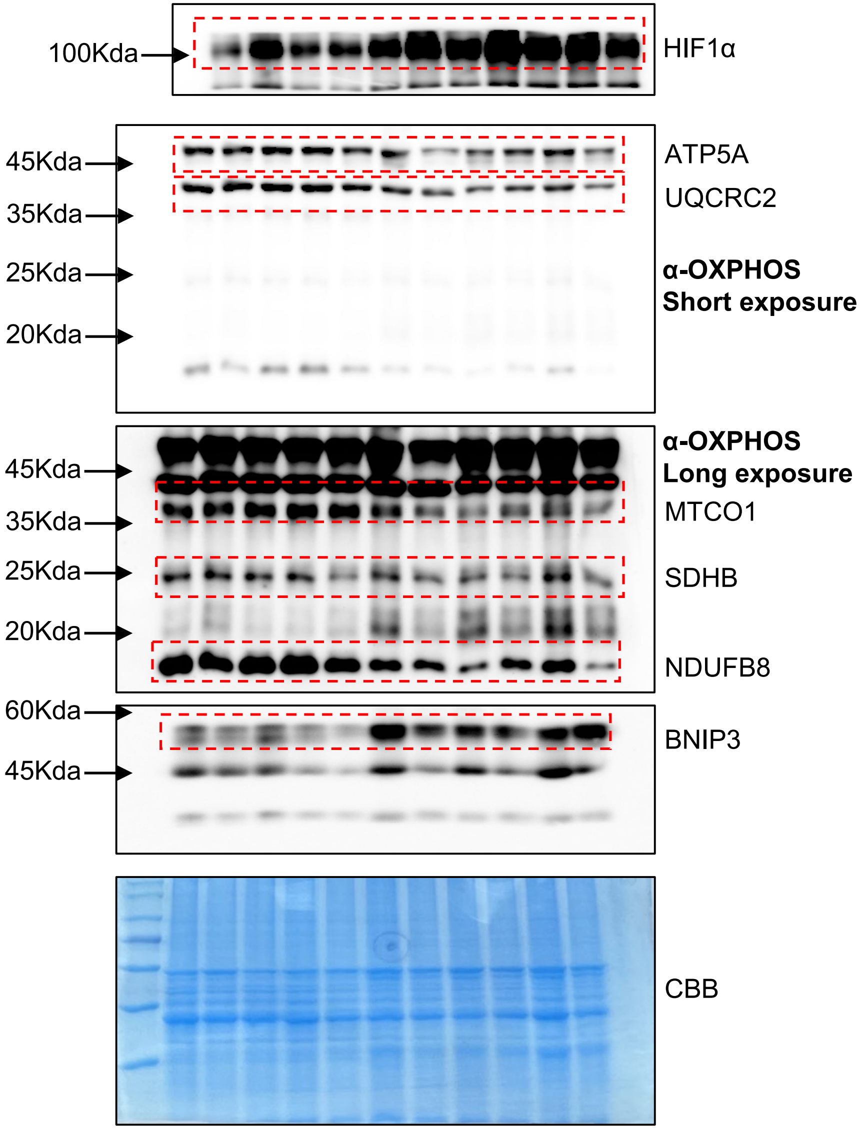
100Kda
HIF1α
45Kda
35Kda
25Kda
20Kda
ATP5A
UQCRC2
α-OXPHOS
Short exposure
α-OXPHOS
Long exposure
45Kda
35Kda
25Kda
20Kda
MTCO1
SDHB
NDUFB8
60Kda
45Kda
BNIP3
CBB

Figure. 8B

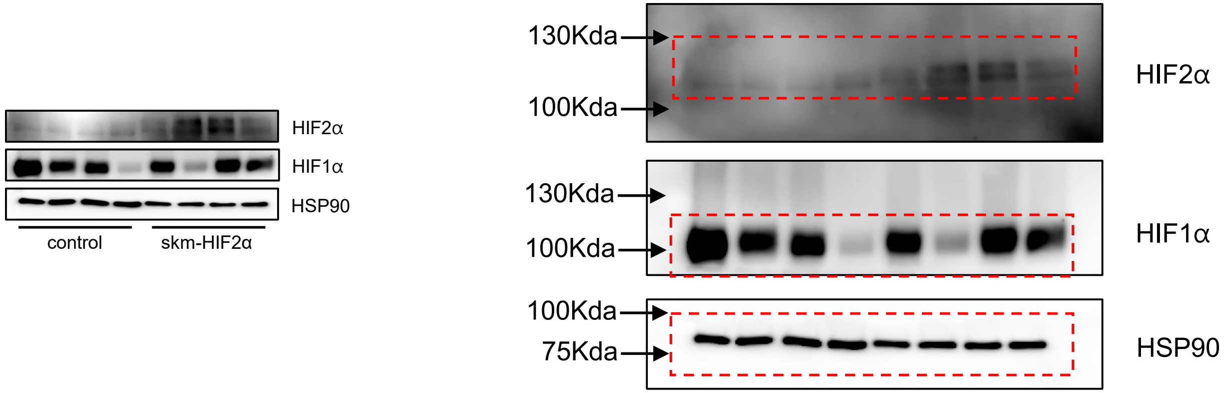
HIF2α
HIF1α
HSP90
control
skm-HIF2α
130Kda
100Kda
HIF2α
130Kda
100Kda
HIF1α
100Kda
75Kda
HSP90

Figure. 8E

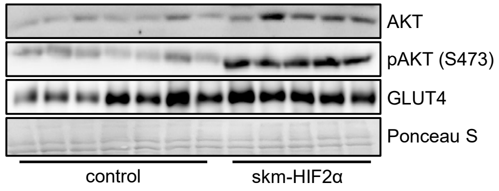
AKT
pAKT (S473)
GLUT4
Ponceau S
control
skm-HIF2α

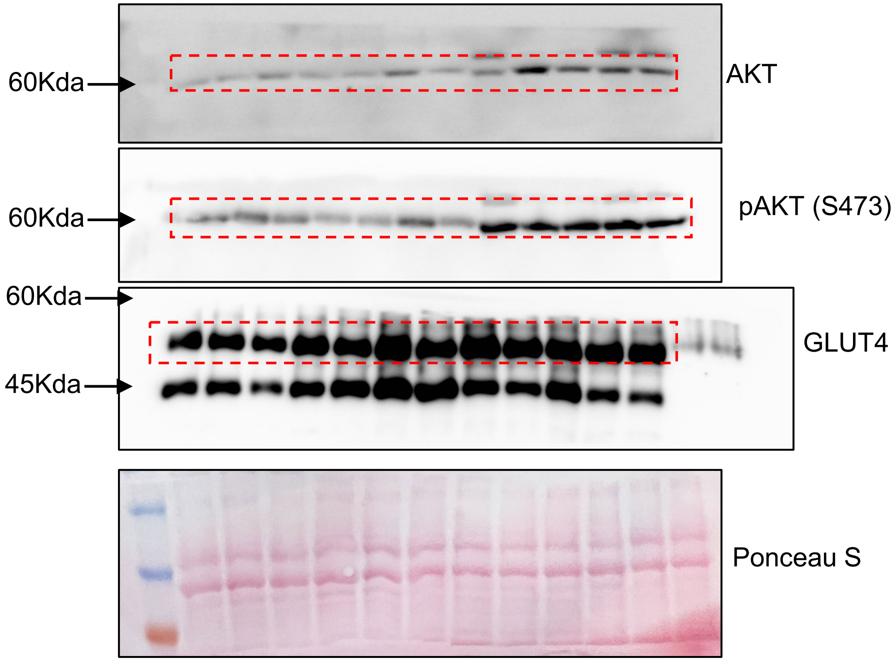
60Kda
AKT
60Kda
pAKT (S473)
60Kda
45Kda
GLUT4
Ponceau S

Supplemental Figure. 1A

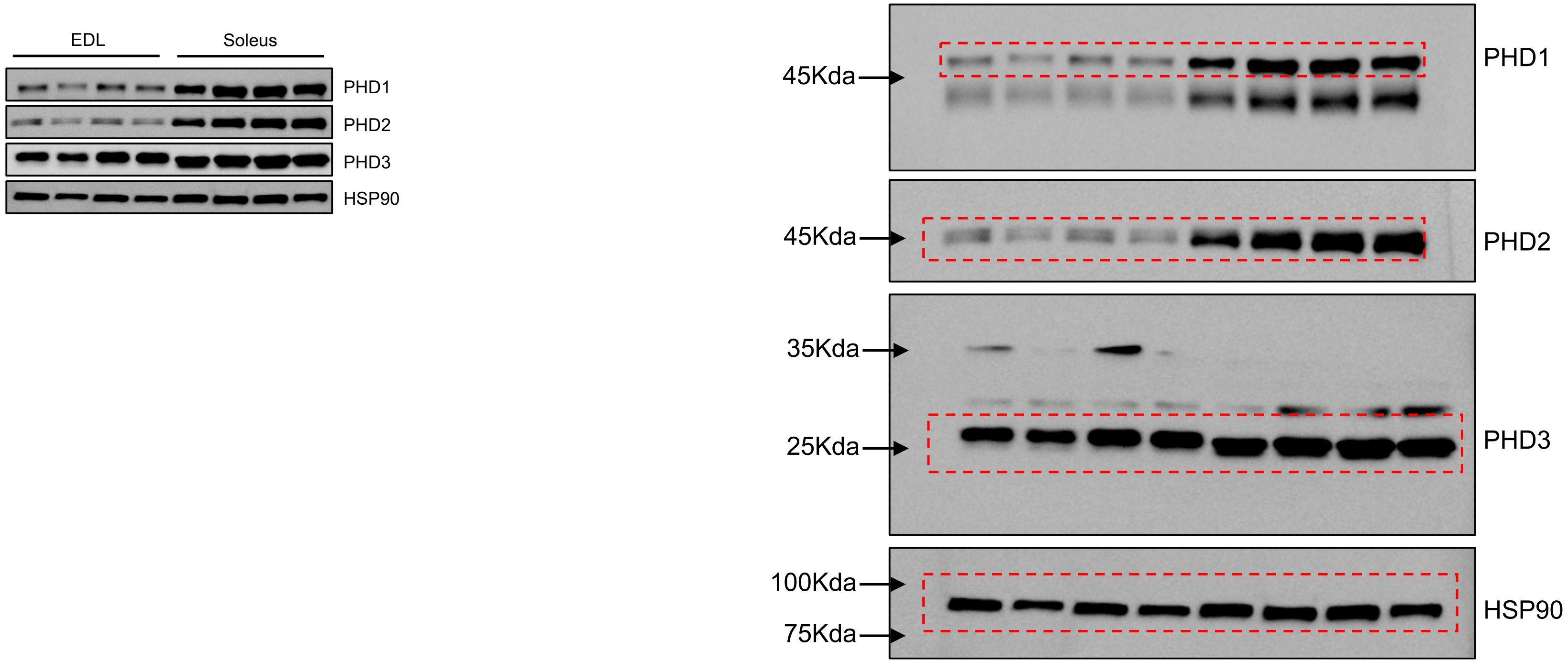

Supplemental Figure. 2A

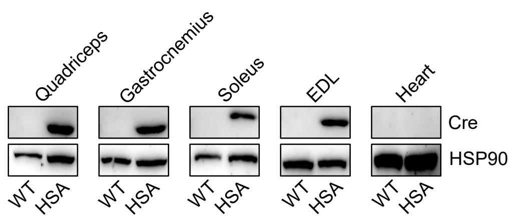

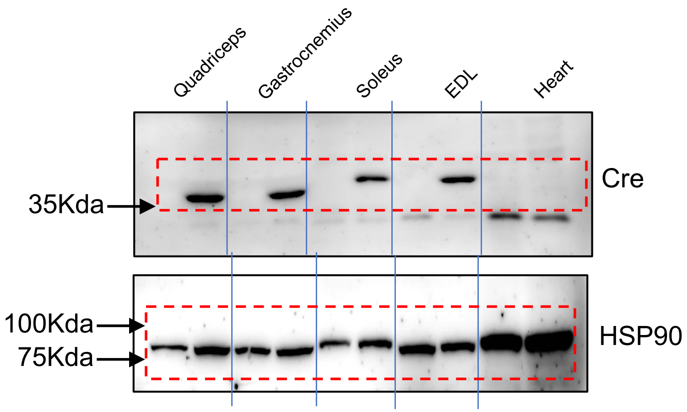

Supplemental Figure. 2D

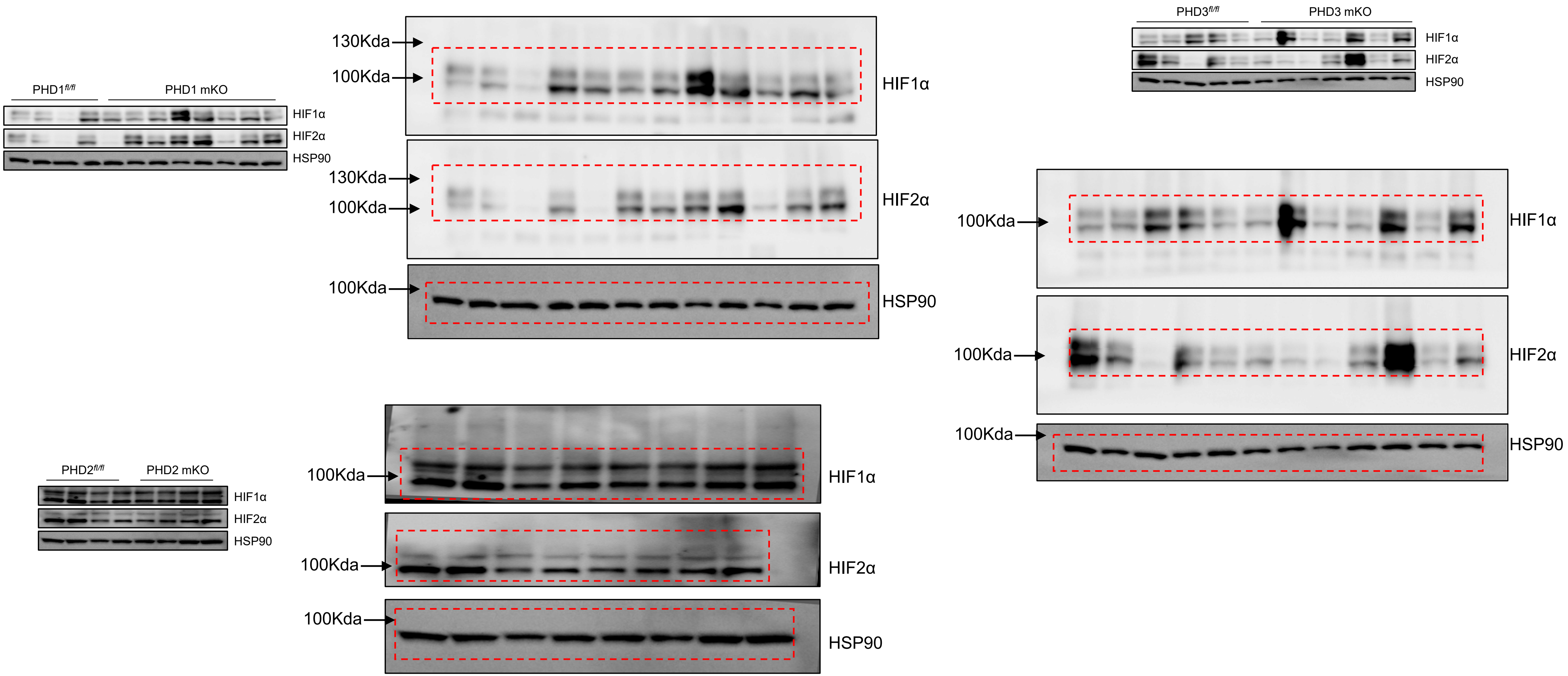

Supplemental Figure. 3A

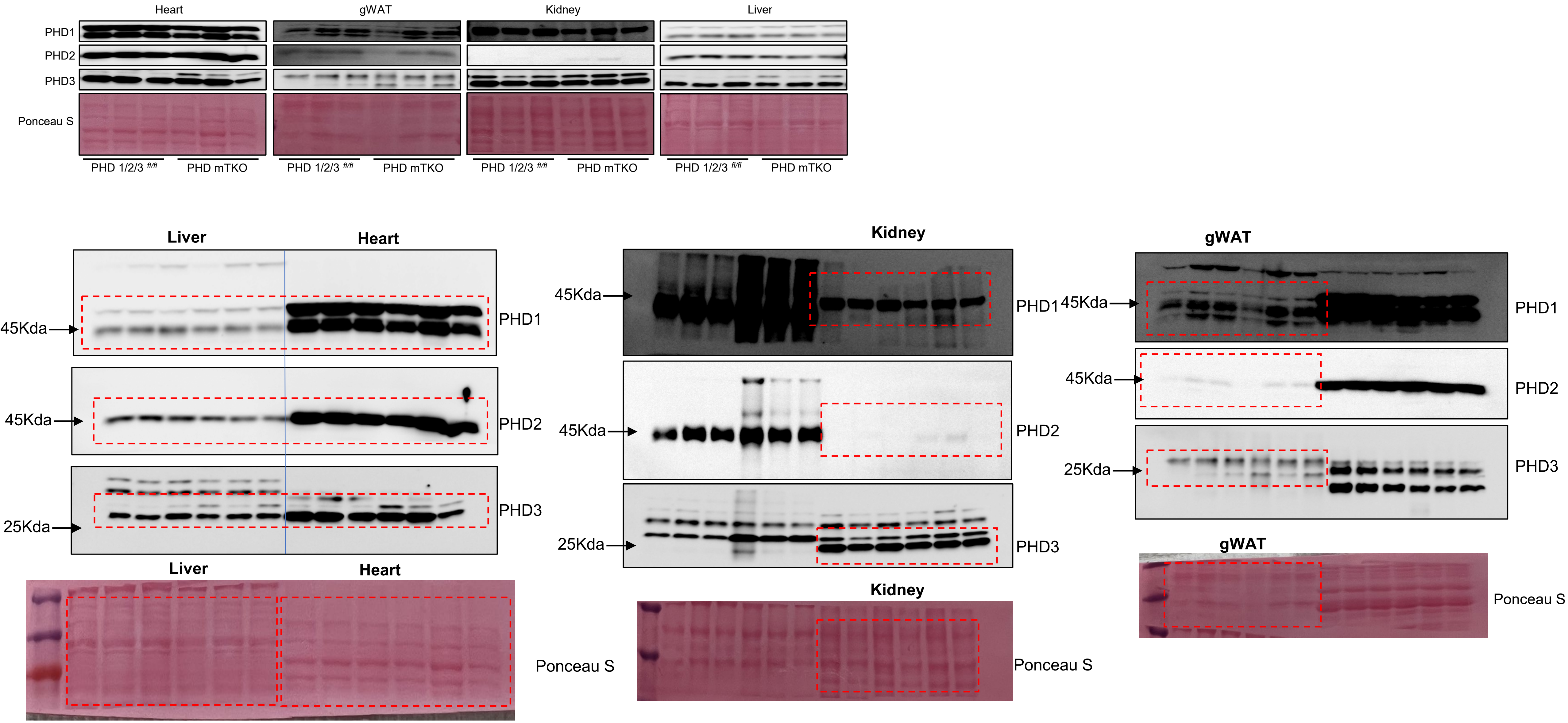

Supplemental Figure. 3C

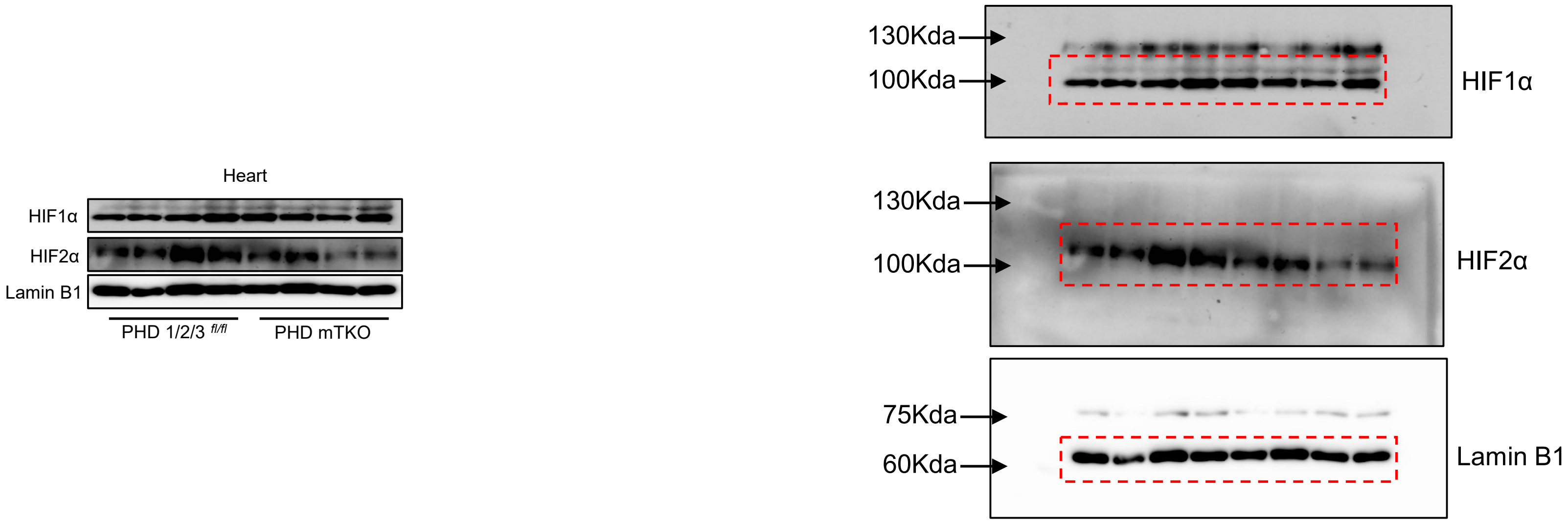

Supplemental Figure. 4E

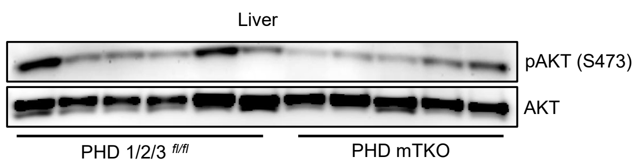

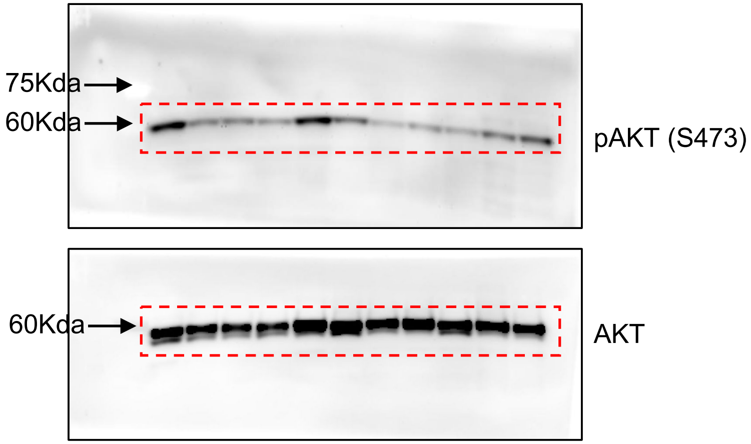

Supplemental Figure. 4F

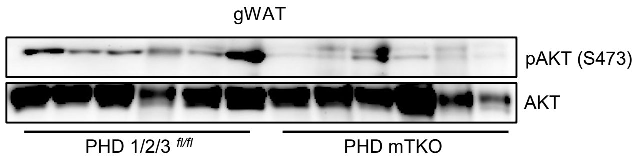

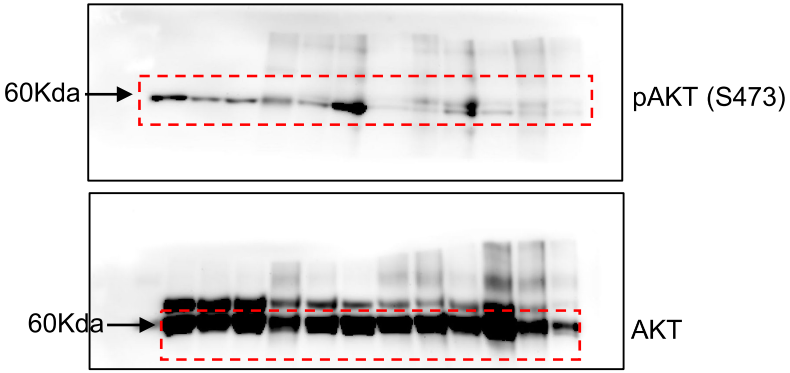

Supplemental Figure. 6A

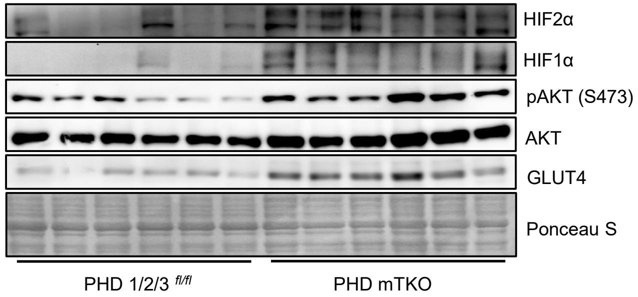

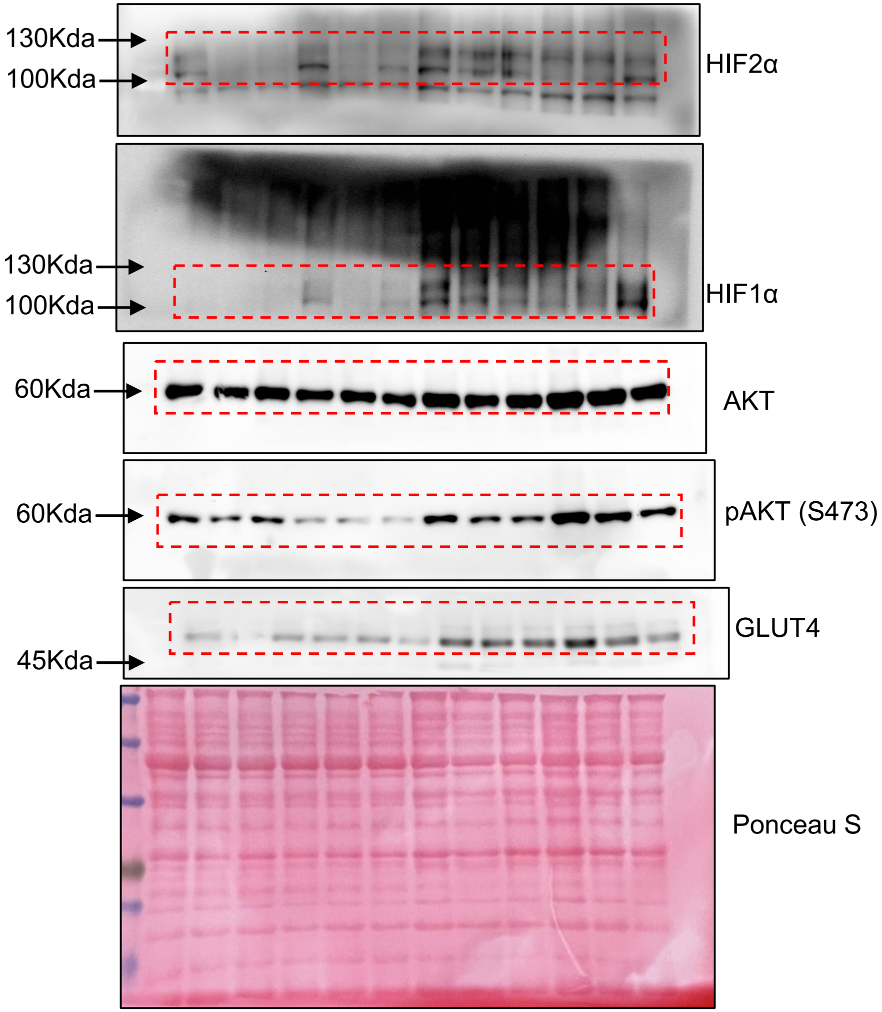

Supplemental Figure. 7A

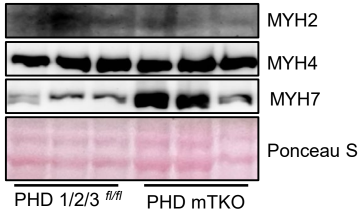

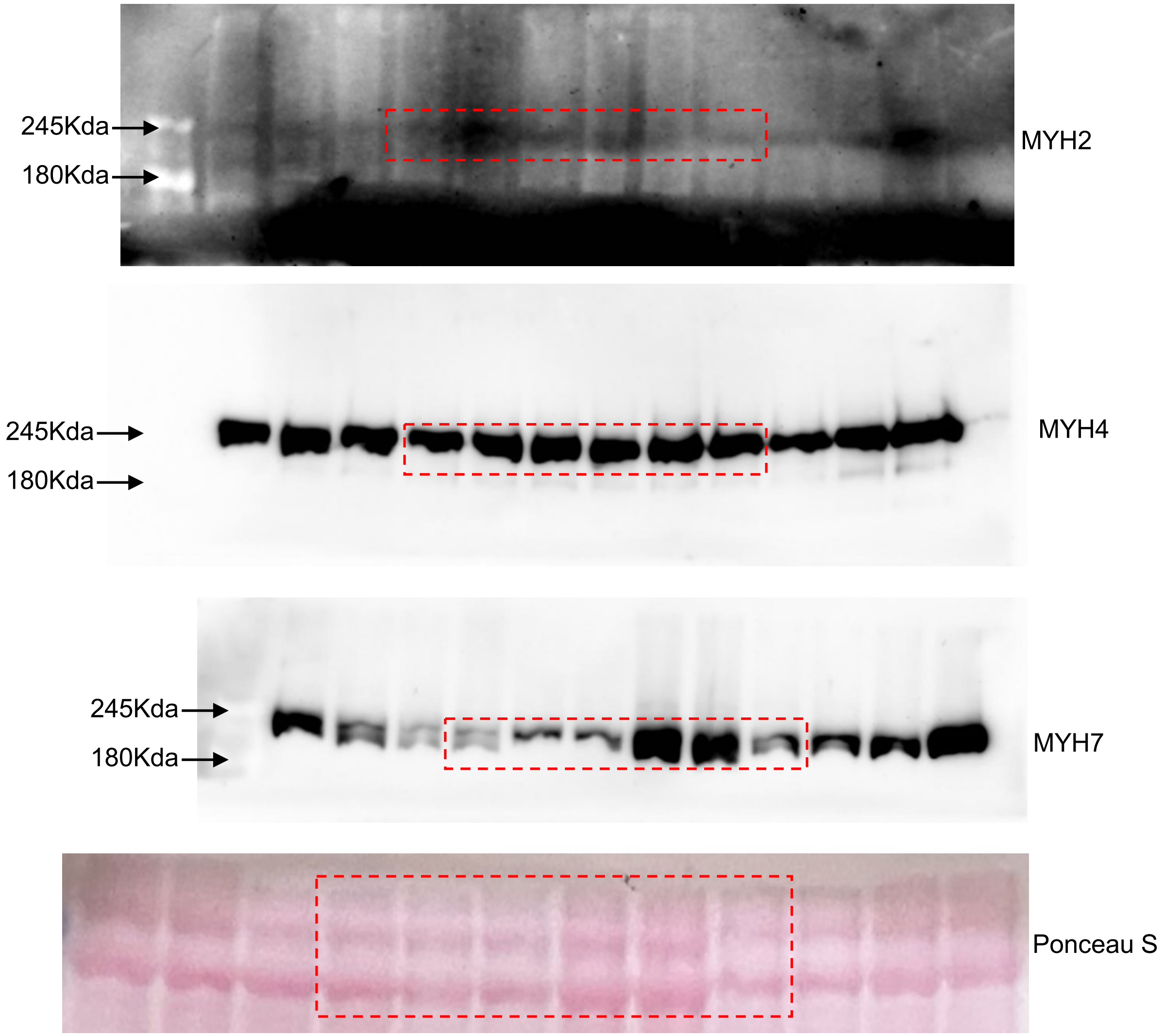

Supplemental Figure. 7G

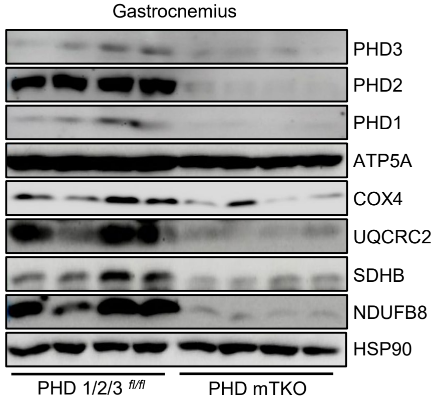

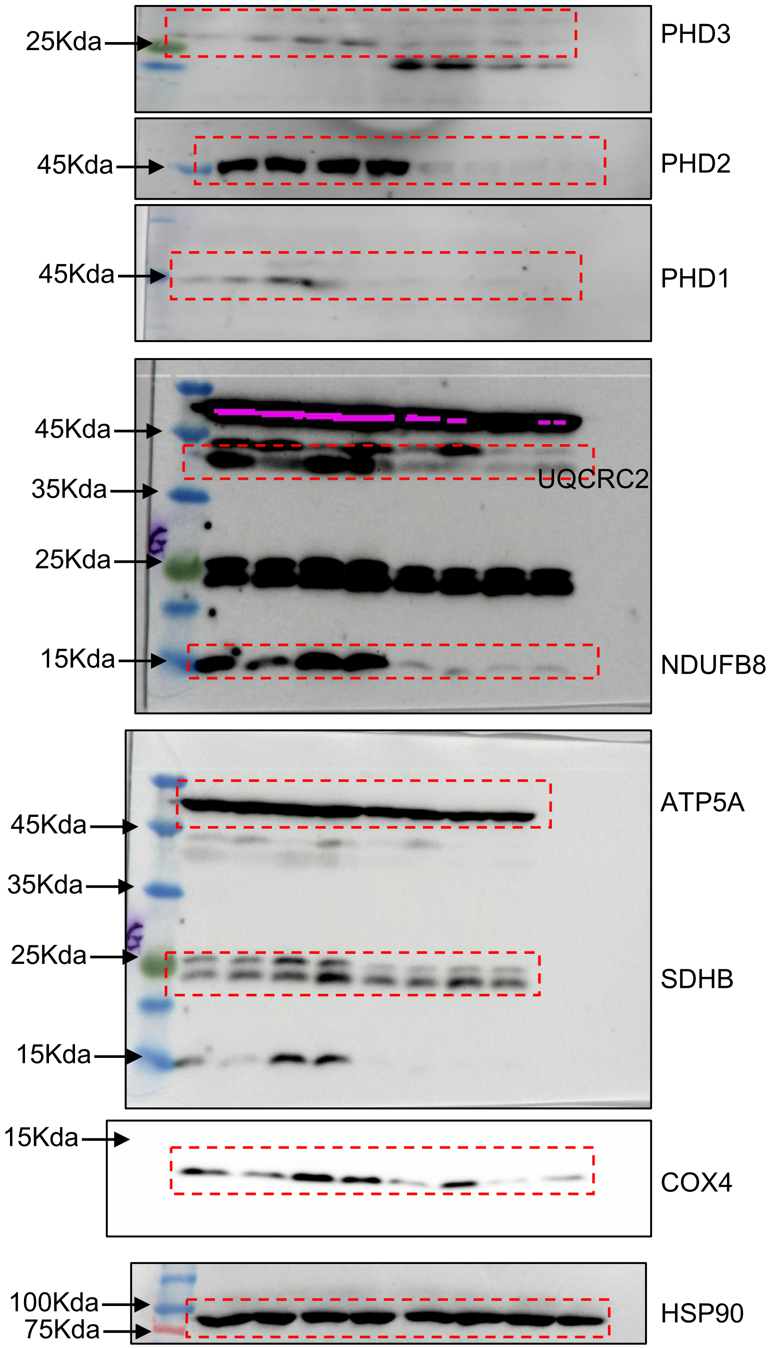

Supplemental Figure. 7H

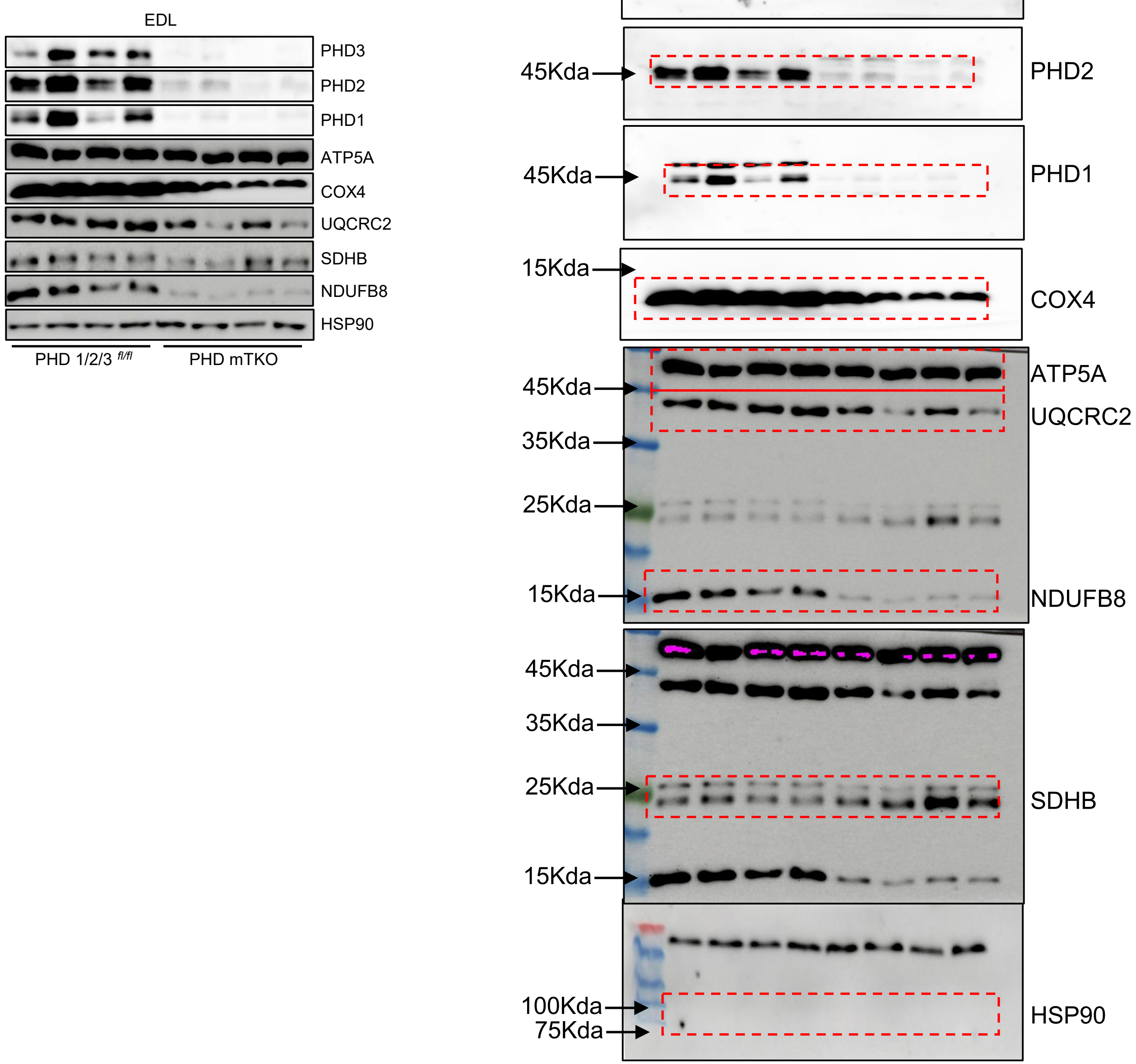

Supplemental Figure. 7I

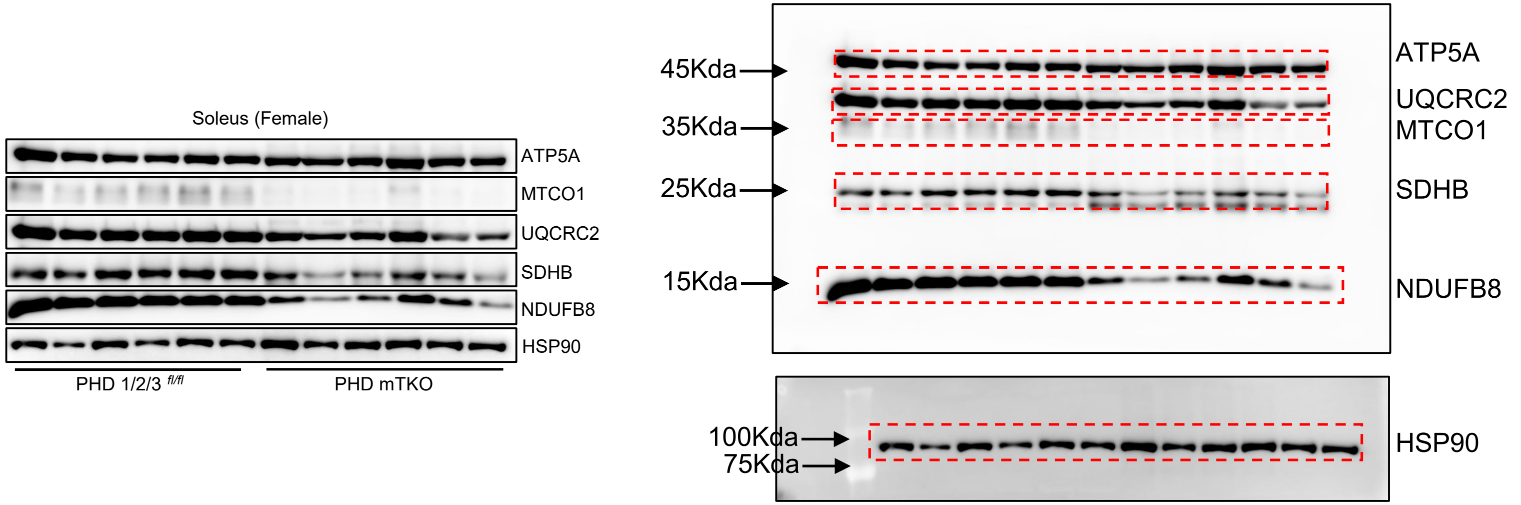

Supplemental Figure. 8A

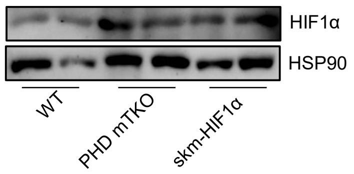

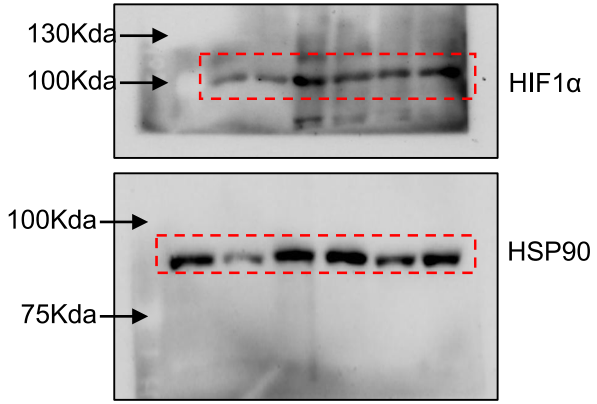

Supplemental Figure. 8B

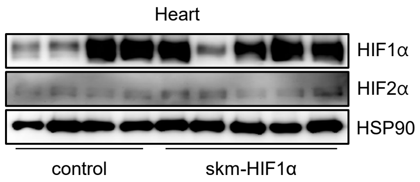

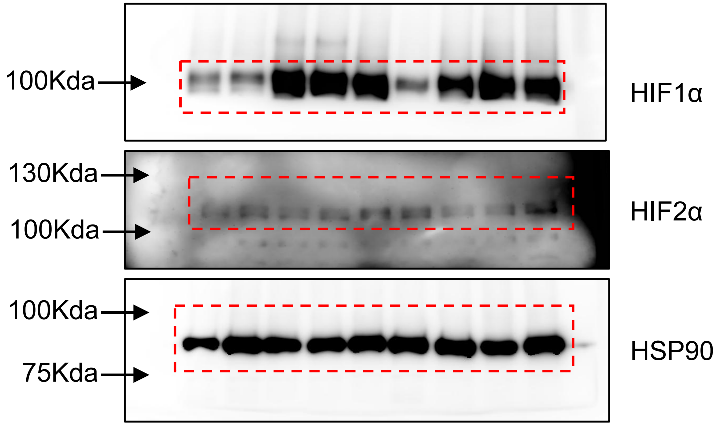

Supplemental Figure. 9A

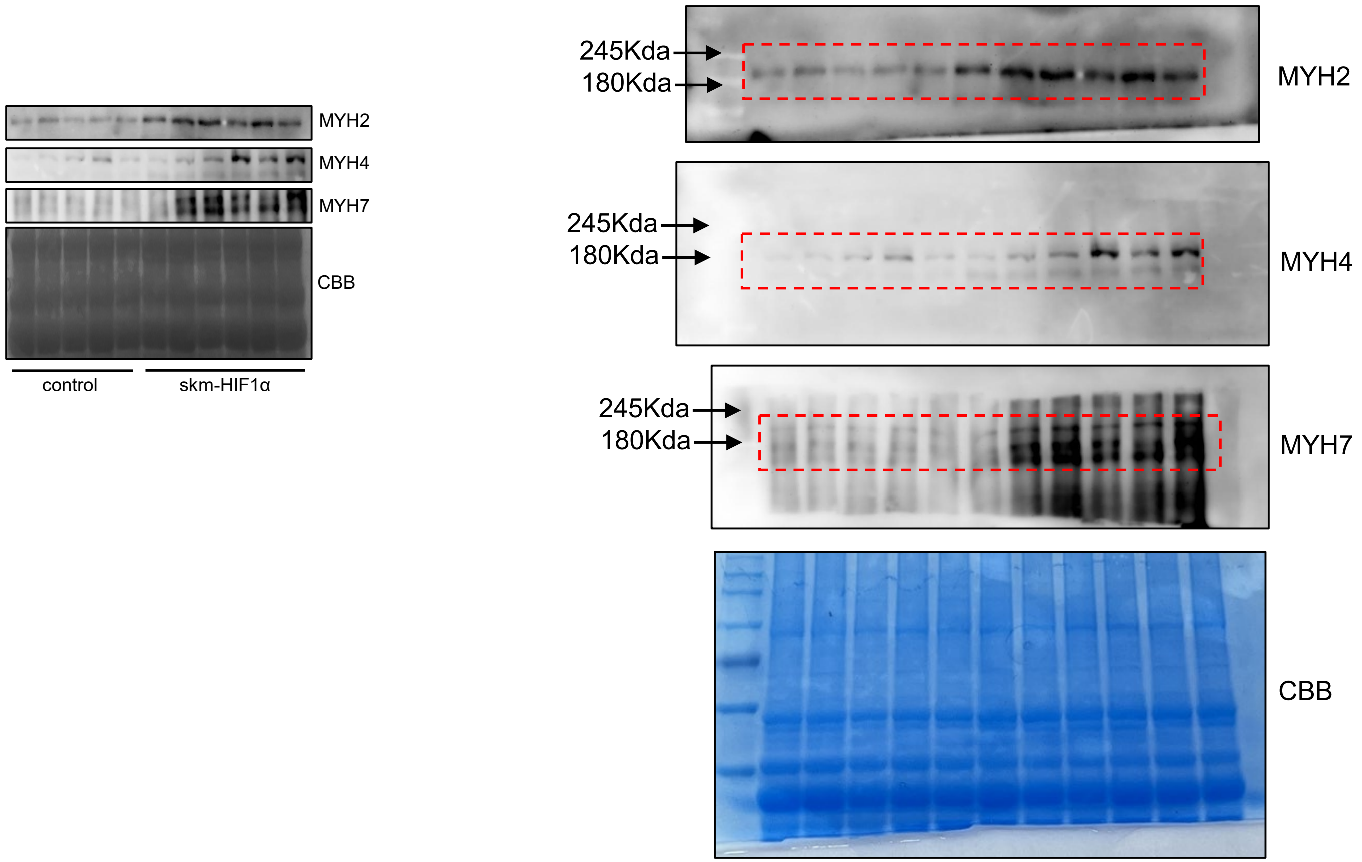

Supplemental Figure. 10E

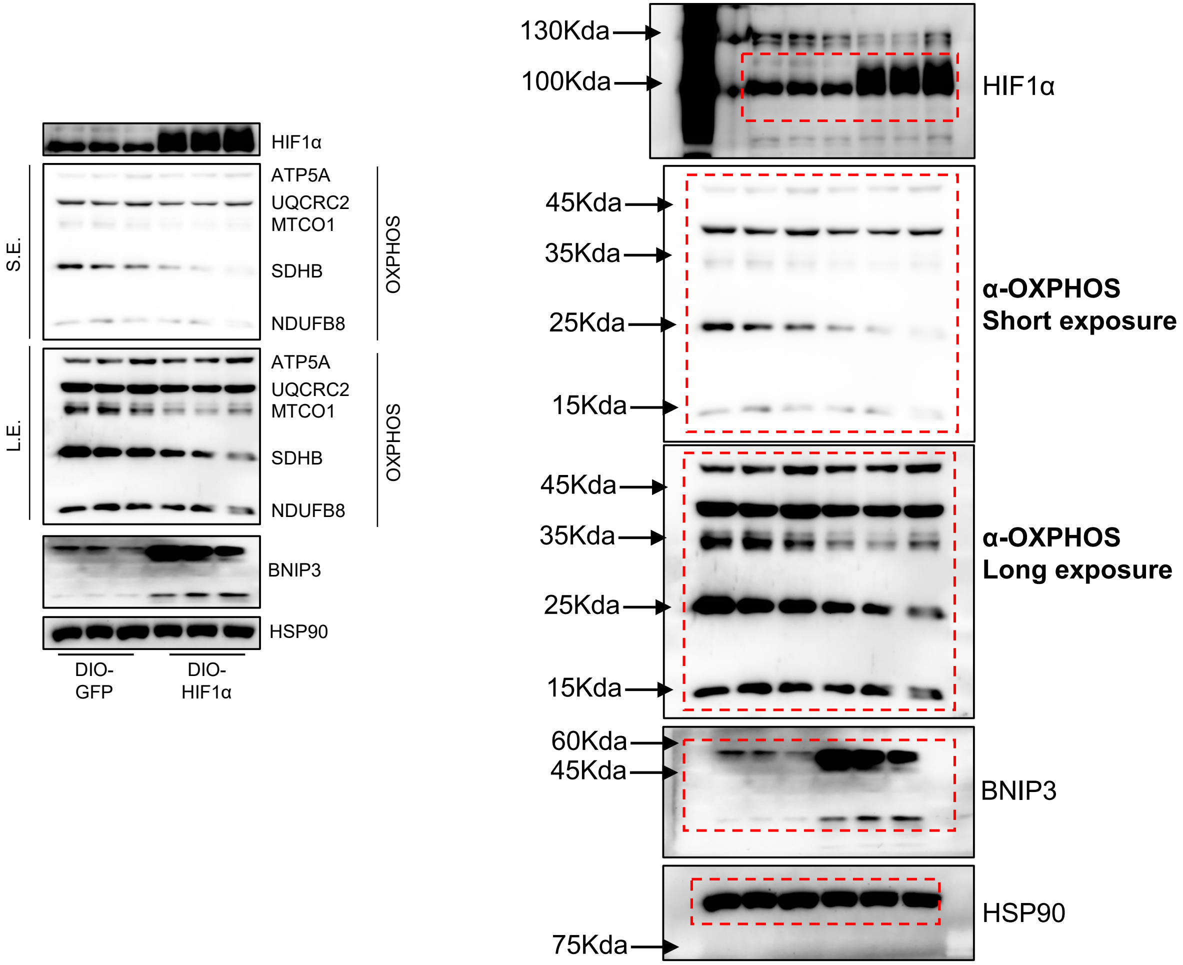

Supplemental Figure. 11A

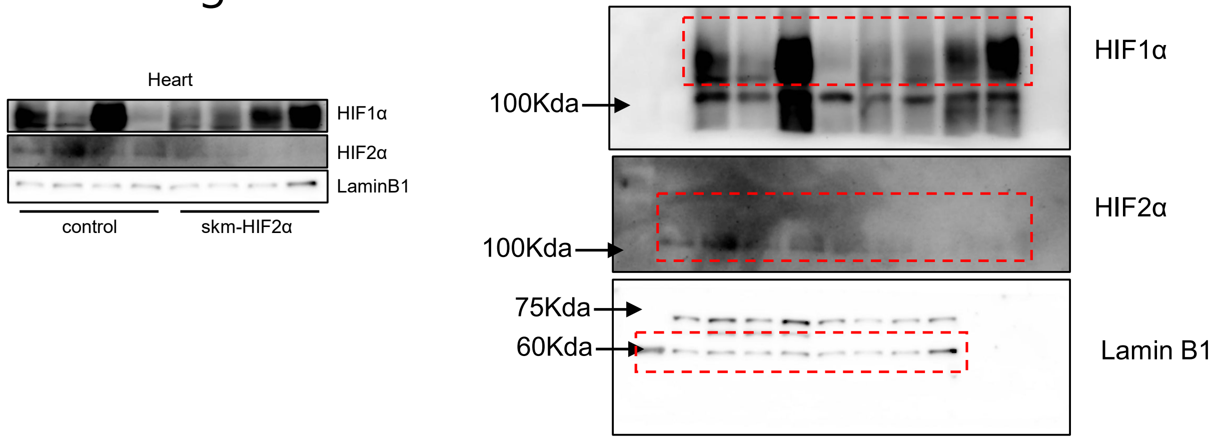

Supplemental Figure. 11B

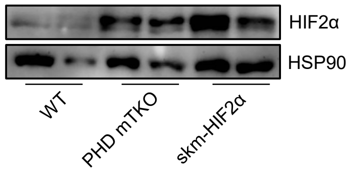

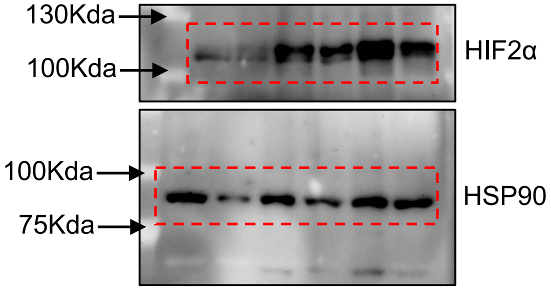

Supplemental Figure. 12A

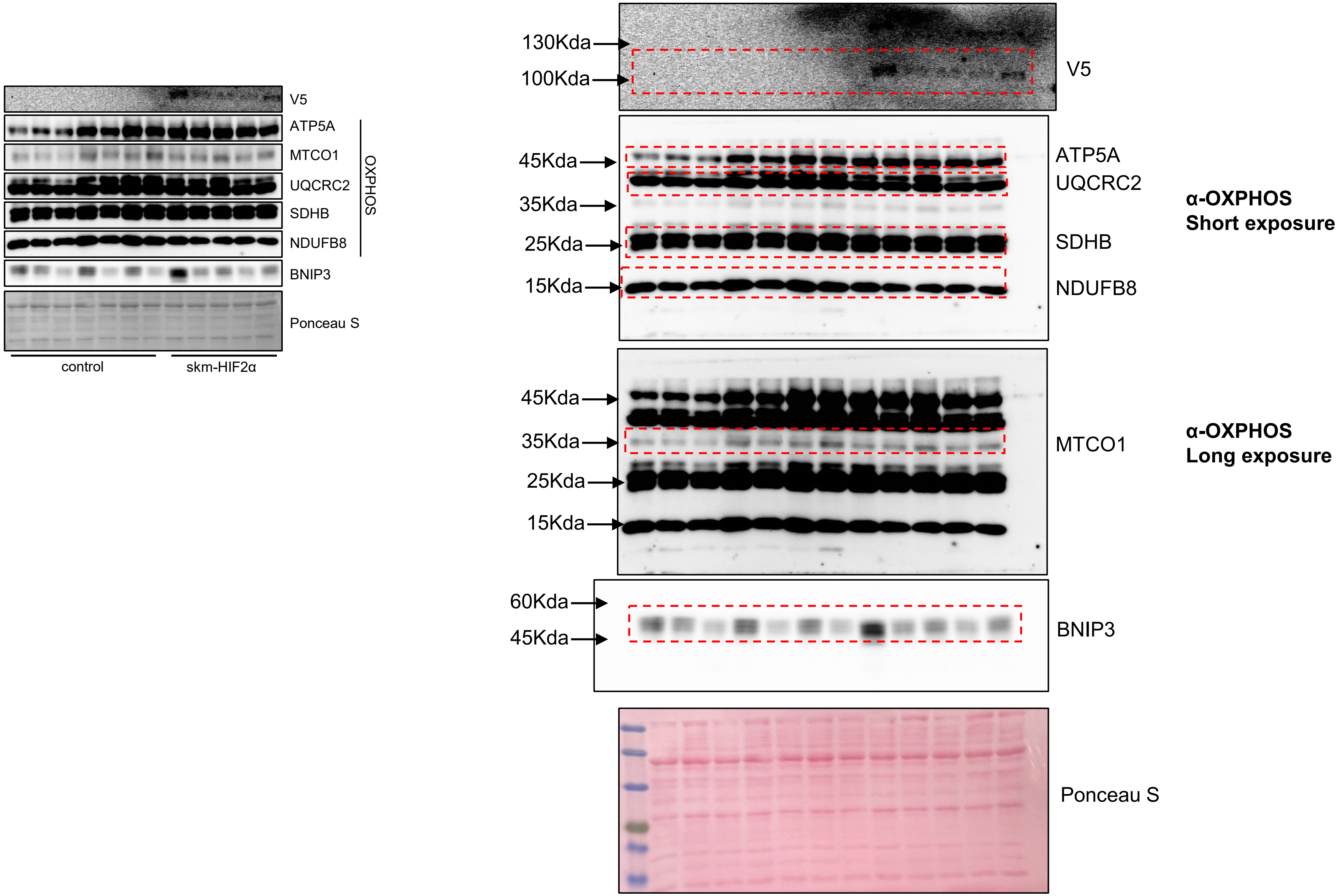

Supplemental Figure. 12B

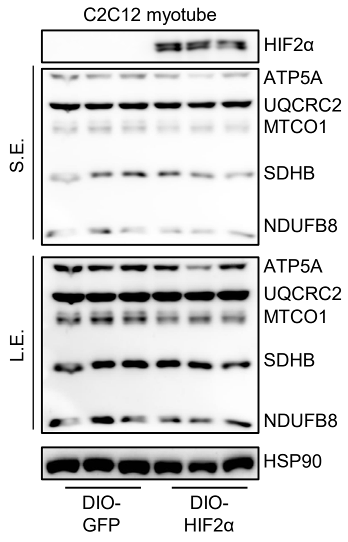

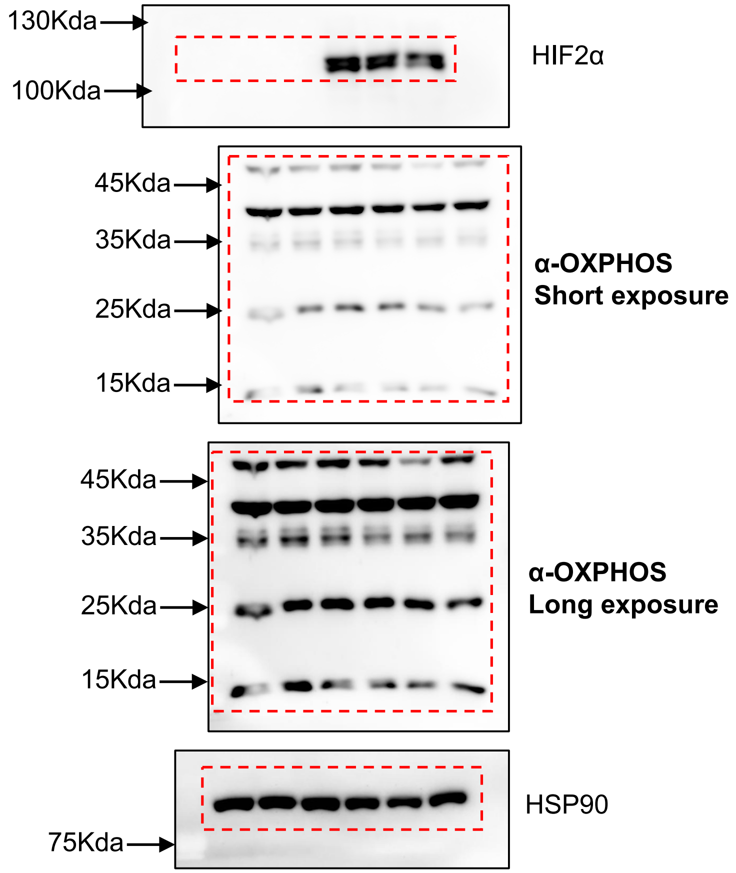

Supplemental Figure. 12D

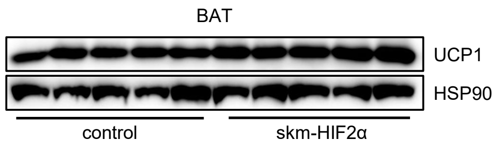

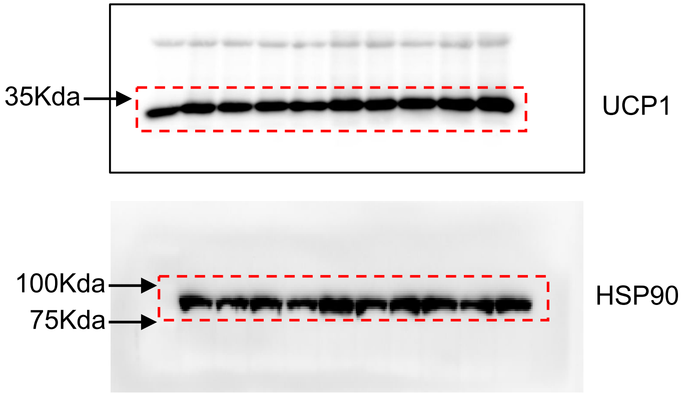

Supplemental Figure. 15B

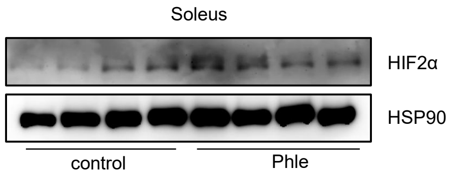

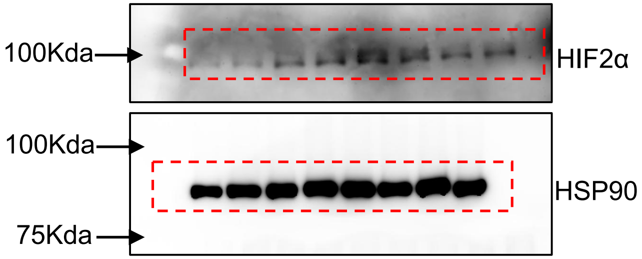

Supplement: Unedited blot and gel images [file jci-136-195411-s097.pdf]
